# Whole‐body endothermy: ancient, homologous and widespread among the ancestors of mammals, birds and crocodylians

**DOI:** 10.1111/brv.12822

**Published:** 2021-12-10

**Authors:** Gordon Grigg, Julia Nowack, José Eduardo Pereira Wilken Bicudo, Naresh Chandra Bal, Holly N. Woodward, Roger S. Seymour

**Affiliations:** ^1^ School of Biological Sciences University of Queensland Brisbane QLD 4072 Australia; ^2^ School of Biological and Environmental Sciences Liverpool John Moores University James Parsons Building, Byrom Street Liverpool L3 3AF U.K.; ^3^ School of Earth, Atmospheric and Life Sciences University of Wollongong Wollongong NSW 2522 Australia; ^4^ School of Biotechnology KIIT University Bhubaneswar 751024 India; ^5^ Oklahoma State University Center for Health Sciences Tulsa OK 74107 U.S.A.; ^6^ School of Biological Sciences University of Adelaide Adelaide SA 5005 Australia

**Keywords:** endothermy, evolution, temperature regulation, non‐shivering thermogenesis, tachymetabolism, plesiomorphy, UCP1, brown adipose tissue, amniote heart

## Abstract

The whole‐body (tachymetabolic) endothermy seen in modern birds and mammals is long held to have evolved independently in each group, a reasonable assumption when it was believed that its earliest appearances in birds and mammals arose many millions of years apart. That assumption is consistent with current acceptance that the non‐shivering thermogenesis (NST) component of regulatory body heat originates differently in each group: from skeletal muscle in birds and from brown adipose tissue (BAT) in mammals. However, BAT is absent in monotremes, marsupials, and many eutherians, all whole‐body endotherms. Indeed, recent research implies that BAT‐driven NST originated more recently and that the biochemical processes driving muscle NST in birds, many modern mammals and the ancestors of both may be similar, deriving from controlled ‘slippage’ of Ca^2+^ from the sarcoplasmic reticulum Ca^2+^‐ATPase (SERCA) in skeletal muscle, similar to a process seen in some fishes. This similarity prompted our realisation that the capacity for whole‐body endothermy could even have pre‐dated the divergence of Amniota into Synapsida and Sauropsida, leading us to hypothesise the homology of whole‐body endothermy in birds and mammals, in contrast to the current assumption of their independent (convergent) evolution. To explore the extent of similarity between muscle NST in mammals and birds we undertook a detailed review of these processes and their control in each group. We found considerable but not complete similarity between them: in extant mammals the ‘slippage’ is controlled by the protein sarcolipin (SLN), in birds the SLN is slightly different structurally and its role in NST is not yet proved. However, considering the multi‐millions of years since the separation of synapsids and diapsids, we consider that the similarity between NST production in birds and mammals is consistent with their whole‐body endothermy being homologous. If so, we should expect to find evidence for it much earlier and more widespread among extinct amniotes than is currently recognised. Accordingly, we conducted an extensive survey of the palaeontological literature using established proxies. Fossil bone histology reveals evidence of sustained rapid growth rates indicating tachymetabolism. Large body size and erect stature indicate high systemic arterial blood pressures and four‐chambered hearts, characteristic of tachymetabolism. Large nutrient foramina in long bones are indicative of high bone perfusion for rapid somatic growth and for repair of microfractures caused by intense locomotion. Obligate bipedality appeared early and only in whole‐body endotherms. Isotopic profiles of fossil material indicate endothermic levels of body temperature. These proxies led us to compelling evidence for the widespread occurrence of whole‐body endothermy among numerous extinct synapsids and sauropsids, and very early in each clade's family tree. These results are consistent with and support our hypothesis that tachymetabolic endothermy is plesiomorphic in Amniota. A hypothetical structure for the heart of the earliest endothermic amniotes is proposed. We conclude that there is strong evidence for whole‐body endothermy being ancient and widespread among amniotes and that the similarity of biochemical processes driving muscle NST in extant birds and mammals strengthens the case for its plesiomorphy.

## I INTRODUCTION

### (1) Stimulus for this research

Long‐held opinion is that endothermy evolved independently in birds and mammals (e.g. Ruben, 1995; Poelmann *et al*., 2014; Padian & de Ricqlès, 2020), but several recent studies have led us to propose that their whole‐body, high‐intensity (tachymetabolic) endothermy may actually share a common ancestry. Recent studies (Rowland, Bal & Periasamy, 2015b; Nowack *et al*., 2017) have put forward a well‐supported hypothesis that the ancient and still ubiquitous source of regulatory non‐shivering thermogenesis (NST – metabolic heat production without muscular contraction) in mammals resides in their skeletal muscle. Hitherto the only source of NST in mammals was thought to be from brown adipose tissue (BAT) (Cannon *et al*., 2000; Cannon & Nedergaard, 2004). This has been problematic because (as detailed below) BAT does not occur in either marsupials or monotremes, and it is absent in many eutherians (Nowack *et al*., 2017). Interestingly, the skeletal muscle NST hypothesis aligns all groups of mammals with birds, whose source of NST is also thought to be in skeletal muscle (Bicudo, Vianna & Chaui‐Berlinck, 2001; Bicudo, Bianco & Vianna, 2002), and in both groups evidence suggests that the regulatory NST is derived from the ‘slippage’ of Ca^2+^ from the sarcoplasmic reticulum Ca^2+^‐ATPase (SERCA). This mechanism was described in rabbits (*Oryctolagus cuniculus*) by de Meis (2001), and de Meis, Arruda & Carvalho (2005) speculated about its “probable” importance to “facultative thermogenesis”. But its significance for thermoregulation was overlooked until experiments on mice (*Mus musculus*) by Bal *et al*. (2012) showed that the mechanism is adaptive in mammals, being upregulated in response to cold challenge and controlled by the protein sarcolipin (SLN). Collectively, these observations generated our central hypothesis, that birds and mammals share a common ancestry for their tachymetabolic endothermy, a proposal that has important implications for current ideas about vertebrate endothermy and its evolution.

One of the implications of this proposal is that there should be very early occurrences of tachymetabolic endothermy in the amniote family tree. Accordingly, drawing on recent studies in biochemistry, comparative physiology and palaeontology, we explore that possibility and conclude that whole‐body, tachymetabolic endothermy is very much older and more widespread than has been recognised previously and that its many occurrences among extinct and extant amniotes are homologous.

### (2) Definitions

The language of the biology of thermoregulation is often confusing and commonly ambiguous, so here we explain how we define the terms used herein. The literal meaning of *ectothermy* is ‘heat from outside’, in contrast to *endothermy* meaning ‘heat from inside’. Most invertebrates are *ectotherms*, and so are most extant reptiles, amphibians and fishes. Typically, ectothermy is characterised by low metabolic rate (*bradymetabolism*) and little thermal insulation. Metabolic heat production is so low that it has little effect on body temperature (*T*_b_), and ectotherms typically have almost the same *T*_b_ as their environment except that many can regulate it by behavioural manipulation of heat exchange with the environment, for example by basking in the sun. Some ectotherms show *regional endothermy*. For example, marlin and swordfish warm their eyes with modified muscles, some bony fishes and sharks employ an elaborate countercurrent blood system in the lateral musculature to retain the by‐product heat from swimming, while leatherback turtles (*Dermochelys coriacea*) retain it by being globose and well‐insulated. Even some pythons curl elegantly around their eggs and warm them and themselves by shivering. These ectotherms exhibit internal warming, mostly localised, but metabolically they are bradymetabolic. Extant birds and mammals, however, are *tachymetabolic endotherms* (*whole‐body endotherms*) and, except when they are in torpor or hibernation, they are characterised by basal metabolic rates 5–10 times higher than ectotherms (*tachymetabolism*), and at rest they produce enough ‘waste’ heat from the aerobic metabolism of their internal organs to raise *T*_b_ above ambient. Within the so‐called ‘thermal neutral zone’ (TNZ, a range of ambient temperature, *T*_a_, in which resting metabolic rate is independent of *T*_a_), they regulate *T*_b_ by controlling the rate of heat loss with adjustments of the insulative properties of their integument, surface blood flow and evaporation. At cold *T*_a_, below their TNZ, basal metabolic rate is insufficient to maintain a stable *T*_b_ and heat production can be increased by exercise, shivering thermogenesis (ST) and by NST. Most tachymetabolic endotherms balance heat production and heat loss to maintain *T*_b_ within a narrow range, and this pattern is commonly described as *homeothermic* (literally ‘same heat’) *endothermy*. However, tachymetabolic endothermy should not be seen as a strategy to effect thermoregulation. Rather, it is characterised by its tachymetabolism, which permits a high‐energy lifestyle, with enough energy to provide a capacity for physiological thermoregulation. Importantly, the maintenance of homeothermy is not a defining characteristic of tachymetabolic endothermy; many ‘normally tachymetabolic’ and ‘normally homeothermic’ endotherms can lower their metabolic rate below basal, abandon their stable *T*_b_ and enter torpor, daily or for longer periods, to conserve energy or to avoid harsh environmental conditions (Ruf & Geiser, 2015). These may be referred to as *heterothermic endotherms*. Because the word endothermy used alone can be ambiguous, we will use it with an appropriate clarifying qualifier, such as ‘regional endothermy’, ‘whole‐body endothermy’ (or ‘*tachymetabolic*’ *endothermy*), unless context makes that unnecessary.

### (3) Historical background

The source of NST in mammals has long been accepted and promoted as resulting solely from the activity of the uncoupling protein UCP1, located within deposits of BAT (e.g. Cannon *et al*., 2000; Cannon & Nedergaard, 2004). Indeed, Cannon *et al*. (2000, p. 387) claimed that “there is no other mammalian non‐shivering thermogenesis than that emanating from adrenergic stimulation of brown adipose tissue”. But BAT is far from ubiquitous among extant mammals. Neither BAT nor UCP1 occurs in either monotremes or marsupials (Augee, 1978; Hayward & Lisson, 1992; Rose *et al*., 1999; Kabat *et al*., 2003a; Kabat, Rose & West, 2003b; Polymeropoulos, Jastroch & Frappell, 2012) and neither is BAT found in all eutherian (placental) mammals (Rothwell & Stock, 1985; Gaudry *et al*., 2017; Nowack *et al*., 2019), yet all are tachymetabolic endotherms. BAT does occur in many, mostly small eutherians (Saito, Saito & Shingai, 2008) and particularly those that undergo torpor and/or hibernation in very cold climates, for which BAT has come to be regarded as necessary (Cannon *et al*., 2000; Nowack *et al*., 2017). NST is also recognised as the significant heat source for arousal from torpor or hibernation. However, BAT is not an obligate requirement for arousal from torpor or hibernation in all mammals because, despite lacking BAT, a number of marsupials and at least one monotreme show torpor and/or hibernation and are able to rewarm to normothermia (Geiser, 1988; Grigg, Beard & Augee, 1989, 2004; Grigg, Augee & Beard, 1992a; Grigg & Beard, 2000; Grigg, 2004; Nicol & Andersen, 2008; Geiser & Körtner, 2010; Nicol, 2017).

The lack of BAT and thermogenic UCP1 in monotremes, marsupials and so many eutherians has been a puzzle. These mammals are tachymetabolic endotherms, some with a demonstrated capacity for NST, and NST is probably characteristic throughout (see Section II.1), but what is its source? Early indications of an answer came from a Tasmanian marsupial, the bettong (*Bettongia gaimardi*) which, like all marsupials examined so far, lacks BAT. Working on the effect of catecholamines on bettongs, Ye *et al*. (1995, 1996) found that NST increased in response to norepinephrine, a hormone used to indicate its occurrence and now identified as being a likely requirement for the recruitment of muscle NST (Rowland *et al*., 2015a). The studies by Ye *et al*. (1995, 1996) suggested that whole‐body thermogenesis in bettongs “probably originates in skeletal muscle” (Ye *et al*., 1996, p. R592). Eldershaw *et al*. (1996, p. 315) suggested that “The evolutionary appearance of BAT may have been due to the requirement of a supplementary thermogenic mechanism in juvenile and smaller mammals”. Further work on bettongs and another marsupial, the Tasmanian devil (*Sarcophilus harrisii*), reinforced the suggestion that skeletal muscle is involved. Both species are homeothermic endotherms lacking both BAT and UCP1 (Rose *et al*., 1999; Kabat *et al*., 2003a,b) and both show norepinephrine‐stimulated NST. Bettongs and Tasmanian devils are only distantly related, so NST is probably typical of marsupials. The occurrence of NST has not yet been demonstrated in monotremes, but is likely: Grigg *et al*. (1992a) observed arousal from 12°C to 35°C by a large, short‐beaked echidna (*Tachyglossus aculeatus*) dug from its field hibernaculum. *T*_b_ rose with only very slow movements of the limbs and body to 18°C, at which occasional body twitches began, and shivering above 20°C steepened the rate of *T*_b_ rise.

Birds too lack BAT (Johnston, 1971; Saarela *et al*., 1991; Brigham & Trayhurn, 1994; Emre *et al*., 2007) and their source of NST is also considered to be within skeletal muscle (Bicudo *et al*., 2001, 2002). Noting these findings, and the large proportion of body mass contributed by skeletal muscle, Grigg *et al*. (2004, p. 991) observed that “It seems likely that a widespread, ancient, and controllable mechanism of regulatory NST will be found in the skeletal muscle of mammals” and suggested that one possible “source could be in the sarcoplasmic reticulum, as in birds”.

The puzzle seems now to be resolved from studies by Rowland *et al*. (2015b) and Nowack *et al*. (2017), which provide a convincing explanation. These authors proposed that the primary, ancient source of NST in all mammals is indeed located within skeletal muscle and is derived from it by modification of the activity of the sarcoplasmic reticulum Ca^2+^‐ATPase (SERCA), a calcium‐pump involved in muscle relaxation, uncoupled from calcium transport by SLN when producing heat (Bal *et al*., 2012, 2016). This proposal is not yet widely accepted, but considerable evidence for it in mammals is accumulating (see Section II.1). Excitingly, the biochemical mechanism driving muscle NST in mammals appears to be very similar to the mechanism posited to be the source of NST in the skeletal muscle of birds, because that too is thought to be SERCA based (Bicudo *et al*., 2001, 2002). Discussing the suggestion that the ‘missing’ source of NST in many mammals might be in skeletal muscle, Grigg *et al*. (2004, p. 991), paraphrasing Grigg *et al*. (2004), observed that “If it turns out to be the same mechanism in both birds and mammals, that could imply that endothermy in these two groups stems from a common origin”; that hypothesis is the main focus of this review.

### (4) Outline

Our deliberations comprise a reappraisal of the nature and timing of the occurrences of tachymetabolic endothermy within the Amniota, particularly whether its occurrences in synapsids and sauropsids have evolved independently as currently assumed (e.g. Poelmann *et al*., 2014), or are homologous as a similarity between their skeletal muscle sources of NST may suggest. Similarity would be consistent with a common origin; dissimilarity would favour convergent evolution. Accordingly, our first aim was to explore the extent of similarity between the molecular basis of muscle NST in extant birds and mammals. Second, if muscle NST is found to be similar in both groups, consistent with them sharing a common origin, the capacity for expression of tachymetabolic endothermy may be older than the divergence of Amniota into Synapsida and Sauropsida. If so, it could have appeared earlier and been more widespread among amniotes than is usually thought. Our second aim therefore was to carry out a substantial review of relevant studies on amniote fossil material with that prediction in mind (see online Supporting Information, Appendix S1). Finally, we conclude that there is a great deal of evidence consistent with our hypothesis that tachymetabolic endothermy in amniotes is plesiomorphic, discuss the most likely counterarguments, review the implications of this conclusion, and propose an hypothetical structure for the heart of the earliest tachymetabolic endotherms.

## II HOW SIMILAR ARE THE MOLECULAR MECHANISMS OF MUSCLE NST IN BIRDS AND MAMMALS?

Recent studies have described an important role for skeletal muscle as a source of NST in the thermoregulation of extant mammals and birds. Here and in Table 1 we compare and contrast the molecular mechanisms driving this in each group.

**Table 1:** Comparison of molecular processes contributing to an increase in non‐shivering thermogenesis (NST) in response to cold exposure in extant eutherian and non‐eutherian mammals and birds. Insufficient data are available from monotremes, so they are combined with marsupials. The table identifies gaps in knowledge

		Mammals		Birds
Characteristics and molecular mechanisms		Eutherians	Marsupials and monotremes	
Brown adipose tissue (BAT) and UCP1				
1.	Presence of BAT	Typically present^1^, many exceptions^2–5^	BAT absent^4,6–14^	BAT absent^14–17^
2.	UCP1 expression in mitochondria	Thermogenic UCP1 only in eutherians with BAT^1,18^	Thermogenic UCP1 absent from both^13,19–21^	UCP1 gene not present in birds (or crocodiles)^22^
Skeletal muscle response or adaptation to cold				
3.	Shivering	In acute cold exposure; suppressed by adaptation^23,24^	In acute cold exposure; suppressed by adaptation^25^	In acute cold exposure^26–28^
4.	Oxidative capacity and oxygen consumption	Increase in mitochondrial abundance and oxidative capacity ^29^	Increase in mitochondrial abundance and oxidative capacity ^11,25,30^	Increase in mitochondrial abundance and oxidative capacity^31,32^
5.	Redness (indication of mitochondrial abundance and blood flow)	Increase^33,34^	Not known	Increase^31^
6.	Mitochondrial abundance	Increase^35,36^	Increase^30^	Increase^37,38^
7.	Mitochondrial Ca^2+^ uptake	Increase^39^	Not known	Increase^40^
8.	Electron transport chain (ETC) proteins and ATP synthetase	Increase^35,41^	Not known	Increase^31^
9.	Adenine nucleotide translocator (ANT) expression/activity (either produces heat or transports ATP from mitochondria to cytosol, supporting heat production by SERCA and/or myosin)	Increase^42^	Increase ^43^	Increase^44,45^
10.	RyR and SERCA expression	Increase^41,46^	Not known	Increase^47,48^
11.	SLN expression	Increase^41,36^	Not known	Not known
12.	UCP3 (in mammals); avUCP (avian homolog) (produce heat directly, or scavenge ROS, helping mitochondrial ATP synthesis)	Increase^49,50^	Increase^30^	Increase^38,51^
13.	PGC1α expression and mitochondrial biogenesis	Increase^52^	Not known	Increase^31,53^
14.	Thyroid hormone in blood	Increase^33^	Increase^25^	Increase^54^
15.	Glucocorticoids in blood	Increase^55^	Not known	Increase^56^

PGC1α, peroxisome‐proliferator‐activated receptor‐γ co‐activator 1α; ROS, reactive oxygen species; RyR, ryanodine receptor; SERCA, sarcoplasmic reticulum Ca^2+^‐ATPase; SLN, sarcolipin; UCP1, uncoupling protein 1.

^1^Cannon & Nedergaard (2004); ^2^Rothwell & Stock (1985); ^3^Fyda *et al*. (2020); ^4^Gaudry, Campbell & Jastroch (2018); ^5^Nowack *et al*. (2019); ^6^Rowlatt, Mrosovsky & English (1971); ^7^Augee, Gooden & Musser (2006); ^8^Augee (1978); ^9^Dawson (1989); ^10^Hayward & Lisson (1992); ^11^Schaeffer, Villarin & Lindstedt (2003); ^12^Saito *et al*. (2008); ^13^Polymeropoulos *et al*. (2012); ^14^Jastroch, Oelkrug & Keipert (2018); ^15^Johnston (1971); ^16^Saarela *et al*. (1991); ^17^Brigham & Trayhurn (1994); ^18^Nedergaard & Cannon (2018); ^19^Kabat *et al*. (2003b); ^20^Oelkrug, Polymeropoulos & Jastroch (2015); ^21^Holloway & Geiser (2001); ^22^Schwartz, Murray & Seebacher (2008); ^23^Silva (2006); ^24^Block (1994); ^25^Rose & Kuswanti (2004); ^26^Blix (2016); ^27^Barré *et al*. (1989); ^28^Chaffee & Roberts (1971); ^29^Matoba & Murakami (1981); ^30^Schaeffer *et al*. (2005); ^31^Hirabayashi *et al*. (2005); ^32^Ijiri *et al*. (2009); ^33^Arruda *et al*. (2008); ^34^Rowland *et al*. (2015a); ^35^Cheah *et al*. (1985); ^36^Bal *et al*. (2017); ^37^Sirsat *et al*. (2016); ^38^Raimbault *et al*. (2001); ^39^Greenway & Himms‐Hagen (1978); ^40^Barré & Nedergaard (1987); ^41^ Bal *et al*. (2016); ^42^Mollica *et al*. (2005); ^43^Jastroch *et al*. (2009); ^44^Toyomizu *et al*. (2002); ^45^Talbot *et al*. (2004); ^46^Pant, Bal & Periasamy (2016); ^47^Dumonteil, Barré & Meissner (1993); ^48^Dumonteil, Barré & Meissner (1995); ^49^Shabalina *et al*. (2010); ^50^Simonyan *et al*. (2001); ^51^Rey *et al*. (2010); ^52^Puigserver *et al*. (1998); ^53^Ueda *et al*. (2005); ^54^Rudas & Pethes (1984); ^55^Hashimoto *et al*. (1970); ^56^de Bruijn & Romero (2011).

### (1) Non‐shivering thermogenesis (NST) in mammalian skeletal muscle

**Fig. 1:**
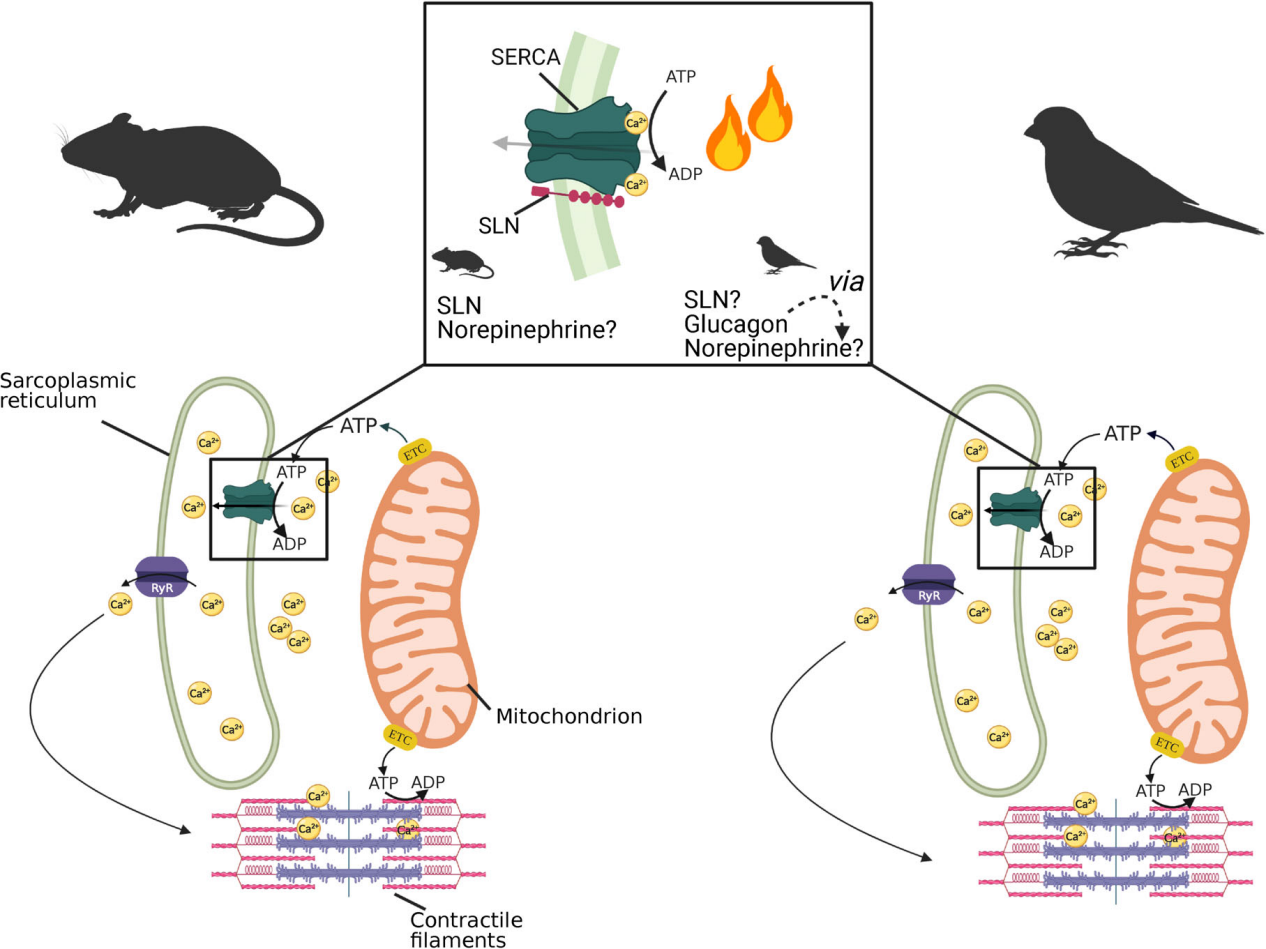
Mechanisms of muscle non‐shivering thermogenesis (NST) in mammals and birds. The sarcoplasmic reticulum Ca^2+^‐ATPase (SERCA) is a Ca^2+^ pump involved in muscle contraction and thermogenesis. During the contraction–relaxation cycle (outside the box) SERCA uses ATP to pump Ca^2+^ from the cytosol into the sarcoplasmic reticulum (SR), which leads to muscle relaxation; Ca^2+^ leaves the SR through ryanodine receptor (RyR) channels causing muscle contraction. The boxed region relates to the shift to muscle NST in mammals and birds. In mammals the shift to NST is prompted by the attachment of a protein, sarcolipin (SLN) to SERCA, increasing its ATP utilisation by causing Ca^2+^ ‘slippage’, i.e. Ca^2+^ attaches to SERCA, but instead of being transported into the SR, Ca^2+^ is released again at the cytosolic side, producing heat from the ATP hydrolysis without actual muscle contraction. There is evidence of strong similarity between the mechanisms of muscle NST in birds and mammals (see Section II and Table 1), although the physiological role of SLN in bird muscle NST has not yet been demonstrated. Furthermore, how SLN or its equivalent in birds is recruited is still not fully understood. In mammals, data suggest the involvement of norepinephrine; this may be the case in birds too: there is evidence from ducklings of norepinephrine levels increasing in response to injection of glucagon (Filali‐Zegzouti *et al*., 2005). Figure derived from Bal *et al*. (2021). ETC, Electron Transport Chain.

During the contraction of skeletal muscle in mammals the Ca^2+^‐ATPase pump uses ATP to pump Ca^2+^ from the cytosol into the sarcoplasmic reticulum (SR) in which Ca^2+^ is stored, leading to muscle relaxation. The stored Ca^2+^ leaves the SR again through ryanoid receptor (RyR) channels during the next depolarisation event, causing muscle contraction. When SERCA functions in mammalian thermogenesis, its activity is increased by the protein sarcolipin (SLN) (Fig. 1). Sarcolipin causes Ca^2+^ ‘slippage’, i.e. Ca^2+^ attaches to SERCA, but instead of being transported into the SR, Ca^2+^ is released again at the cytosolic side (Smith *et al*., 2002; Bal *et al*., 2012). Thus, heat is produced without muscle contraction. The proposal that SLN‐regulated cycling of Ca^2+^ in muscle accounts for NST in mammals lacking BAT is not without controversy. Indeed, Jastroch, Polymeropolous & Gaudry (2021) mentioned SERCA only in the context of its possible function in birds, referencing a critical review by Campbell & Dicke (2018). They also noted the experimental challenges involved in establishing ‘muscle NST mechanisms’ as significant contributors to systemic thermoregulation, particularly because NST and ST both occur in the same cell. Campbell & Dicke (2018) expressed doubt about the capacity of the SLN‐mediated NST mechanism to account for the quantum of NST observed in mammals lacking BAT. However, studies from several different groups have provided evidence for a role of SLN in thermogenesis and muscle metabolism (Maurya *et al*., 2018; Rotter *et al*., 2018; Kaspari *et al*., 2020; Nicolaisen *et al*., 2020; Wang *et al*., 2020). Also, the case for the muscle NST hypothesis has been strengthened considerably by recent experimental work on wild boar piglets (*Sus scrofa*), eutherian mammals that lack BAT (Nowack *et al*., 2019). These authors monitored an age‐related replacement of ST by NST in wild piglets, which lack BAT, and found the transition to be accompanied by an increase in *T*_b_ and coincident increase in SERCA activity and the expression of both SERCA and SLN. Within eutherian mammals, muscle NST has so far been identified in rodents, rabbits (reviewed by Nowack *et al*., 2017) and wild boar (Nowack *et al*., 2019) and it is assumed to be present in all mammalian species, either as a primary heat‐production mechanism or, in many eutherians (particularly small ones), in addition to the heat produced by BAT (Rowland *et al*., 2015b; Nowack *et al*., 2017).

Importantly, modification of SERCA for NST in muscle is adaptive in eutherian mammals: SLN expression in the skeletal muscle is upregulated when mice are adapted to cold (Bal *et al*., 2016) and in wild boar piglets exposed to cold spring weather after birth (Nowack *et al*., 2019). SLN upregulation is even more pronounced in mice with compromised BAT function (Bal *et al*., 2016, 2017). Further, SLN expression is tuned to cold adaptation in developing neonatal rodents (Pant *et al*., 2016) and it also seems to play a role in the final stages of rewarming by hibernating squirrels (*Ictidomys tridecemlineatus*) (Anderson, 2016; Oliver *et al*., 2019). Together, these findings show that SLN is critical for muscle NST in rodents and is possibly even more important in species lacking BAT and UCP1 (Nowack *et al*., 2019), such as monotremes, marsupials (Hayward & Lisson, 1992; Rose *et al*., 1999; Kabat *et al*., 2003a,b; Polymeropoulos *et al*., 2012) and certain large‐bodied species (Gaudry *et al*., 2017). The evolution of thermogenic BAT and UCP1 must have occurred after the divergence of eutherians and marsupials (Saito *et al*., 2008), implying that endothermy in non‐eutherian mammals relied on muscle NST, as in birds.

### (2) Non‐shivering thermogenesis (NST) in avian skeletal muscle

Much less is known about the details of muscle NST in birds, but evidence suggests that birds too rely on SERCA for both muscle contraction and, with Ca^2+^ transport uncoupled, for NST also. Dawson & Carey (1976) first reported an increase in the thermogenic capacity and endurance of cold‐exposed birds, and the involvement of muscle NST was demonstrated by Grav *et al*. (1988). This work on the common eider (*Somateria mollissima*), a seabird that hatches in the Arctic, revealed that mitochondrial oxidative capacity in muscle increases after hatching, but not in the liver. Skeletal muscle is the major site of NST in other cold‐acclimated bird species too, including king penguins (*Aptenodytes patagonicus*) (Duchamp *et al*., 1991) and Muscovy ducks (*Cairina moschata*) (Duchamp & Barré, 1993; Marmonier *et al*., 1997). Additionally, cold‐exposed ducklings show an increase in expression of two isoforms of SERCA (SERCA1a, SERCA2a) and RyR (Dumonteil *et al*., 1995). Despite these similarities, details of the exact mechanism remain elusive. Particularly, although birds have a SLN sequence similar to that of mammals (Bal *et al*., 2018), differences in the C‐ and N‐terminal sequences lead to questions about its regulatory role on SERCA (Montigny *et al*., 2014), and the physiological role of SLN in bird muscle NST has not yet been demonstrated. In mammals, the N‐terminal sequences of SLN are more important for the uncoupling of SERCA, while C‐terminal and transmembrane sequences are critical for its correct positioning and co‐localisation in the groove of SERCA inside the SR (Sahoo *et al*., 2015). It is assumed that the N‐terminus of SLN positions itself in the loop regions of SERCA on the cytoplasmic side, regulating SLN–SERCA interaction. Importantly, a comparison of SERCA sequences from birds and mammals shows that the residues in the loop regions of SERCA that can potentially interact with N‐terminal residues of SLN are also not conserved in birds (Bal *et al*., 2018). Thus, avian SLN may facilitate a better uncoupling with avian SERCA and a role of avian SLN in muscle NST cannot be ruled out.

A study on cold‐exposed Muscovy ducklings showed that muscle NST is correlated with upregulation of the avian uncoupling protein (avUCP), which, in contrast to mammalian UCP1, is not associated with a change in mitochondrial membrane proton conductance (Teulier *et al*., 2010). Further, Teulier *et al*. (2014) found that ducklings reared at thermoneutrality were able to display a capacity for NST, based on simultaneous measurements of muscle electromyogram activity and resting metabolic rates during the first month of their post‐hatching life. These authors also reported that this thermogenic mechanism increased further in ducklings chronically exposed to a cold environment but decreased over time when birds were kept in a thermoneutral environment. Adenine nucleotide translocator (ANT) in muscle has been proposed as another potential uncoupling protein in avian NST (Roussel *et al*., 2000; Talbot *et al*., 2004; Walter & Seebacher, 2009). Interestingly, upregulation of ANT expression in muscle upon cold adaptation is observed not only in birds but also in mammals and seems to be highly correlated with the patterns of expression of SLN and SERCA in rodents undergoing cold adaptation (Bal *et al*., 2016). Moreover, ANT is the protein serving to transport ATP out of mitochondria (Klingenberg, 2008; Kunji *et al*., 2016) and ANT levels increase when cellular ATP demand becomes higher (Bal *et al*., 2016). Thus, despite NST *via* ANT or SERCA being seen by some as alternative options (e.g. Ruuskanen, Hsu & Nord, 2021) they may actually be synergistic, and it could be that ANT acts as a facilitator of SERCA‐based muscle NST.

Despite overall similarity between birds and mammals, a recent study looking at the gene expression of SLN in adult juncos (*Junco hyemalis*) exposed to extreme cold (−8°C) found downregulation of the expression of SLN, while many genes involved in Ca^2+^ cycling and known to be altered by cold adaptation were unrepresented in their data (Stager & Cheviron, 2020). As mentioned above, the involvement of SLN in muscle NST is not yet proved for birds, but if SLN is involved this could mean that muscle NST was not sufficient for heat production at this temperature and that the birds had to rely on shivering. However, it needs to be noted that the study analysed only the pectoralis, a muscle that is used in birds primarily for shivering, and that SLN and Ca^2+^‐handling proteins in other posture‐bearing muscles that are recruited for NST in mammals have not been studied.

### (3) Fuelling and regulation of skeletal muscle NST: birds *versus* mammals

Because metabolic processes are highly conserved among vertebrates (Smith & Morowitz, 2004; Seebacher, 2018), the compounds necessary to activate and fuel the process of NST by the target thermogenic organ (muscle or muscle plus BAT in many eutherians) likely share common metabolic pathways in mammals and birds, perhaps with quantitative differences between them rather than *de novo* structures or processes. We describe some of the shared traits found in birds and mammals below.

#### (a) NST is fuelled by fatty acids in both birds and mammals

Muscle NST relies primarily on energy from ATP and the production of ATP by mitochondria plays a critical role, so continued heat production *via* SERCA depends upon the supply of substrates to mitochondria. In both groups this seems to rely largely on fatty acids. In chickens (*Gallus gallus domesticus*), Toyomizu *et al*. (2002) showed that cold acclimation (4–6°C, 10–12 days) induced fatty acid‐mediated uncoupling of mitochondrial oxidative phosphorylation processes and also increased the rate of ATP synthesis in the mitochondria of skeletal muscle. Interestingly, fatty acid oxidation also appears to fuel flight in most avian taxa (Tucker, 1968, 1972; Torre‐Bueno & Larochelle, 1978; Hudson & Bernstein, 1983; Ward *et al*., 2001; Bundle, Hansen & Dial, 2007), providing a fuel source with the greatest energy density for the least weight (Jenni & Jenni‐Eiermann, 1998; Guglielmo, 2010). Fatty acids seem to be an important substrate for muscle NST in mammals too. Increased mitochondrial content and proteins associated with fatty acid utilisation in muscle have been shown in cold‐acclimated mice, rats (*Rattus rattus*) and rabbits (Mollica *et al*., 2005; Bruton *et al*., 2010; Bal *et al*., 2016, 2017). By contrast, mammals and birds differ in the fuel source driving shivering. In mammals, shivering relies mainly on glucose (Haman & Blondin, 2017), whereas birds are thought to rely on lipids (Swanson, 2010; Zhang *et al*., 2015).

#### (b) Transcriptional regulation of NST is similar in mammals and birds

Another apparent similarity between birds and mammals is that the protein peroxisome‐proliferator‐activated receptor‐γ co‐activator‐1α (PGC‐1α), or a highly homologous protein, appears to play a central role in the transcriptional regulation of energy metabolism in both, which is important for NST (Puigserver & Spiegelman, 2003; Walter & Seebacher, 2009). In eutherian mammals PGC‐1α coordinates a variety of transcription factors and nuclear receptors involved in energy metabolism, and its expression in skeletal muscle is very responsive to cold adaptation (Puigserver *et al*., 1998; Goto *et al*., 2000; Baar *et al*., 2002; Taylor *et al*., 2005; Terada *et al*., 2005; Stancic *et al*., 2013). Avian UCP transcripts in skeletal muscles of chickens increase following cold exposure (Toyomizu *et al*., 2002), suggesting transcriptional regulation similar to mammals. Furthermore, chicken DNA encodes a protein homologous to mammalian PGC‐1α, which shows alterations in transcript levels in skeletal muscle in response to cold exposure (Ueda *et al*., 2005).

#### (c) Hormonal regulation of muscle NST in birds and mammals

The comparatively recent recognition of the importance of muscle NST means that little is known about its regulation, but catecholamines and thyroid hormones are among the likely candidates. However, what can be gleaned from the literature suggests that regulatory mechanisms of muscle NST are likely to be similar in birds and mammals.

It seems likely that the catecholamine norepinephrine may have a role in the regulation of muscle NST in all mammals and also in birds. Several studies have shown that norepinephrine has a role in the cold adaptation of dogs (*Canis familiaris*), rodents and rabbits (Jansky, 1973). Elevated levels of plasma norepinephrine were observed in UCP1‐knockout mice upon cold acclimatisation, hyper‐recruiting SLN and muscle NST (Rowland *et al*., 2015a). As reported in Section I.3, norepinephrine stimulated NST in two distantly related marsupials, bettongs (Ye *et al*., 1995, 1996) and Tasmanian devils (Kabat *et al*., 2003a). It seems that adrenergic signalling may be required for the recruitment of muscle NST in all mammals, with and without BAT. Norepinephrine seems likely to regulate muscle NST in birds as well, but this has yet to be proved. However, glucagon is known to have thermogenic effects in Muscovy ducklings (Filali‐Zegzouti *et al*., 2000) and an increase in plasma norepinephrine following glucagon injection suggests that the thermogenic response in birds also may be mediated by norepinephrine (Filali‐Zegzouti *et al*., 2005) (Fig. 1).

Thyroid hormones are significantly involved in the thermoregulation of both birds and mammals (Little & Seebacher, 2014). They have profound effects on the metabolism of both skeletal muscle and brown fat in eutherian mammals (Bianco *et al*., 2005; Laurberg, Andersen & Karmisholt, 2005; Arruda *et al*., 2008; Louzada *et al*., 2014) and likely have similar effects in marsupials (Withers & Hulbert, 1988; Rose & Kuswanti, 2004) and monotremes (Nicol, 2017). They also affect several proteins associated with Ca^2+^ cycling in skeletal muscle of laboratory rodents, including both SERCA and SLN (Simonides *et al*., 2001; Minamisawa *et al*., 2006; Trivieri *et al*., 2006). In birds, thyroid hormones are thought to influence both embryogenic development and phenotype (DuRant *et al*., 2014) and are involved in stimulating thermogenesis in chicks (Walter & Seebacher, 2009).

### (4) Biochemical similarities are consistent with a common origin for tachymetabolic endothermy in birds and mammals

Considering the multi‐millions of years since synapsids and sauropsids diverged, the similarity between the biochemical processes driving muscle NST in extant mammals and birds (Table 1) is consistent with our hypothesis that the tachymetabolic endothermy characteristic of birds and most mammals is homologous. With the conservative nature of vertebrate evolution and widespread homologies (Romer & Parsons, 1977), extending to their basic metabolic processes (Smith & Morowitz, 2004), the similarity between birds and mammals in their muscle NST production provides no proof by itself. However, if endothermy arose independently in birds and mammals, as currently accepted, much larger differences would be expected.

## III HEAT PRODUCTION IN MUSCLE OF PRE‐AMNIOTE VERTEBRATES AND PERHAPS EXTANT REPTILES

With homology such a feature across vertebrates, it is no surprise that the molecular mechanisms proposed to account for NST in birds and most, perhaps all, mammals may share antecedents in amniote ancestry. With respect to this, it is of interest to examine extant fishes. Whole‐body endothermy is very challenging for gill breathers, yet several groups of extant fishes show regional endothermy and in some of these heat production is SERCA‐based. Swordfish (Xiphiidae), marlin (Istiophoridae) and butterfly mackerel (*Gasterochisma melampus*) possess cranial heater organs, specialised tissue adjacent to the eyes, derived from muscle (Block, 1994; Block & Finnerty, 1994). The heat raises eye temperature by some degrees above that of the surrounding water, thereby enhancing temporal resolution of vision (Carey, 1982; Fritsches, Brill & Warrant, 2005). The mechanism of heat production is *via* an increase in SERCA activity, but while SLN in mammals and birds leads to an uncoupling of SERCA activity and Ca^2+^ transport, heat in fish is generated *via* futile cycling of Ca^2+^. In fish, RyR channels release Ca^2+^ from the SR, whereas SERCA (in fact the same isoform as that involved in muscle NST in mammals) pumps it back into the organelle, leading to increased heat production *via* an increase in activity (Block, 1994; Morrissette, Franck & Block, 2003; da Costa & Landeira‐Fernandez, 2009). Whether SLN or a different uncoupler is involved is unknown. Heat production is localised within the muscle and does not lead to whole‐body endothermy, and the fish remain bradymetabolic ectotherms. Tunas and some sharks (Lamnidae) employ a countercurrent blood supply to retain by‐product heat in the lateral swimming musculature (Carey & Teal, 1966, 1969; Bernal *et al*., 2005; Sepulveda *et al*., 2007, 2008; Ciezarek *et al*., 2019), benefitting their cruising speeds and migrational ranges (Watanabe *et al*., 2015). Whether this is supplemented by SERCA‐based heat production is unknown but, in contrast to typical ectotherms, the metabolic rate of Pacific Bluefin tuna (*Thunnus orientalis*) is known to increase in cooler water (Blank *et al*., 2007), hinting at possible thermogenesis in response to cold (Ciezarek *et al*., 2019). It is worth noting that some tunas have extensive visceral retes as well (Stevens, 2011). Perhaps the most striking of the regionally endothermic fishes are the opahs (*Lampris* sp.). They maintain much of their body a few degrees above the ambient sea water (Runcie *et al*., 2009; Wegner *et al*., 2015; Davesne *et al*., 2018). They have a complex extraocular *rete mirabile* that is thought to retain heat and warm the eye, a substantial counter‐current vascular arrangement in the gills to retain heat produced in muscles driving the pectoral fins and may also have some form of SERCA activity. Furthermore, a role for SLN in their deep muscle tissues has been proposed recently based on the SLN/SERCA ratio (Franck, Slight‐Simcoe & Wegner, 2019).

SLN occurs also in reptiles (Bal & Periasamy, 2020). No thermogenic role has yet been described, but the possibility may be worth exploring. One species of lizard, the South American tegu, *Tupinambis merianae*, can maintain an increased *T*_b_ independent of *T*_a_ during the breeding period (Tattersall *et al*., 2016) – the source of the heat remains unknown (G.J. Tattersall, personal communication, June 2020). Also among reptiles, some pythons ‘shiver’ to warm their eggs (Hutchison, Dowling & Vinegar, 1966; Harlow & Grigg, 1984), but whether this can be supplemented by SERCA‐based heat production is unknown. Bal & Periasamy (2020) discussed two competing theories to explain the origin of SLN as a regulator of SERCA activity in vertebrates, but their resolution will require further research on non‐eutherian species.

Because of its high specific heat and high thermal conductivity, water poses a challenging environment for the evolution of tachymetabolic endothermy by aquatic vertebrates. This applies to gill‐breathers particularly, for which the relatively low oxygen content in water, compared to that in air, poses an additional challenge. However, despite the constraints, and based on what we see in extant species, selection pressures leading to regional warmth have operated often and among aquatic vertebrates as diverse as bony fishes, sharks and turtles, leading to a diversity of mechanisms that either retain by‐product heat or produce it, and involving SERCA in several cases, with more examples likely to be found. No examples are known from extant ‘Amphibia’, the Lissamphibia, but the first terrestrial amniotes may have carried with them an historical capacity for heat production that could be upregulated in skeletal muscle when the thermal constraints imposed by breathing water were released. This was in combination with the release of another constraint, the much lower oxygen content of water compared with air. The Australian lungfish, *Neoceratodus forsteri*, a lobe‐finned fish related to the line from which tetrapods arose, provides an example of this. Juvenile fish forced into activity achieved a higher metabolic rate when they had access to air than when deprived of it and had to rely on gills alone (Grigg, 1965). Tachymetabolic endothermy is characterised by a high aerobic metabolic intensity and the capacity for production and retention of heat at a whole‐body scale. When air‐breathing amniotes came onto land this became easier for the first time, and the wherewithal for controllable heat production was close at hand.

## IV PROXIES FOR WHOLE‐BODY (TACHYMETABOLIC) ENDOTHERMY IN SAUROPSIDA AND SYNAPSIDA

Our hypothesis that the capacity for tachymetabolic endothermy in birds and mammals is homologous and therefore very old predicts that there should have been early expressions and widespread occurrences of it throughout the amniote phylogeny. Accordingly, we compiled an inventory of its probable occurrences in sauropsids and synapsids, paying particular attention to the earliest clades. Sometimes we had the benefit of conclusions about metabolic status provided by the researchers themselves, but in many studies this was not part of their aim and we made our own interpretation, guided by a number of proxies as discussed below. Biochemical and physiological evidence is almost always lacking in fossils, so we relied on other evidence to indicate tachymetabolism, i.e. sustained high growth rate, activity levels, reconstructed body form and other anatomical evidence of the high metabolic rate characteristic of endothermy, as well as, cautiously, *T*_b_ estimates from palaeothermometry.

In our search of the palaeontology literature, when we found taxa for which there was good evidence of tachymetabolism we scored it as an occurrence in the relevant taxonomic group. However, we do not mean to imply that there cannot be expressions of tachymetabolism and bradymetabolism within a single clade. Apparent reversions from endothermy to ectothermy in crocodylians (Seymour *et al*., 2004), phytosaurs (Legendre *et al*., 2016) and notosuchians (Cubo *et al*., 2020) show that the expression of endothermy may be more opportunistic than usually assumed and may explain the occurrences of ectothermy in living reptiles (Section VI.2*b*). This pattern of occurrences of both endothermy and ectothermy within closely related amniotic taxa through deep time has been described by Cubo & Jalil (2019, p. 327) as a “flickering on and off” of bradymetabolism and tachymetabolism. Other examples can be found in Botha‐Brink & Smith (2011), Legendre, Segalen & Cubo (2013) and Appendix S1.

We considered evidence in the following six categories as proxies for endothermy and we sought multiple lines of evidence wherever possible.

### (1) Evidence from osteohistology

The microstructure of bone commonly survives fossilisation, thereby recording information about an individual's ontogenetic rate of growth, chronological age and age at adult size, attributes that relate to the metabolic regime under which growth occurred (Padian & Lamm, 2013). High rates of growth are associated with endothermy and also with the occurrence of fibrolamellar bone (FLB), which is frequently found in today's endothermic mammals and birds, but rarely in reptiles. Enlow & Brown (1957) were the first to note the similarity of dinosaur bone structure to that of mammals, writing (p. 200) “In structure, the bone of these extinct animals [dinosaurs] is similar to, if not identical with, the bone tissues of many living mammals, including man.” Currey (1962) examined the histology of prosauropod bone and commented on its high vascularisation and structure being more like a mammal or bird than a reptile, suggesting “physiological specialization” (p. 238). Bakker (1972) and de Ricqlès (1974) were early acceptors of its relevance as an indicator of high metabolic rate, and the presence of well‐vascularised FLB, notably in the long bones, became used commonly for inferring probable endothermy in extinct amniotes. Under polarised light, the fibrous component of FLB has a ‘woven’ appearance, due to the rapid rate of bone mineral deposition. Studies on extant vertebrates reveal that woven‐fibred bone forms at apposition rates ranging from 5 to 171 μm per day (Castanet *et al*., 1996, 2000; de Margerie, 2002; de Margerie, Cubo & Castanet, 2002; Starck & Chinsamy, 2002; de Margerie *et al*., 2004). At an apposition rate of 5 μm or less per day, bone fibres are deposited slowly into well‐organised parallel‐fibred or lamellar arrangements (Castanet *et al*., 1996). Therefore, the presence of FLB in extinct vertebrates implies a high bone apposition rate, and elevated growth rates sustained during ontogeny can indicate endothermic metabolism (Farlow, Dodson & Chinsamy, 1995). Numerous recent studies have relied on the use of FLB as an indicator of high bone growth rate and, in turn, tachymetabolism (e.g. Padian, de Ricqlès & Horner, 2001; Montes *et al*., 2007; de Ricqlès *et al*., 2008; Cubo *et al*., 2012; Houssaye, 2013; Legendre *et al*., 2013; Padian & Lamm, 2013; Stein & Prondvai, 2014; Klein, Foth & Schoch, 2017). The timing of our survey is fortunate because it follows the emergence of improved quantitative techniques for analysing osteohistological attributes that allow inferences about the metabolic status of fossil samples by comparing them with equivalent attributes of extant ectotherms and endotherms whose metabolic rates are known (Legendre *et al*., 2016; Olivier *et al*., 2017; Fleischle, Wintrich & Sander, 2018; Cubo & Jalil, 2019). Nevertheless, Padian & de Ricqlès (2020) urged against relying on single samples when inferring endothermy and urged in favour of basing diagnosis on evidence of high growth rate sustained through much of ontogeny. We sought such evidence in our literature survey.

Reports of FLB in the bones of turtles and juvenile crocodylians (Enlow, 1969; Reid, 1997; Tumarkin‐Deratzian, 2007; Woodward, Horner & Farlow, 2014; Company & Pereda‐Suberbiola, 2017) have led to questions about its utility for inferring endothermic metabolism. However, when present in extant terrestrial ectotherms, FLB is isolated within a cortex comprised primarily of slowly formed parallel‐fibred or lamellar bone, likely coinciding with ephemeral optimal environmental conditions permitting a brief period of rapid growth (Woodward *et al*., 2014). It occurs only in juvenile crocodylians and is lost as they grow, providing an example of what Padian & de Ricqlès (2020) refer to as anomalously high growth rates that may be seen in very young ectotherms. Alternatively, FLB in juvenile crocodylians may be an atavistic property pointing to an endothermic ancestry (Seymour *et al*., 2004). It remains the case that the only extant terrestrial vertebrates capable of sustained FLB formation (i.e. sustained high growth rates) throughout ontogeny are endothermic.

In our literature survey, osteohistological information was the most common category of data available, but only in eight out of nearly 50 taxa were there no other proxies available. One of these (phytosaurs) we judged as most likely ectothermic, whereas the archosauromorph *Aenigmastropheus* we judged as most likely endothermic but left it uncertain. In the remaining six the osteological evidence in support of tachymetabolic endothermy is strong by itself (full details are provided in Appendix S1).

### (2) Evidence from central cardiovascular physiology

**Fig. 2:**
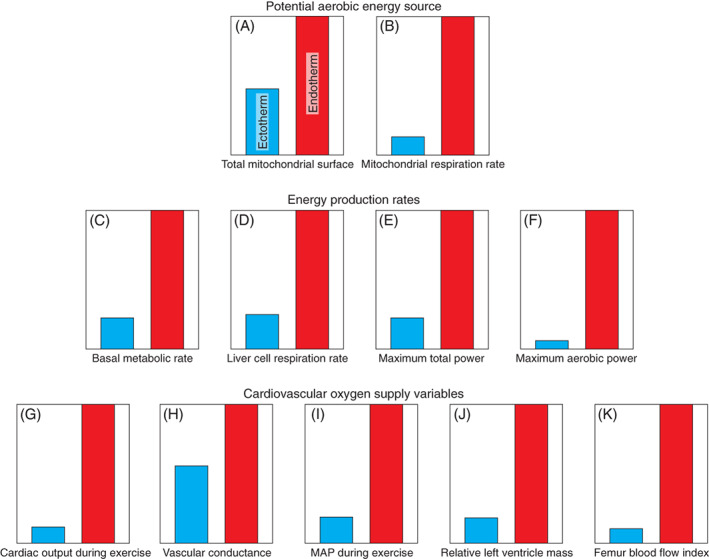
Differences in energy metabolism and cardiovascular oxygen supply variables between representative ectotherms (blue) and endotherms (red). All graphs provide values for ectotherms as a proportion of those for endotherms, under similar temperatures and body sizes. The potential aerobic energy source is compared as total mitochondrial surface area (A) and maximum mitochondrial respiration rate (B). Rates of energy production include basal metabolic rate (C), an example of *in vitro* cellular respiration rate (D), maximum total power output (aerobic and anaerobic) during intense exercise (E) and maximum aerobic power output (F). On the bottom row are cardiovascular variables associated with delivery of oxygen for aerobic metabolism. Cardiac output during exercise (G) is similar to maximum aerobic power output (F). Vascular conductance (H) is the inverse of vascular resistance and suggests that blood flows more easily through the endotherm's circulation, however, the means are not significantly different. Mean systemic arterial blood pressure (MAP) during exercise is higher in endotherms (I), in direct association with higher cardiac output. The greater work of the endotherm heart is represented by greater ventricle mass (J). Finally, greater aerobic exercise levels of endotherms are matched by greater blood flow rates to the femur shaft through the nutrient foramen (K), presumably associated with repair of exercise‐induced microfractures (secondary osteon formation). The data are based on published information. Total mitochondrial inner surface area (A) is the sum of areas in all major tissues (liver, kidney, brain, heart, lung and skeletal muscle) and values for 1 kg animals are calculated from allometric equations (Else & Hulbert, 1985). Respiration rates of isolated mitochondria (B) are from similarly sized lizards and mice with succinate substrate at 37°C (Berner, 1999). Basal metabolic rates (C) are based on averages of 1 kg fish, amphibians and reptiles as ectotherms and mammals and birds as endotherms, all adjusted to 38°C (White, Phillips & Seymour, 2006). Liver cell respiration rates per mg of protein (D) are from rats and lizards at 37°C (Hulbert & Else, 2004). Maximum total power output (E) and maximum aerobic power output (F) are based on 1 kg crocodiles compared to mammals in general (Seymour, 2013). Cardiac output (G), systemic vascular conductance (H), mean systemic arterial blood pressure (MAP) (I) and relative left ventricle mass (J) are calculated for 1 kg animals from allometric equations (Hillman & Hedrick, 2015). Femur blood flow indices (K) in 1 kg mammals and non‐varanid reptiles are calculated from allometric equations (Seymour *et al*., 2012).

The oxygen that supports the metabolic rate is delivered by the cardiovascular system, so the anatomy and physiology of the heart and arteries provide perhaps the most direct indication of metabolic status, short of measuring metabolic rate itself. Although the soft anatomy and physiology is lost in extinct species, a large fossil skeleton can indicate that the once‐living animal was an endotherm, because the vertical distance between the normal position of the vertebrate heart to the top of the body can demonstrate that the animal had an elevated arterial blood pressure. To appreciate how a skeletal distance is related to blood pressure and metabolic rate, it is useful to compare ectotherms and endotherms in terms of quantitative differences in anatomy and physiology of the oxygen cascade (Fig. 2).

Metabolic and cardiovascular variables in living ectotherms and endotherms differ greatly and in parallel at all levels, from the mitochondria, through the oxygen transport systems to the aerobic behaviour of the entire animal. The potential aerobic energy production rate by the mitochondria is much higher in endotherms (Fig. 2A, B). Basal metabolic rates are about fivefold higher, and maximal aerobic metabolic rates about 17‐fold higher in extant endotherms than in ectotherms (Fig. 2C, F). This is met by a cardiac output sixfold higher at rest and eightfold higher during exercise (Fig. 2G). The high cardiac outputs of endotherms are sent to systemic tissues that have slightly higher (but not significantly so) vascular conductance than ectotherms (Fig. 2H). The inverse of conductance is resistance, which is therefore a little lower in endotherms, but not low enough to compensate for the high blood flow rate. This is possibly due to a mismatch between a greater number of blood vessels in tissues (which reduces resistance) and smaller capillaries (which increase resistance) of endotherms compared to ectotherms (Huttenlocker & Farmer, 2017). The result is that the mean systemic arterial blood pressure (MAP) is 3.5‐fold higher in resting endotherms than in ectotherms (Seymour *et al*., 2004), and 5.5‐fold higher in exercising endotherms than in ectotherms (Fig. 2I). In common units (1 mm Hg = 133.3 Pa), MAP averages about 100 mm Hg in resting mammals and 135 mm Hg in birds, compared to 35 mm Hg in extant reptiles, and individual values for endothermic and ectothermic species of a similar body size scarcely overlap (Seymour, 2016). Thus, a high MAP is functionally related to high aerobic tachymetabolism, because it is associated with high rates of oxygen delivery by the blood. The greater cardiac output and a higher MAP combine to increase heart work, which accounts for a 5.5‐fold larger left ventricle mass in endotherms (Fig. 2J). These cardiovascular variables are all correlated functionally with the high oxygen demands of tachymetabolic endothermy. Correlations often do not imply causation, but all of these correlations certainly do.

**Fig. 3:**
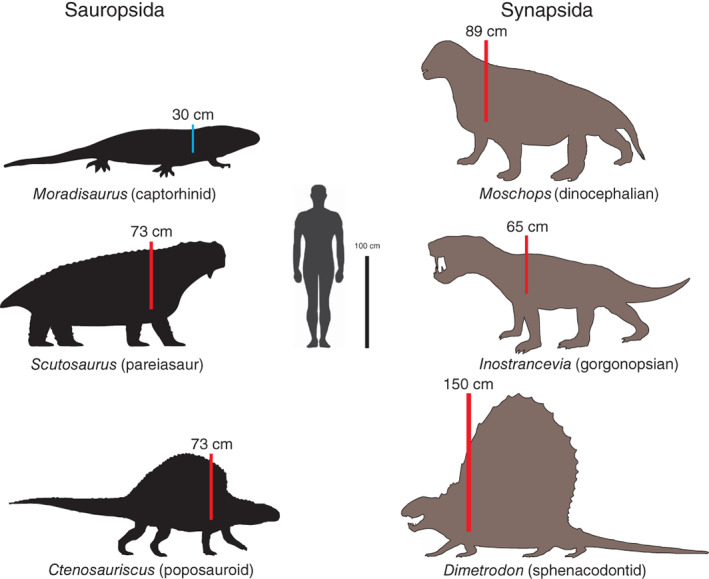
Relative sizes of some of the largest known members of Permian and Triassic sauropsids and synapsids, compared to a human and a 1 m scale. The vertical distances from the estimated heart position to the top of the body (H–H) are indicated. All of these animals, except for the captorhinid (*Moradisaurus*), must have had mean systemic arterial blood pressures (MAPs) higher than the 89 mm Hg (11.87 kPa) required to support a vertical blood column of 50 cm (*P*_*g*_ = 39 mm Hg, 5.20 kPa) with an adequate perfusion pressure against the vascular system's resistance (*P*_*r*_ = 50 mm Hg, 6.67 kPa). The total pressures are well within the range exhibited by extant tachymetabolic endotherms. Conversely, the largest captorhinid needed to support only a short vertical column of blood above the heart. It is important to realise, however, that a short H–H distance cannot be used as evidence of bradymetabolic ectothermy; small tachymetabolic endotherms typically have a high MAP but low H–H values.

Of course, most of the metabolic and cardiovascular variables in Fig. 2 cannot be measured directly in extinct species, however we can obtain an indication of MAP from reconstructions of fossil skeletons (Seymour, 1976, 2016). There are two components of MAP that are relevant in demonstrating endothermy. First is the systemic arterial perfusion pressure (*P*_*r*_), which is the pressure necessary to push the blood through the inherent resistance of the circulatory system. The second component of MAP is the necessity to support the vertical blood column between the level of the heart and the top of the body. This gravitational component of blood pressure (*P*_*g*_) is the product of the density of blood (ρ), the acceleration due to gravity (*g*) and the vertical height (*h*) (*P*_*g*_ = ρ*gh =* 77 mm Hg for every 100 cm vertical distance above the heart). Because ρ and *g* are nearly constant, *P*_*g*_ is proportional to height of the body above the heart. The heart is located at the lowest part of the body cavity near the pectoral girdle and the head is often at the top of the body, so we have historically called this vertical component above the heart the ‘heart‐to‐head (H–H)’ distance, although herein we also apply H–H distance to the distance between the heart and the highest part of the body (Fig. 3). MAP in mammals is often, but erroneously considered to be a constant, about 100 mm Hg, independent of body size and shape. However, among 47 mammalian species, MAP significantly increases non‐linearly from about 90 mm Hg in a mouse to about 150 mm Hg in an elephant (White & Seymour, 2014). *P*_*r*_ appears to decrease over this range, from 90 mm Hg in the mouse to 45 mm Hg in the elephant, while the gravitational component of MAP (*P*_*g*_) increases from almost zero in the mouse to 105 mm Hg in the elephant. MAP is about 200 mm Hg in a standing adult giraffe, of which *P*_*g*_ accounts for 155 mm Hg and *P*_*r*_ for 45 mm Hg. Among 16 mammalian species, MAP increases significantly with H–H distance, with or without inclusion of the giraffe (Sandal, Damgaard & Secher, 2020). It is thus possible to estimate *P*_*g*_ from the vertical distance from the heart level to the top of the body.

No vertebrate animal can have a vertical H–H distance greater than can be supported by their MAP at heart level, because the siphon principle and its negative arterial vascular pressures in the head would cause vascular collapse, cessation of blood flow and other fatal problems (for references, see Seymour, 2016). Extant bradymetabolic reptiles have a mean MAP of 35 mm Hg and are therefore limited in vertical size. Because MAP = *P*_*g*_ + *P*_*r*_, a MAP of 35 mm Hg would equal *P*_*g*_ when the H–H distance reaches 45 cm and *P*_*r*_ becomes zero. To maintain a positive *P*_*r*_ in the head and permit blood flow there, a maximum H–H distance of about 35 cm is reasonable for an ectotherm. Extant endotherms, on the other hand, have a high MAP to support their higher metabolic intensity, so they can also support a taller posture. For example, a MAP of 90 mm Hg at heart level in the average mammal could support a H–H distance up to 58 cm and still have sufficient perfusion pressure (*P*_*r*_ = 45 mm Hg) to maintain flow through the brain.

The previous paragraph indicates that MAP in extant reptiles can support a maximum H–H distance of 35 cm and MAP in extant mammals can support a minimum H–H distance of 58 cm. Therefore, extinct species with skeletons indicating H–H distances over about 50 cm were probably endotherms (Fig. 3). It is important to note, however, that whereas a high H–H distance implies tachymetabolic endothermy, a low H–H distance does not necessarily imply ectothermy, as the tachymetabolism of so many extant small mammals and birds makes clear. Endothermy in primitive small species would allow the evolution of larger, erect species with larger distances between the heart and the top of the body. Examples of tall, presumably endothermic, sauropsids and synapsids begin to occur during the Permian and Triassic (Fig. 3). Basal archosaurs also include species with high H–H distance (e.g. *Protorosaurus*, *Smok*, *Ornithosuchus*) and later Mesozoic archosaurs (e.g. *Tyrannosaurus*, *Shantungosaurus*) exhibited H–H distances equivalent to giraffes. Interestingly, from bone histology, the prostrate, crocodylian‐shaped Triassic Phytosauria (Legendre *et al*., 2016) and Cretaceous Notosuchia (Cubo *et al*., 2020) appear to be ectotherms secondarily, and all have short vertical distances above the heart level.

High MAP is related to a requirement for complete separation of systemic and pulmonary circuits in a typically endothermic, four‐chambered heart. If the high MAP were applied also to the pulmonary circuit, the lungs would be at risk from filtration of fluid into pulmonary air spaces. To avoid that, pulmonary blood pressures in all reptiles, birds and mammals are low: 15–40 mm Hg (Johansen, 1972; Hicks, 1998). The occurrence of four‐chambered hearts in crocodiles was once thought to diminish the value of these hearts as evidence for endothermy, but their hearts are now proposed, based on several lines of evidence, to express a reversal to ectothermy from their endothermic basal archosaurian ancestry (Seymour *et al*., 2004), a conclusion supported also by osteological evidence (Cubo & Jalil, 2019).

The functional correlation between erect posture, high H–H distances and endothermy in dinosaurs was first pointed out 45 years ago (Seymour, 1976). Since its original acceptance by some palaeontologists (e.g. Ostrom, 1980), there have been no published criticisms of the cardiovascular principles involved with inferring endothermy from high H–H distance. Seymour (1976) also argued that long‐necked sauropod dinosaurs would have required MAPs exceeding 500 mm Hg if they held the neck vertically and that this was unlikely. This was a separate issue, unrelated to endothermy. There have been attempts to solve the sauropod's blood pressure problems with multiple hearts and vascular siphons. A summary of the controversy shows that the only solution is that they could not have lifted their heads high (Seymour, 2016). The sauropod neck controversy may have been partly responsible for the absence of MAP evidence being presented routinely in discussions about dinosaur tachymetabolic endothermy.

### (3) Evidence from long bone foramina; femur blood flow index

Endothermy is supported by a much higher consumption of oxygen and a much greater blood flow than is required by ectothermy (Fig. 2) and another source of information about an extinct amniote's metabolic intensity is provided by estimating the size of foramina in fossil long bones through which blood vessels passed. This evidence is a corollary to high bone vascularity observed with palaeohistology. Bones receive blood to grow (modelling), to repair microfractures (remodelling), to exchange materials and to support haematopoiesis in the bone marrow. In most species, blood enters the shaft of the femur through the principal nutrient foramen, thence perfusing the endosteal cavity and bone marrow before exiting *via* numerous vessels in the cortical bone (Marenzana & Arnett, 2013). The size of the foramen can provide an index which is related to the rate of blood flow to the bone shaft (Hu, Nelson & Seymour, 2020, 2021a,b) and flow rate is about 10 times higher in extant mammals and birds than in extant non‐varanid reptiles (Fig. 2K), and even higher if differences in blood pressure are considered (Seymour *et al*., 2012; Allan *et al*., 2014). These large differences in femoral blood perfusion between living ectothermic and endothermic amniotes can be used to evaluate the size of nutrient foramina found on fossil long bones as records of their metabolic intensity. Data gained from numerous early sauropsids and synapsids indicate the level of aerobic metabolic rate during daily locomotion. In particular, the calculated femoral blood flow indices from a range of Triassic, non‐archosaurian archosauromorphs and pseudosuchians are not significantly different from those of extant endothermic mammals and are larger than those from extant non‐varanid reptiles (Seymour *et al*., 2012, 2019). Likewise, nutrient foramina from non‐mammalian synapsids from the Carboniferous to early Late Triassic, including caseids, edaphosaurids, sphenacodontids, dicynodonts, gorgonopsids, and cynodonts are also in the range of recent mammals and, interestingly, varanid lizards (Knaus *et al*., 2021). Varanid lizards need special mention because they differ physiologically from other extant squamates in a number of unique ways. Despite being bradymetabolic they have aerobic metabolic rates that approach those of mammals during locomotion, bone remodelling in response to exercise and prominent bone cortical vascular canals, all general characteristics of endotherms (see references in Seymour *et al*., 2012). Intriguingly, they also have multicameral lungs with unidirectional patterns of airflow (Schachner *et al*., 2014; Cieri & Farmer, 2016), a characteristic of avian and crocodylian lungs. Nevertheless, they do not thermoregulate physiologically, lack insulation and are classified as ectotherms (Seebacher & Grigg, 2001). In our survey we made reference to calculations based on the size of the nutrient foramen as indicating an elevated metabolic intensity in 15 taxa. In all of them it was in combination with at least one other proxy, and in 11 of them it was in combination with 2, 3 or 4 other independent proxies, as described in Appendix S1.

### (4) Evidence from the biomechanics of bipedality

A biomechanical study estimated the metabolic rates of 14 extinct bipedal dinosauriforms during walking and running, based on well‐validated data from extant species (Pontzer, Allen & Hutchinson, 2009). It showed that walking and slow running by the larger extinct dinosaurs could not have been supported by the maximum aerobic capabilities of modern ectotherms and fell within the capability of extant birds and mammals. In practical terms, the implication is that any bipedal sauropsid or synapsid >10 kg would be unable to move swiftly unless it was tachymetabolic, and any biped >200 kg would need to be tachymetabolic even to maintain walking pace. The high energy cost of bipedality is demonstrated by the fact that there are no obligatory bipedal ectotherms living today.

### (5) Evidence from palaeothermometry

The application of palaeothermometry to fossil amniotes usually involves the analysis of stable oxygen isotope or carbon and oxygen isotope ratios in fossilised teeth or other bone (Barrick, Showers & Fischer, 1996; Fricke & Rogers, 2000; Amiot *et al*., 2006; Bernard *et al*., 2010; Eagle *et al*., 2011; Harrell, Perez‐Huerta & Suarez, 2016; Rey *et al*., 2017). Teeth are often chosen because of their robustness to change, and it makes physiological sense too, because teeth are well perfused with blood at core *T*_b_ during development. The method is controversial because of uncertainty about how representative the calculated temperatures are due to diagenetic effects (Trueman *et al*., 2003), so we have interpreted such data with caution. Recently a more accurate method has been applied, for example by Dawson *et al*. (2020) who reported endotherm‐like *T*_b_ from eggshells in three clades of dinosaur: a hadrosaur (Ornithischia) and two Saurischia, a theropod (*Troodon*) and another, possibly a sauropod. The method is useful in biology because it measures the thermodynamic preference of two heavy, rare isotopes such as ^13^C and ^18^O to bind together. This process is temperature dependent and is therefore a proxy for the temperature at which carbonate minerals were laid down (Affek, 2012).

Caution is needed particularly when interpreting palaeothermometric values implying warm and stable *T*_b_ in very large animals, because it could possibly be explained equally well by either endothermy or gigantothermy. Gigantothermy, sometimes called passive homeothermy and usually thought of as being more relevant to ectotherms, is a phenomenon that arises because, as animals grow, their surface area to mass ratio decreases and their thermal inertia increases until in very large individuals thermostability is approached. As an illustration, in a tropical winter field study of *Crocodylus porosus*, Grigg *et al*. (1998) and Seebacher, Grigg & Beard (1999) found that whereas *T*_b_ in a 32 kg individual cycled daily by about 6°C, a 1000 kg animal's *T*_b_ cycled by about 2°C and calculations showed that a 10000 kg individual would be stable within about 0.1°C when *T*_a_ ranged daily by 20°C. Also, because rates of heating and cooling are typically asymmetrical, it is possible for a very large ectotherm to become essentially thermostable at a *T*_b_ warmer than *T*_a_. So endotherm‐like palaeothermometric *T*_b_ data need to be treated with caution, and resolving whether tachymetabolism or gigantothermy provides an explanation is not necessarily straightforward. Brice & Grigg (in press) recently used a modelling approach to explore the possibility that very large wholly aquatic sauropsids with metabolism characteristic of ectotherms could develop endotherm‐like *T*_b_ by gigantothermy (see Section V.1*b*) and Appendix S1). In short, the modelling showed that gigantothermy could explain observations of endotherm‐like *T*_b_ only in comparatively warm water, and only in the largest of the really enormous animals. The observed *T*_b_ data thus could be explained only by tachymetabolic endothermy. The conclusions of Brice & Grigg (in press) also draw attention to misunderstandings about the size threshold for terrestrial gigantothermy, and about overly optimistic assumptions about the capacity of terrestrial gigantothermy to confer the warmth, long‐term thermal stability and energy levels typical of today's birds and mammals in any extinct sauropsid or synapsid. As recognised by Reid (1997) and shown quantitatively by Seymour (2013), warmth gained by gigantothermy produces a warm ectotherm, not a tachymetabolic endotherm.

### (6) Evidence from respiratory turbinates

The presence of nasal respiratory turbinate bones (RTs) in both mammals and birds has long been advocated as diagnostic of endothermy (Hillenius, 1992, 1994; Ruben, 1995; Ruben *et al*., 2012). In mammals, RTs function in the conservation of heat and water (Schmidt‐Nielsen, Hainsworth & Murrish, 1970), and their absence has been used to argue against the occurrence of endothermy in dinosaurs (Chinsamy‐Turan & Hillenius, 2004; Hillenius & Ruben, 2004; Ruben *et al*., 2012). But does their absence necessarily dictate ectothermy? Incongruities that challenge the assumption that RTs are essential for endothermy suggest that they are not a strict requirement, especially in equable environments with ample water (Seymour, 2004; Seymour *et al*., 2004). Moreover, a comparison of respiratory pathway surface areas in relation to metabolic rate between mammals and birds suggests that RTs are not required for endothermy in birds (Owerkowicz *et al*., 2015) – a conclusion with relevance for dinosaurs. In short, while the presence of RTs can be accepted as indicative of endothermy, their absence is not indicative of ectothermy.

## V RESULTS: WHOLE‐BODY ENDOTHERMY APPEARS EARLY AND IS WIDESPREAD IN BOTH SAUROPSIDA AND SYNAPSIDA

In a recent review, Benton (2020) downplayed the significance of earlier occurrences of endothermy and concluded that whole‐body endothermy evolved contemporaneously in Archosauromorpha (Sauropsida) and Therapsida (Synapsida) early in the Triassic, after the Permian–Triassic Mass Extinction (PTME) (see Section V.2*b*). By contrast, guided by the six proxies discussed in Section IV, we found compelling evidence for widespread occurrences of tachymetabolic endothermy well before the PTME among both Sauropsida and Synapsida, consistent with our prediction.

**Fig. 4:**
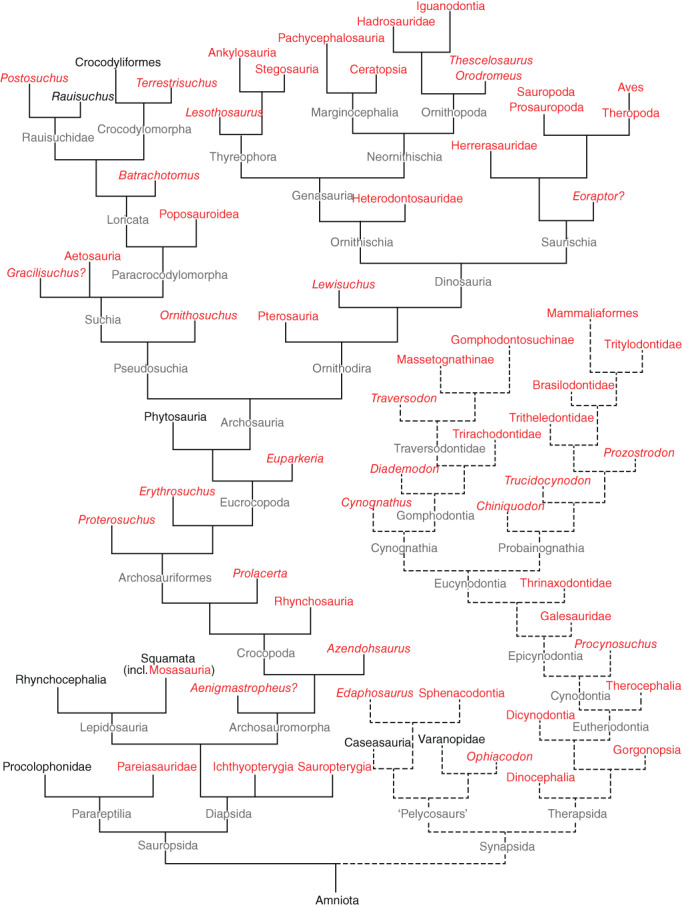
Occurrences of tachymetabolic endothermy in amniote taxa, shown in red, as judged on the basis of criteria discussed in Section IV. Those for which the evidence is inconclusive are shown with a question mark. For taxa shown in black we found either no evidence for endothermy or no relevant information. The figure is indicative rather than comprehensive. The evidence on which the diagnosis for each taxon or taxonomic group is based is provided in Appendix S1. The identification of a clade as having tachymetabolic endothermy is not meant to imply necessarily that that tachymetabolism is characteristic of the whole group. We counted a strong indication of endothermy in one genus, for example, as an occurrence in that taxonomic group, even if an apparently ectothermic genus was also found. Assuming the proxies we used are valid, the results are congruent with our hypothesis that the capacity to express whole‐body endothermy arose very early in amniotes and that this capacity, seen in extant birds and mammals, is plesiomorphic. The results are also consistent with recent findings that all birds and many mammals possess similar biochemical machinery driving skeletal muscle NST, proposed as the ancient and still current source for NST in many, perhaps most extant mammals and all birds (supplemented by BAT in some eutherian mammals). Schematic phylogeny from multiple sources. Artwork by David Kirshner.

Because we are primarily interested in how early whole‐body endothermy emerged, we focus in detail here in the main text on the ‘Parareptiles’ (Sauropsida), ‘Pelycosaurs’ and the pre‐PTME Therapsida (Synapsida). We also include brief summaries of our findings for the aquatic Mesozoic diapsids, Ichthyosauria, Sauropterygia and Mosasauria, some of which grew to enormous size, and Archosauromorphs and Archosaurs. The results are summarised in Fig. 4, and full details of the survey results can be found in Appendix S1.

### (1) Endothermy among sauropsids

#### (a) Procolophonidae and Pareiasauridae (‘Parareptilia’)

These two groups are particularly relevant to our question about how early the first whole‐body endotherms appeared. They have been subject to comparatively few relevant studies, but we found one for Procolophonidae and several for Pareiasauridae.

##### (i) Procolophonidae (Late Permian–Late Triassic)

Small (30–50 cm) lizard‐like ‘parareptiles’. Botha‐Brink & Smith (2012) studied the limb bone histology of three small Triassic procolophonid parareptiles, *Sauropareion anoplus* (Early Triassic), *Procolophon trigoniceps* (Late Permian–Early Triassic) and *Teratophon spinigenis* (Middle Triassic), from the Karoo Basin of South Africa, in order to infer their palaeobiology. No FLB was observed, but they noted the bone histology was different from typical reptile bone, and well vascularised in a couple of genera, to the extent that suggested rapid growth early in development. They considered the morphology and bone histology to suggest a burrowing lifestyle. From the available information, no conclusion can be reached about their metabolic status.

##### (ii) Pareiasauridae (Middle–Late Permian)

**Fig. 5:**
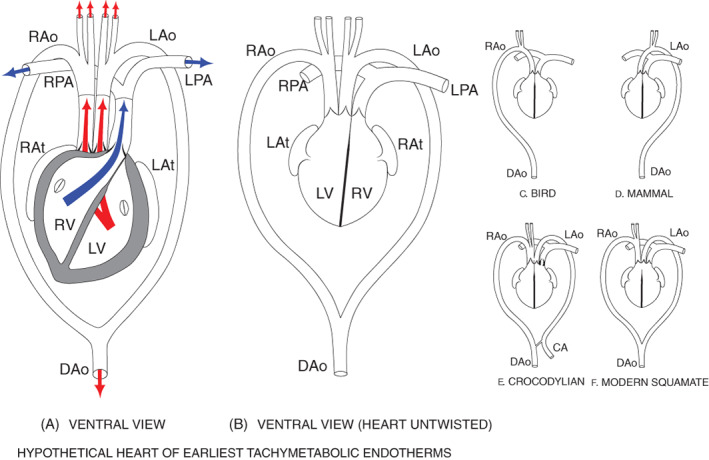
The hypothetical structure of the heart and outflow tracts of the earliest whole‐body (tachymetabolic) endothermic amniotes, shown schematically with the heart twisted as normal in relation to its outflow tracts (A) and untwisted in (B) to allow easier comparison with the thumbnail sketches (right) of four extant amniotes. All are represented as if viewed from below. Interventricular septa are drawn to represent their muscular and membranous sections. For the crocodylian (E) note the unusual location of the left aorta (LAo) exiting the right ventricle (RV) next to the exit leading to the pulmonary arteries (LPA and RPA), the foramen of Panizza depicted as a small gap in the common wall between the right and left aortae (RAo and LAo), and the cog‐tooth valve which contracts to initiate pulmonary bypass shunting. CA, coeliac artery; DAo, dorsal aorta; LAt, left atrium; LV, left ventricle; RAt, right atrium. Artwork by David Kirshner.

Many were large (1–3 m long, 100–600 kg) stocky herbivores with semi‐erect to upright stance. They have been reconstructed as chunky‐looking herbivores, somewhat resembling domestic cattle. Canoville & Chinsamy (2017) examined samples from the long bones and ribs of several South African pareiasaurs, including *Pareieasaurus*, *Pareiasuchus*, *Bradysaurus* and *Anthodon* (all Late Permian) and found a generally similar microstructure among them. They found no FLB, but extensive well‐vascularised Haversian systems with primary and secondary osteons. They concluded that the bone histology indicated relatively rapid growth early in ontogeny, with periosteal growth slowing later and growth continuing for several years during adulthood. They suggested that the early high growth “could possibly be used in support of previous interpretations by de Ricqlès (1978a) that pareiasaurs may have had intermediate physiologies as compared to other basal amniotes, with a tendency towards endothermy” (p. 1063). Looy *et al*. (2016) studied the osteohistology of limb bones and a scapula of *Bunostegos* (Late Permian) from Niger. They interpreted the results as evidence of fast bone deposition and elevated metabolism, in accordance with previous studies on pareiasaurs (de Ricqlès, 1978a,b; Canoville & Chinsamy‐Turan, 2011). These interpretations are congruent with the semi‐erect to upright limb posture of pareiasaurs (Bakker, 1971; Sumida & Modesto, 2001; Turner *et al*., 2015). After histological examination of bone from two Upper Permian pareiasaurs from Russia, *Deltavjatia rossica* and *Scutosaurus karpinskii*, Boitsova *et al*. (2019) reported relatively short periods of rapid growth early in life, with well‐vascularised, fast‐growing FLB separated by lines of arrested growth (LAGs). Although this rapid growth window was relatively short, it apparently accounted for about 50% (*Deltavjatia*) or 75% (*Scutosaurus*) of their growth to maximum size. A transition to poorly vascularised parallel‐fibred and lamellar bone separated by LAGs followed, suggesting that slower but still periodic growth continued for several years into adulthood. By contrast, much slower growth was inferred in the Middle Permian *Provelosaurus americanus* from southern Brazil (Farias, Schultz & Soares, 2019). Following the logic of Seymour (1976, 2016), the body size and habitus of the larger pareiasaurs such as *Pareiasaurus*, *Scutosaurus* and *Bunostegos* imply a H–H distance requiring endotherm‐equivalent mean arterial pressure (MAP) (Fig. 3) and a four‐chambered heart (Fig. 5). Moreover, Rey *et al*. (2020) reported stable oxygen isotope values from phosphates in teeth and bone from *Bradysaurus* and some unidentified pareiasaurs. Used as a proxy for water dependence, the values clustered with those from coeval tachymetabolic endothermic synapsids. Collectively, satisfying Proxies 1, 2, and 5, there is strong evidence for the occurrence of tachymetabolic endothermy in Pareiasauridae.

#### (b) Ichthyosauria, Sauropterygia and Mosasauria

Although these are not among the earliest sauropsids, we paid considerable attention to three groups of extinct aquatic Mesozoic non‐archosauromorph diapsids because they represent a special case and their metabolic status has been in some doubt: Ichthyosauria (Early–Late Mesozoic), Sauropterygia (plesiosaurs and pliosaurs) (Late Triassic–Late Cretaceous) and Mosasauria (Squamata) (Cretaceous) (Fig. 4). We summarise our results here, for full details see Appendix S1. ‘Some form of endothermy’ or just ‘endothermy’ have been suggested for each group, based on palaeothermometry of teeth and bone that revealed endotherm‐like *T*_b_ in the mid‐30s °C (Bernard *et al*., 2010; Harrell *et al*., 2016). However, because of the very large body size of some genera in each group when adult, up to 7 m and even 12 m in mosasaurs, gigantothermy was potentially a possible alternative explanation. Brice & Grigg (in press) modelled a range of thermal and metabolic scenarios in which the reported values for *T*_b_ in these aquatic sauropsids could be achieved. The modelling showed that if they were bradymetabolic ectotherms, even in relatively warm water (28°C) they would have to grow to at least 10 m (≈ 4600 kg) before gigantothermy would allow them to be homeothermic at 34°C without either additional insulation or activity. And even with insulation and activity, none could have maintained the reported *T*_b_ in the mid‐30s °C across the likely range of water temperatures (16–29°C), even as adults. The implication is that the bird‐ and mammal‐like body temperatures reported for these aquatic sauropsids are best explained by tachymetabolic endothermy. This conclusion is supported by multiple independent osteohistological studies which inferred high rates of growth and a high metabolic rate in representatives of each group. Interestingly, in an osteohistological study of placodonts (mid‐Triassic), Fleischle *et al*. (2018, p. e4955) suggested that an elevated metabolic rate and growth rate may have evolved at the base of Sauropterygia “or may have even been inherited from terrestrial progenitors”. For each of the three clades there is good evidence for tachymetabolic endothermy (Proxies 1, 5, backed up by analyses precluding gigantothermy).

#### (c) Archosauromorpha and Archosauria

Fifty years ago, the suggestion that dinosaurs may have been ‘warm blooded’ was controversial and the ideas of its most vocal protagonist, Robert Bakker, were viewed with scepticism. It has taken a long time, but with the development of many novel techniques and with new evidence the last couple of decades have seen a vast change in perceptions about dinosaur metabolic physiology, and in the Archosauromorpha and Archosauria as a whole. Birds are now accepted as theropod dinosaurs and although the idea itself was much older, Ostrom (1973) detailed the first formal and well‐argued proposal for it. Ostrom's paper facilitated acceptance by many that at least some of the non‐avian dinosaurs must have been endothermic. Although still not accepted universally (e.g. Rezende *et al*., 2020) the emerging consensus is that endothermy was common in Archosauria and basal in Archosauromorpha in the Late Permian–Early Triassic (e.g. Seymour *et al*., 2004; Ezcurra, Scheyer & Butler, 2014; Ezcurra, 2016; Legendre *et al*., 2016; Cubo & Jalil, 2019; Benton, 2020; Cubo *et al*., 2020). Our literature survey reinforces this consensus. Using the proxies discussed in Section IV, we found good evidence for occurrences of endothermy in many groups of both Pseudosuchia and Ornithodira, including Pterosauria and essentially all the well‐known groups of Dinosauria (Fig. 4), with full details provided in Appendix S1.

### (2) Endothermy among early synapsids

Like the sauropsids, the synapsids also comprise two distinct groups early on, the ‘Pelycosaurs’ (from Late Carboniferous) and the Therapsids (from Early Permian).

#### (a) ‘Pelycosaurs’

The informal, non‐cladistic name ‘Pelycosaur’ is often used to refer to five categories of so‐called ‘mammal‐like reptiles’ comprising ‘all synapsids not held to be within the clade Therapsida’, that is, the Caseasauria, Varanopidae, Ophiacodontidae, Edaphosauridae and Sphenacodontia. Each arose in the Late Carboniferous, and none survived past the mid‐Permian, unless the Therapsida arose from the Sphenacodontia as suggested by Benson (2012). Until the last decade or so most consideration of the thermal biology of pelycosaurs assumed they were ectothermic and focused on a possible role for the dorsal ‘sails’ of *Dimetrodon* (Sphenacodontia) and *Edaphosaurus* (Edaphosauria) as heat‐exchange organs. There have been osteohistological studies but few with possible endothermy as a focus. In the Caseasauria and Varanopidae, reviewed in Appendix S1, osteohistological data revealed no evidence relevant to endothermy. Also, it should be noted that Macdougall *et al*. (2018) questioned whether varanopids are correctly placed within Synapsida, and Ford & Benson (2020) found them to be a better fit within Diapsida. We will focus our attention on the remaining three groups.

##### (i) Ophiacodontidae (Late Carboniferous–Early Permian)

Shelton & Sander (2017) examined post‐cranial skeletal material from several species of *Ophiacodon*, large (1.6–3 m, 26–230 kg) quadrupedal basal synapsids. The authors described classic characteristics of ‘true’ FLB with the cortex comprising “primary osteons in a woven bone matrix (that) remains highly vascularized throughout ontogeny, providing evidence for fast skeletal growth” (p. 397). This evidence of well‐vascularised FLB sustained throughout ontogeny is compelling. They concluded (p. 397) that “*Ophiacodon* is more advanced or ‘mammal‐like’ in terms of the osteonal development, bone matrix, and skeletal growth than what has been described thus far for any other pelycosaur taxon”. This is in agreement with the much earlier findings of Enlow & Brown (1957) whose osteohistological work convinced them that mammalian endothermy arose in the Carboniferous. In a subsequent osteohistological study, however, on a different ophiacodontid, *Clepsydrops collettii* (Late Carboniferous), Laurin & de Buffrénil (2016) found no FLB and described ‘woven‐like’ bone, implying fast growth early on, then slowing, with similarity to that of varanid lizards. More recently still, using phylogenetic eigenvector mapping to infer the metabolic rate of a range of non‐mammalian synapsids, Faure‐Brac & Cubo (2020) also reported an estimate of metabolic rate for *Clepsydrops* indicative of ectothermy. In the same study, these authors concluded that *Ophiacodon uniformis* also was ectothermic, together with *Dimetrodon*, *Sphenacodon* and an edaphosaur, all of which we judged to be tachymetabolic endotherms on the basis of osteological and cardiovascular evidence [see Section V2*a*(*i*) and (*ii*).]. It is worth noting, however, that among these ‘pelycosaurs’ the metabolic rate Faure‐Brac & Cubo (2020) calculated for *Ophiacodon uniformis* did approach the threshold for endothermy. Additionally, although Knaus *et al*. (2021) did not include their four species of *Ophiacodon* among their list of non‐mammalian synapsids with an elevated maximum metabolic rate (MMR) consistent with endothermy, the MMR calculations for *Ophiacodon* based on eigenvector mapping did cluster well with edaphosaurs, sphenacodontids, dicynodonts and gorgonopsids. On balance, the FLB evidence for tachymetabolic endothermy presented by Shelton & Sander (2017) is strong, and in the studies by both Faure‐Brac & Cubo (2020) and Knaus *et al*. (2021) *Ophiacodon* came very close to meeting the criteria for endothermy. The issue is not resolved but we judge that *Ophiacodon* was most likely characterised by tachymetabolic endothermy (Proxy 1, likely 3 also).

##### (ii) Sphenacodontia (Late Carboniferous–Early Permian)

Using their innovative osteological approach [see Section V.2*a*(*i*)], Faure‐Brac & Cubo (2020) reported estimates of metabolic rate in *Dimetrodon* sp. and *Sphenacodon* sp. as indicative of ectothermy. However, three studies imply that at least some sphenacodontids were endothermic. Huttenlocker, Rega & Sumida (2010) examined the hyperextended spines of *Dimetrodon* and found that, as well as lamellar bone, each exhibited extensive well‐vascularised parallel‐fibred and FLB, implying rapid outgrowth of the spines. Shelton *et al*. (2012) found what they called incipient FLB (IFLB) in the postcranial skeleton of *Dimetrodon natalis* throughout ontogeny. The IFLB comprised highly vascularised woven and parallel‐fibred bone in combination, coupled with incipient primary osteons. They suggested a metabolic rate slightly higher than that in modern reptiles. These studies may be indicative of endothermy, but they are not by themselves convincing. However, Seymour (2016) noted that the top of the neural spine of *Dimetrodon grandis* reached 1.5 m above the heart (Fig. 3), implying a MAP of 115 mm Hg (15.33 kPa), which is well within the endothermic range and requires a four‐chambered heart. More recently it was observed that the nutrient foramina from several species of *Dimetrodon* indicated elevated metabolic rates consistent with endothermy (Knaus *et al*., 2021). Although *Sphenacodon* lacked a tall sail and has been assessed as ectothermic, it seems clear that *Dimetrodon* at least among sphenacodontids was a tachymetabolic endotherm (Proxies 2, 3).

##### (iii) Edaphosauria (Late Carboniferous–Early Permian)

Using phylogenetic eigenvector mapping, Faure‐Brac & Cubo (2020) reported an estimate of metabolic rate in *Edaphosaurus boanerges* that is indicative of ectothermy. Huttenlocker, Mazierski & Reisz (2011) reported FLB in the lateral tubercles of the spines of edaphosaurs but made no interpretation about the animals' metabolic rate. As in *Dimetrodon*, the elevation of the top of the tallest neural spines of *Edaphosaurus* implies endotherm‐like blood pressure (Seymour, 1976, 2016), so large edaphosaurs at least must have been tachymetabolic endotherms. Additionally, nutrient foramina from edaphosaurs indicate elevated metabolic rates consistent with endothermy (Knaus *et al*., 2021). The weight of evidence thus favours tachymetabolic endothermy (Proxies 2, 3).

#### (b) Pre‐Triassic Therapsida

The case for tachymetabolic endothermy in numerous post‐Triassic therapsids is now well established, as the review by Benton (2020) shows, and our literature survey confirms this and expands its taxonomic reach (Fig. 4, Appendix S1). Benton noted its “emergence” in the Late Permian in both sauropsids and therapsids, and “acceleration” in the Early Triassic after the PTME. Noting the loss of so many of the Permian endotherms to the PTME, he thus considered endothermy to have its origins in the Triassic, and contemporaneously in both sauropsids and synapsids. But in sauropsids, as discussed above, whole‐body endothermy is considered basal to Archosauromorpha from the Middle Permian. And among therapsids, whereas the Dinocephalia and Gorgonopsia arose in the mid‐Permian and did not extend to the Triassic, the Dicynodontia, Therocephalia and Cynodontia all spanned the PTME and in each of these there is evidence of endothermy both before and after the extinction event. The results for all therapsids are shown graphically in Fig. 4 and complete explanatory details for all therapsids included in our survey are provided in Appendix S1. Because the significance of pre‐Triassic therapsids may be unappreciated, we review here our findings for the basal, pre‐PTME therapsids Dinocephalia, Gorgonopsia, Dicynodontia, Therocephalia and Cynodontia.

##### (i) Dinocephalia (Middle–Late Permian)

The only evidence we have for this group is that at least one member, *Moschops*, achieved a large stature and a H–H distance of about 89 cm thus requiring a MAP of approximately 120 mm Hg (16.0 kPa) to perfuse the brain, clearly in the endothermic range (Proxy 2) (Fig. 3).

##### (ii) Gorgonopsia (Middle–Late Permian)

Long bone histology of these bear‐sized, quadrupedal carnivores, judged to be active predators on the basis of their striking sabre‐like canines, was first studied by de Ricqlès (1969), cited in Chinsamy‐Turan & Ray, 2012). He reported FLB with some development of Haversian systems in four genera of gorgonopsians (*Aelurognathus*, Late Permian; *Scymnognathus*, now *Gorgonops*, Late Permian; *Dixeya*, now *Aelurognathus*; *Lycaenops*, Mid Permian; and one indeterminate). Ray, Botha & Chinsamy (2004) reported FLB from an additional genus, *Scylacops* (Late Permian) and found the cortices contained wide zones of FLB, interrupted by LAGs and annuli comprising avascular lamellar bone, suggesting rapid growth interrupted periodically. In contrast to *Scylacops*, they reported that *Aelurognathus* lacked these interruptions, suggesting its rapid growth was sustained. The osteohistology of gorgonopsians supports tachymetabolic endothermy and this is supported by reconstructions of them as active predators carrying themselves erect. The largest gorgonopsian *Inostrancevia* was quadrupedal, yet has a H–H distance consistent with endothermy (Fig. 3). Additionally, nutrient foramina from gorgonopsians indicate elevated metabolic rates consistent with endothermy (Knaus *et al*., 2021). There is thus good evidence that tachymetabolic endothermy was widespread in gorgonopsians (Proxies 1, 2, 3).

##### (iii) Dicynodontia (Middle Permian–Triassic)

Most studies of this long‐lived group of sturdy quadrupedal herbivores report bone histology implying high growth rates, i.e. FLB and Haversian remodelling, particularly during their growth phase and to varying degrees: *Lystrosaurus* (Late Permian–Early Triassic), *Oudenodon* (Late Permian), *Moghreberia* (Late Triassic), *Placerias* (Triassic), *Wadiasaurus* (Middle Triassic), and *Tropidostoma* (Late Permian) (Ray, Botha & Chinsamy, 2004; Ray, Chinsamy & Bandyopadhyay, 2005; Botha & Angielczyk, 2007; Ray, Bandyopadhyay & Bhawal, 2009; Botha‐Brink & Angielczyk, 2010; Green, Schweitzer & Lamm, 2010). These animals covered a size range from 0.5 m to the elephant‐sized, 4.5 m long *Lisowicia* (Late Triassic), weighing more than 8000 kg (Sulej & Niedźwiedzki, 2019). The bone histology of *Lisowicia* reflects uninterrupted fast growth, with a highly remodelled inner cortex. Olivier *et al*. (2017) used analyses of osteohistological attributes combined with phylogenetic eigenvector mapping to infer resting metabolic rates of three fossil synapsids, *Moghreberia*, *Lystrosaurus* and *Oudenodon*, and reported metabolic rates within the range for extant mammals and disjunct from a series of extant ectotherms. Additionally, Whitney & Sidor (2020) deduced from daily growth rings in tusk dentine that polar *Lystrosaurus* showed periods of seasonal torpor, whereas in non‐polar regions growth was uninterrupted. They noted (p. 472) that their results supported “the growing body of evidence that *Lystrosaurus* was endothermic”. More recently, nutrient foramina from dicynodonts have been found to indicate elevated metabolic rates consistent with endothermy (Knaus *et al*., 2021). Further support for endothermy among Dicynodontia comes from stable oxygen isotope analyses by Rey *et al*. (2017) who concluded that *Shansiodon* (Lower Triassic), *Lystrosaurus*, and *Moghreberia* were endothermic. Interestingly, they also concluded that *Dicynodon* (Late Permian) and *Oudenodon* were ectothermic, comparing oxygen isotope signatures with *Pareiasaurus*, which they assumed was ectothermic. However, on strong grounds we consider *Pareiasaurus* to have been endothermic (see Section V.1*a*) and an osteological study by Faure‐Brac & Cubo (2020) using eigenvector mapping listed *Oudenodon* as endothermic, along with *Lystrosaurus*. The evidence thus supports tachymetabolic endothermy in Dicynodontia (Proxies 1, 3, 5).

##### (iv) Therocephalia (Middle Permian–Middle Triassic)

Hillenius (1992, 1994) described respiratory turbinates in the Late Permian therocephalian *Glanosuchus* and therefore deduced their tachymetabolism. By contrast, stable oxygen isotope analyses by Rey *et al*. (2017) implied that *Glanosuchus* was ectothermic. However, their conclusion was derived from comparing its oxygen isotope signature with that of *Pareiasaurus*, assuming *Pareiasaurus* to be ectothermic. Extensive FLB was reported in *Pristerognathus* (Middle Permian) (Ray *et al*., 2004), supporting the earlier finding by de Ricqlès (1969), referred to by Chinsamy‐Turan & Ray (2012) in this genus and in *Theriognathus* (Late Permian). de Ricqlès (1969) also observed that Haversian bone was more extensive in the therocephalians than in gorgonopsians. Huttenlocker & Botha‐Brink (2013) reported FLB and moderately fast but interrupted multi‐year growth to large body size in Permian *Moschorhinus* but noted a change after the PTME to rapid sustained growth over a shorter period. Collectively, the evidence implies that tachymetabolic endothermy was common among therocephalians (Proxies 1, 6).

##### (v) Cynodontia (Late Permian–Early Triassic)

As reviewed by Botha‐Brink, Soares & Martinelli (2018), FLB appears to be typical across Cynodontia, including the early representatives, characteristically showing rapid bone deposition sustained through early to middle ontogeny. They considered this pattern of growth to be plesiomorphic for non‐mammaliaform cynodonts, having been found in even the most basal members of the clade. A similar growth pattern is reported from *Procynosuchus* (Late Permian) (Ray *et al*., 2004), *Galesaurus* (Early Triassic) (Botha‐Brink, Abdala & Chinsamy, 2012; Butler, Abdala & Botha‐Brink, 2019a) and *Thrinaxodon* (Early Triassic) (Botha & Chinsamy, 2005; Botha‐Brink *et al*., 2012). Structures in the nasal cavity of *Thrinaxodon* have been interpreted by Hillenius (1994) as respiratory turbinates. More recently, nutrient foramina from cynodonts have been found to indicate elevated metabolic rates consistent with endothermy (Knaus *et al*., 2021). Tachymetabolic endothermy thus was apparently widespread among Cynodontia (Proxies 1, 3, 6).

## VI DISCUSSION

Our proposal is that the whole‐body, tachymetabolic endothermy characteristic of extant birds and mammals is plesiomorphic to Amniota. The evidence we present and the multiple occurrences of it very early and widespread in amniotes are consistent with our hypothesis. This review synthesises numerous recent advances in the rarely integrated fields of biochemistry, cardiovascular physiology and palaeobiology to advance a well‐developed and coherent working hypothesis about the history and evolution of endothermy in amniotes. It may seem radical; it is a significant departure from the prevailing paradigm. However, to reprise the logic, the evidence we presented shows hitherto unappreciated strong similarities between the skeletal muscle mechanisms driving NST, a characteristic of tachymetabolic endothermy in birds and posited for the three extant mammalian clades. Thus, the capacity for endothermy could be older than the synapsid–sauropsid divergence. On that reasoning we predicted that some of the earliest sauropsid and synapsid clades for which adequate fossil samples exist should show evidence of tachymetabolic endothermy early in the amniote family tree. Assuming the validity of the multiple proxies for whole‐body (tachymetabolic) endothermy, that is what we found (Fig. 4). Indeed, we found evidence of endothermy in pareiasaurs and ‘pelycosaurs’, considered ancestral sauropsids and synapsids respectively, and in almost all the descendant major clades. The palaeontology results are consistent with and support our hypothesis, stemming originally from biochemical evidence, that tachymetabolic endothermy is homologous between mammals and birds and therefore plesiomorphic in Amniota.

Several issues need to be discussed further: responses to anticipated counter arguments, variations in the extent to which endothermy can be expressed, thoughts about the evolution of tachymetabolic endothermy, and the hypothetical structure of the heart and outflow tracts of the earliest tachymetabolic amniotes.

### (1) Deconstructing likely arguments in support of independent origins

The presumption that endothermy in mammals and birds evolved independently is deeply embedded. For example, Poelmann *et al*. (2014, p. 1) described it as “a textbook case of convergent evolution”. This is understandable because it has long been accepted that endothermy evolved in mammals and birds at very different times and with quite different sources of NST: BAT in mammals and skeletal muscle in birds. But these suppositions no longer apply. BAT is lacking in monotremes, marsupials and even in many eutherians (Section I.3), making it unlikely to be a shared ancestral mammalian trait. Having different times of origin no longer pertains as an argument either. It was thought that endothermy arose first among synapsids, within ‘mammals’ in the Late Triassic (e.g. McKenna & Bell, 1997), but later in Sauropsids, either in the Paleogene or Late Cretaceous, coincident with ‘modern’ birds (Neornithes). Subsequently the origin of birds, and presumably their endothermy too, was pushed back to the Palaeogene or Late Cretaceous and more recently still to the mid‐Cretaceous (Lee *et al*., 2014). However, in a comprehensive recent review, Benton (2020) concluded that (tachymetabolic) endothermy originated independently but more or less contemporaneously in sauropsids and synapsids in the Early Triassic, after the PTME. We confirm occurrences of endothermy in both sauropsids and synapsids in the Early Triassic, but our synthesis shows many occurrences of tachymetabolic endothermy in both groups very much earlier.

The presumption of separate origins for endothermy could also have been reinforced by quite striking differences between bird and mammal respiratory and cardiovascular systems, and these points may still be raised, so we discuss each of them in detail here.

#### (a) Respiratory systems: striking differences between extant birds and mammals

The tachymetabolism of endothermy requires rapid gas exchange, particularly during exercise, which is provided by the lungs of birds and mammals, but their anatomy and function are so different from each other that it would be easy to accept they had quite independent evolutionary origins. Indeed, this was the accepted view for a long time, until Hsia *et al*. (2013) drew attention to a dichotomy of views about their evolution. The older view was that the amniote lung was single‐chambered, as found in lissamphibians, the basal lepidosaurian *Sphenodon* and numerous squamates (Romer & Parsons, 1977), with the implication that multichambered lungs must have evolved separately in mammals, crocodylians, birds and varanid lizards. The alternative hypothesis (Duncker, 2004; Perry & Sander, 2004; Lambertz, Bohme & Perry, 2010) was that the plesiomorphic amniote lung was complex and multichambered and that mammals, turtles, and archosaurs all have multichambered lungs or derivatives thereof. This question has been settled by a comparative anatomical and embryological study by Lambertz *et al*. (2015). They found “shared structural principles of multichamberedness” (p. 1) recognisable across all amniotes, including lepidosaurs, from which they deduced that the “simple sac” of lepidosaurs is secondarily derived. They interpreted the more complex and multichambered lungs of the highly derived varanid lizards as a “reinvention” of the ancestral condition. In short, the evidence supports a multichambered lung being plesiomorphic for amniotes, well suited for efficient gas exchange in a terrestrial environment and ancestral to the highly derived respiratory organs of both mammals and birds.

#### (b) Cardiovascular systems: striking differences between extant birds and mammals

In extant sarcopterygian fishes, amphibians and the extant ectothermic amniotes (squamates, rhynchocephalians and turtles) the dorsal aorta is formed by dorsal fusion of both left and right 4th embryonic arches. In birds and mammals, however, there has been a ‘simplification’ which led to a striking difference between them. In mammals the posterior systemic circulation is derived from the left embryonic arch only, whereas in birds it is derived from only the right embryonic arch (Goodrich, 1958).

Does this obvious difference challenge our hypothesis that endothermy in mammals and birds is homologous? Our palaeontological survey showed that many of their ancestors too were endothermic (see Appendix S1), and if the acquisition of endothermy depended upon systemic blood supply *via* a single arch, that would have been characteristic of them all too. Unsurprisingly, very little is known directly about the cardiovascular anatomy of extinct sauropsids and synapsids, but we do, fortuitously, have a ‘Rosetta Stone’. Crocodylians have a four‐chambered heart (Fig. 5) and, on the basis of this and other evidence, Seymour *et al*. (2004) made a convincing case that they also have endothermic ancestry, a conclusion strengthened more recently by both osteological evidence (Cubo & Jalil, 2019) and embryological evidence (Poelmann & Gittenberger‐de Groot, 2019). Despite having a unique extra‐cardiac pulmonary bypass shunt that makes use of the left aorta (Section VI.4, Fig. 5), crocodylians at rest have both left and right aortae contributing to the systemic flow, suggested for *Caiman crocodilus* by White (1956) and confirmed in *Crocodylus porosus* by Grigg & Johansen (1987). Without the shunt operating, their circulation at rest is not very different from that of a bird or a mammal. Presumably the presence and operational use of both systemic aortae in crocodylians is indicative of the use of both aortic arches in their endothermic ancestry. This suggests strongly that the capacity for endothermy does not depend upon prior ‘simplification’ of the systemic blood supply. There is an abundance of independent evidence that tachymetabolism was basal in archosaurs (e.g. Seymour *et al*., 2004; Ezcurra *et al*., 2014; Ezcurra, 2016; Legendre *et al*., 2016; Cubo & Jalil, 2019; Benton, 2020; Cubo *et al*., 2020), so it is likely that at least all pseudosuchian occurrences of endothermy were supported by both left and right aortae, and perhaps that was so in their ancestors too.

Although there is no equivalent cardiovascular ‘Rosetta Stone’ among normal extant synapsids, all mammalian embryos go through stages with two aortic arches, and in rare human cases, either both arches or the right arch alone (as in birds) can persist after birth (Priya *et al*., 2018). There is no reason to postulate that the endothermy in some of the ‘pelycosaurs’ and early therapsids and all the other endotherms in the synapsid line was reliant solely on the left aortic arch. More likely, perhaps, the endothermic ancestry of today's birds and mammals had two arches contributing to their main posterior systemic supply until, over time, perhaps through millions of years of evolutionary ‘fine‐tuning’, each lost the contribution from one arch.

### (2) Variability in expression of tachymetabolic endothermy, and ‘reversions’

We recognise that extrapolating from the extant animals to extinct ones can be risky and caution is necessary, a point made also by Padian & de Ricqlès (2020). As a striking example of the risk, it led to the unfortunate interpretation that extinct reptiles were slow, low in energy intensity and ‘cold blooded’. Although reasonable at the time, it is very different from the reality revealed in recent years and reviewed and extended herein (Section V, Appendix S1). Despite the risk, we suggest two characteristics drawn from extant tachymetabolic endotherms that most likely occurred among historical endotherms.

#### (a) Different expressions of endothermy

The first is the capacity for whole‐body endothermy to be expressed in different ways. A comparison between two ancient but extant mammals provides a good example: platypus (*Ornithorhynchus anatinus*) and their distant cousin, short‐beaked echidnas, are monotremes. They represent the oldest of the three extant mammalian groups, which gives this example particular resonance. Although both are indisputably tachymetabolic endotherms, the thermal relations of both are different from most endotherms in having much lower metabolic rates and also lower *T*_b_ (reviewed by Nicol, 2017). They also have vastly different *T*_b_ patterns from each other. Platypus are continuously homeothermic at 31–32°C, even throughout winter in the coldest streams (Grigg *et al*., 1992b). However, *T*_b_ in short‐beaked echidnas normally varies by 2–5°C daily, sometimes more (Grigg *et al*., 1992a; Nicol, 2017). A stable *T*_b_, also of 31–32°C, characterises females incubating an egg (Beard & Grigg, 2000; Nicol & Andersen, 2006). Also, in contrast to platypus, short‐beaked echidnas indulge in torpor at any time of year (Grigg *et al*., 2004; Nicol, 2017), even to survive bushfire (Nowack, Cooper & Geiser, 2016) and show classical mammalian hibernation in winter (Grigg *et al*., 1989), rewarming with no visible movement early on, presumably using muscle NST (Grigg *et al*., 1992a; Nicol & Andersen, 2008). Multiple fossils with LAGs in long bones show that interruptions in growth rate were common in ancient endotherms too (Farlow *et al*., 1995; Padian & Horner, 2004), implying comparable occurrences of torpor or hibernation in harsh conditions, e.g. Köhler *et al*. (2012). Whitney & Sidor (2020) reported what appears to be flexibility in the expression of torpor or hibernation within an extinct genus, a common pattern in extant hibernators and torpidators. They deduced from the record written in tusk dentine that the Early Triassic dicynodont synapsid *Lystrosaurus* showed periods of seasonal torpor in a polar climate, whereas growth in non‐polar regions was apparently uninterrupted.

#### (b) Reversions from endothermy to ectothermy

The second characteristic to note is that the expression of the capacity for tachymetabolic endothermy appears to be reversible and may be opportunistic. It is usually assumed that, once evolved, endothermy becomes characteristic for that group and for descendant groups. However, endothermy is energetically expensive compared to ectothermy (Fig. 2), so reversions could result from an endotherm adopting a lifestyle or environment where endothermy is either not necessary or is too expensive.

For example, an active endothermic terrestrial predator that chases prey may evolve into an aquatic sit‐and‐wait ambush predator that no longer requires high aerobic stamina but relies instead on stealth and anaerobic burst activity to subdue prey. Prey species are attracted to the water's edge, which would be an ideal location for an ambush predator hiding under the water. Ectothermy would be selected for, because it reduces metabolic rate and lengthens dive duration that enhances stealth and enables the predator to drown endothermic prey quickly. It would also eliminate the need to stay warm against the great cooling power of water. This scenario may have applied to crocodylians and phytosaurs (Seymour *et al*., 2004; Legendre *et al*., 2016). Crocodylians are ectothermic, and phytosaurs probably were too; de Ricqlès, Padian & Horner (2003) likened their osteohistological patterns to those seen in living crocodylians.

Endotherms that occur in niches where food is insufficient to support full‐time endothermy often adopt energy‐saving strategies, notably periodic torpor. In extreme cases, endothermy can be essentially abandoned. For example, the blind Namib Desert golden moles (*Eremitalpa granti namibensis*) occupy the least productive terrestrial ecosystem on Earth, searching the sand dunes for patchily distributed insects. They are essentially ectotherms while resting and alert, thermal conforming with metabolic rates about 20% of that expected from other Insectivora (Fielden *et al*., 1990; Seymour, Withers & Weathers, 1998). Unlike all other moles that find prey by burrowing, Namib moles find prey by running on the surface, because sand‐swimming is energetically too expensive.

Some reversions to ectothermy may have occurred in the ancestors of the extant non‐crocodylian reptiles Lepidosauria (Rhynchocephalia and Squamata) and Testudines. If these groups are correctly placed within Sauropsida our hypothesis of a common ancestry for endothermy in sauropsids implies an ancestry capable of endothermy. Before discussing whether their ectothermy can be explained by reversion, we should note that the fossil record for very early amniotes is quite limited and the phylogeny is unresolved. Padian & de Ricqlès (2020) noted that the lack of phylogenetic context is a constraint for researchers speculating about the physiology of extinct vertebrates. There is a possibility that the lineages of extant non‐crocodylian reptiles may not fit well into Sauropsida, and their ancestors may not have had a capacity for endothermy. Nevertheless, assuming the extant non‐crocodylian reptiles do have that ancestry, could selective advantages have led to these essentially terrestrial reptiles reverting to ectothermy? Could the benefits of ectothermy in a Mesozoic world, in competition with the hugely diverse fauna at the time have been sufficient? With the focus on endothermy's success, it is easy to overlook the attributes of ectothermy, particularly its economical energy requirements, and the long lineages and enormous success of squamates and testudines in today's world. These testify to the many beneficial features of ectothermy so comprehensively addressed by Pough (1980). The ectothermy of these terrestrial reptiles does not rule out the possibility of endothermic ancestry, and it is worth recalling the very endotherm‐like attributes of the squamates Varanidae (Section IV.3) and Mosasauria (Section V.1*b*, Appendix S1). The benefits of ectothermy may also explain the reversion of notosuchians, an extinct sister group to Neosuchia, which includes crocodylians. They too apparently expressed ectothermy as a derived character from ancestrally endothermic archosaurs (Cubo *et al*., 2020). It must be noted that not all extant reptiles are ectothermic. The term ‘reptile’ is applied loosely to all Sauropsida, and that includes birds – extant reptiles that are endothermic.

Accompanying the reversion to ectothermy were changes in heart anatomy and function. Although the Crocodylia retain the four‐chambered heart of their endothermic ancestors, the extant rhynchocephalians, squamates and testudines do not. Their hearts are in fact more complex, with two atria and a ventricle comprising three chambers, the cavum venosum, cavum pulmonale and cavum arteriosum (Jensen *et al*., 2010) which, despite being anatomically connected, can achieve significant separation of arterial and venous blood through their internal architecture and timing (Grigg & Simons, 1972; Millard & Johansen, 1974; Shelton & Burggren, 1976; Wang *et al*., 2003). The embryological development of all amniote hearts is similar, with the final state essentially dependent on the growth of the interventricular septum (for details, see Seymour *et al*., 2004). Briefly, all amniotes pass through a stage where the septum grows towards the outflow vessels but initially does not separate the ventricle anatomically. In birds, mammals and crocodiles, the septum continues extending and separates the ventricle, but in slightly different ways. In a reversion from endothermy to ectothermy, however, abbreviation of the septum's growth sets up the situation seen in extant (non‐avian) reptiles. Interestingly, similar congenital ventricular septal defects occur in humans, including the Tetralogy of Fallot, and are not uncommon, showing that an evolutionary conversion of a completely divided heart into an incompletely divided one is not difficult to imagine. Such a shift would be more detrimental for a tachymetabolic endotherm than a bradymetabolic ectotherm. Hence, if their ancestry did include endothermy, the retention of this ‘defect’ in extant squamates and testudines could be expected, especially since it might confer several postulated advantages (Hicks & Wang, 1996; Burggren, Filogonio & Wang, 2020).

### (3) SERCA, muscle NST and the evolution of tachymetabolic endothermy

This review sets up a new frame of reference for thinking about the evolution of endothermy in amniotes. Instead of seeing its occurrences as a constellation of separate evolutionary events, we take a more parsimonious view, seeing its multiple occurrences as homologous and with a much more ancient heritage. Here we discuss when and how the capacity for the higher‐metabolic‐intensity lifestyle may have arisen.

#### (a) The evolution of endothermy in amniotes could have recruited pre‐existing processes

Walter & Seebacher (2009) noted that metabolic processes are highly conserved among vertebrates (Smith & Morowitz, 2004) and expressed the view that “the transition from ectothermy to endothermy is likely to involve quantitative changes in (existing metabolic) pathways rather than *de novo* structures or processes” (p. 2328), an observation extended by Seebacher (2020). In that context, the molecular mechanism generating extra heat within the SR of modern birds and mammals occurs by a broadly similar mechanism in today's teleost fishes and may be very old. Admittedly the Teleostei arose in the Early Triassic [370 million years ago (mya); Near *et al*., 2012] well after vertebrate tetrapods. However, the diversity of occurrences of SERCA‐based heat production in modern teleosts favours the possibility that similar examples of regional endothermy emerged within the Devonian ancestral ray‐finned fishes, the Actinopterygii (400 mya; Near *et al*., 2012) long before tetrapods. There are even examples leaning towards whole‐body endothermy in extant fish. Despite the constraints against retaining heat at a ‘whole‐of‐body’ scale for any animal living in water and breathing *via* gills, selection pressures for heat retention at that scale have apparently operated in fishes too. There is one particularly striking example. As mentioned earlier, opah (*Lampris* spp.) show highly developed regional endothermy, retaining heat generated as a by‐product from the “constant flapping of wing‐like pectoral fins” (Wegner *et al*., 2015, p. 786). This relies on extensive lipid deposits and even a counter‐current system in the gills to help retain heat (Runcie *et al*., 2009; Wegner *et al*., 2015), and a strong suggestion of SERCA activity in the pectoral muscles, uncoupled by SLN (Franck *et al*., 2019; Bal & Periasamy, 2020). With the emergence of amniotes from the water, no longer gill‐breathing and in a less thermally challenging medium, the evolution of whole‐body endothermy became more straightforward.

#### (b) The earliest amniotes

Little is known about the earliest amniotes, which were presumably ectothermic, living in the Late Carboniferous and generally depicted as small and probably agile terrestrial insectivores resembling today's lizards (Canoville & Laurin, 2010). Clarke & Pörtner (2010) portrayed them as small, active eurythermal ectotherms living in a cool but variable thermal environment. However, if we could be presented today with a freshly dead example for dissection, there is no reason to suppose its anatomy would be markedly different from the small reptiles of today. Nevertheless, the genomes of those early amniotes may have carried from their piscine ancestry *a capacity for heat production within muscle via SERCA*. We cannot tell whether the first expressions of endothermy pre‐dated or followed the separation of sauropsids and synapsids. The relevant fossil record is scant and mostly poorly preserved, but one preliminary study is of interest. Estefa *et al*. (2020) described well‐vascularised limb bones from two Early Permian Seymouriamorphs (either stem amniotes or a sister group) and deduced they showed faster bone growth and dynamics than Devonian stem tetrapods such as *Acanthostega*. There has been little work done and perhaps this review will encourage more attention to the fragmentary stem amniote record with this question in mind.

#### (c) The ectotherm to endotherm transition and muscle NST

The crucial change accompanying the transition from ectothermy to endothermy in amniotes was the very large increase in metabolic rate, both when active and at rest. This increases the capacity for sustained aerobic muscular work, while also providing by‐product heat for body temperature regulation, plus sufficient energy to support ST or, more efficiently, supplementary additional heat by NST when required. Compared with ectothermy as expressed by today's reptiles, the main changes underlying the transition involved the gain of insulation, higher aerobic metabolism associated with leakier mitochondria and many more of them, and a capacity for regulated metabolic heat production (ST and NST) (Else & Hulbert, 1987; Else, Turner & Hulbert, 2004). Whatever the original selection pressures were that brought about this transition, a high basal metabolic rate (BMR) and warm *T*_b_ became interdependent in the transition to tachymetabolism.

Numerous authors have tackled explanations for this transition (see reviews by Ruben, 1995; Clarke & Pörtner, 2010; Lovegrove, 2012, 2017; Polymeropoulos, Oelkrug & Jastroch, 2018; Legendre & Davesne, 2020; Seebacher, 2020) and a very engaging and wide‐ranging book by Lovegrove (2019). Three categories of hypothesis have dominated: body warmth advantages *per se*, in which warm and stable *T*_b_ benefits efficiency and counters cold conditions (Crompton, Taylor & Jagger, 1978; McNab, 1978); high metabolic rate advantages, which convey a greater capacity for sustained aerobic activity (Bennett & Ruben, 1979; Ruben, 1995); and the benefits of incubation and parental care (Farmer, 2000; Koteja, 2000, 2004), because endothermy facilitates survival of individuals through their most vulnerable phase. To those hypotheses can be added that tachymetabolic endothermy also allowed an increase in the upper limit to body size, by requiring high arterial blood pressure that, in turn, permits larger and taller bodies capable of accessing more of the available vegetation for food. These hypotheses are not mutually exclusive; different expressions of it may result from different selection pressures. However, any proposal inevitably identifies benefits attributable to endothermy in its now derived state, and possibly none of these benefits was the attribute selected initially. Identifying initiating selection pressures is seldom straightforward. Seebacher (2020) warned about teleological traps, and Koteja (2004, p. 1043) characterised the various proposals as attempts to identify the hypothetical initial trait “that could be subject to a direct selection, (and) which resulted in a correlated change of basal metabolic rate”.

Bennett & Ruben (1979) suggested that the selective advantage behind the evolution of endothermy was not thermoregulation, as originally suggested (Crompton *et al*., 1978; McNab, 1978), but an increase in aerobic capacity to support sustained activity, and that recognition was subsequently encapsulated in the ‘aerobic capacity hypothesis’. Physical activity is ubiquitous among amniotes, in all environments, and must have been so throughout our history. Anaerobically fuelled activity is limiting, so mechanisms that enhance prey capture and escape from predators or in defence against predation are eminently ‘selectable’. Koteja (2004) referred to Bennett & Ruben (1979) as a “milestone”, because it suggested that a high capacity for sustained physical activity could evolve as a by‐product of natural selection for a behavioural trait not directly related to thermoregulatory capabilities, and providing ‘waste’ heat incidentally, available for thermoregulation. Raising *T*_b_ was a collateral benefit. As presently understood, NST is not envisioned as being a routine contributor to body heat in the thermal neutral zone but functioning only during cool conditions to supplement by‐product heat from the body's maintenance and activity metabolism (Nowack *et al*., 2017). However, most of what is accepted about NST has been framed around studies on eutherian mammals with BAT. Little is known so far about the operational aspects of muscle NST, although it does seem to have a similar function in newborn wild boar piglets, which lack BAT (Nowack *et al*., 2019). Whether SERCA‐based heat production was a more direct contributor to endothermy in its earliest phase is unexplored so far but given its multiple occurrences in fishes (see Section III), as it may be in the deep muscle tissue of the regionally endothermic opah (Franck *et al*., 2019), that is possible. Seebacher (2020) saw endothermy as the evolutionary by‐product of energy balance regulation and interpreted its evolution in the context of metabolic networks. It is not far‐fetched to suggest a role for SERCA‐based heat production early in the evolution of endothermy, perhaps in concert with other elements contributing to the increase in metabolic intensity and sustainable aerobic capacity characteristic of tachymetabolic endotherms.

Nowack *et al*. (2017) suggested that muscle NST may have been an important step in the evolution of endothermy, perhaps as the first metabolic pathway in mammals to be used solely for thermogenesis. Because its importance in mammalian endothermy was recognised only recently, however, none of the current hypotheses seeking to explain the large increase in metabolic intensity and *T*_b_ between ectothermy and endothermy takes it into account. Doing so is beyond the scope of this review, but with endothermy's putative SERCA‐based regulatory heat source derived from and located within the locomotor musculature and arguably being homologous in sauropsids and synapsids, it does seem reasonable to propose that muscle NST will be judged in future studies to have been very significant in the evolution of tachymetabolic endothermy.

### (4) The heart of the earliest endothermic amniotes

Our hypothesis that endothermy in amniotes is plesiomorphic has implications for ideas about the evolution of the amniote heart. Researchers have routinely and reasonably assumed that undivided or partially divided hearts similar to those of extant amphibians and non‐crocodylian sauropsids are evolutionary antecedents of the four‐chambered hearts of mammals, birds and crocodiles. These three four‐chambered hearts are usually presented as textbook examples of convergent evolution (e.g. Poelmann *et al*., 2014), whereas we see them as homologous. The earliest endothermic amniotes must have had hearts that met the same basic functional criteria for endothermy seen in present‐day birds and mammals. Their hearts must have provided a high MAP in the systemic circuit because that is crucial to support the high intensity of aerobic metabolism characteristic of endothermy (Figs 2 and 3). The hearts must also have maintained a much lower pressure in the pulmonary system to avoid filtration of fluid into the lungs causing pulmonary oedema (Section IV.2). Only a four‐chambered heart could have achieved this, and such a heart would also have had the required capacity to separate oxygenated and deoxygenated blood traversing the heart. The hearts of the earliest endothermic amniotes would have been four‐chambered.

The four‐chambered crocodylian heart, mentioned briefly in Section VI.1*b*, is highly relevant to speculation about the heart of the earliest endothermic amniotes; although extensively modified, it provides the best ‘remnant’ example of early amniote endotherms. The modifications are thought to be a consequence of crocodylians having undergone a modification from basal endothermy to ectothermy in adapting to a lifestyle primarily as ambush predators that drown their prey (Seymour *et al*., 2004). It is significant that there are no extant aquatic endotherms that are sit‐and‐wait predators in water. On the contrary, the low aerobic metabolic rate of ectothermy promotes long breath‐holding while resting, taking refuge, or waiting under water for prey. Along with their adaptation to this lifestyle, the crocodylians' ancestral four‐chambered hearts and outflow tracts have gained several unique and novel modifications (Fig. 5E) which, in combination, allow the left systemic arch to facilitate pulmonary by‐pass shunting (PBS), now discussed briefly. One of these modifications is that instead of the left aorta exiting alongside the right aorta from the left ventricle, it exits from the right ventricle beside the pulmonary aorta (Fig. 5E). Associated modifications are the foramen of Panizza which provides a connection of controllable diameter between the left and right systemic aortae at their base, and a controllable ‘cog‐tooth valve’ at the exit from the right ventricle to the pulmonary artery (Fig. 5E). In other words, crocodylians have a four‐chambered heart with what appears to be an ‘added on’ external PBS. To explain the PBS very simply: in tandem with a slowing of the heart, partial constriction of the cog‐tooth valve reduces pulmonary blood flow by increasing the resistance to flow into the pulmonary artery. This also increases right ventricular pressure, so opening the valve to the left aorta that is normally closed and shunting some of the ‘venous’ blood into the systemic circulation (Grigg & Johansen, 1987; Axelsson *et al*., 1996, 1997). Some of this blood passes through the foramen of Panizza into the right aorta. The result is that the PBS lowers the oxygen level in the whole body, which is known to reduce metabolic rate in reptiles. This very likely allows crocodylians to extend their dive time when they rest or seek refuge for long periods underwater (Grigg, 1989). Shunting has been observed in captive *C. porosus* diving voluntarily (Grigg & Johansen, 1987) but not yet in natural situations on animals large enough to have the cog‐tooth valve developed; it is not present in hatchlings but was present in a series of 1.1–4.2 m animals (Webb, 1979). In crocodylians at rest, however, without the PBS in use, the left aorta fills from the right aorta *via* the foramen of Panizza during diastole and the cardiovascular system operates essentially like the double‐circuit systems seen in birds and mammals. Grigg & Kirshner (2015) provide a comprehensive review and discussion of the crocodylian heart, including the debate about its probable functional significance (pp. 299–303). The uniquely crocodylian ‘external PBS’ provides a similar capacity for shunting blood flows between pulmonary and systemic circuits as the ‘within heart’ shunting that occurs within the three‐chambered hearts of extant non‐crocodylian reptiles, possible because of their incompletely divided ventricle (Jensen *et al*., 2010) (Section VI.2*b*).

Importantly, embryological data confirm that the unusual modifications that comprise the PBS, the foramen of Panizza in particular, are secondarily derived and are likely to have evolved in parallel with the adoption of a semi‐aquatic lifestyle (Seymour *et al*., 2004). In short, without its unusual specialisations, the crocodylian heart provides a very satisfactory model for the heart of the earliest tachymetabolic amniotes (Fig. 5A, B). Such a heart is probably representative of the structure of the hearts of the pseudosuchians at least, and probably many more of the now‐extinct endothermic amniotes too, until somewhere in the many millions of years since the first endothermic heart, one or other of the 4th arches was lost, resulting in the systemic circuit ‘simplification’ discussed above in Section VI.1*b* and seen in today's mammals and birds (Fig. 5C, D).

## VII FUTURE RESEARCH

Our proposal that whole‐body endothermy is an ancestral feature shared by extant birds and mammals and that its occurrences were, and remain, widespread in contemporary Amniota raise many interesting research questions. We mention below only a few. A high priority is to determine the source of NST in monotremes, the most ancient group among extant mammals; we suspect that it is in skeletal muscle, as in marsupials. Another high priority is to determine the relative contributions of the two apparent sources of NST in eutherians, an ancient one in muscle and a more recent one in BAT, and under what circumstances and how much each contributes and how that is coordinated. A phylogenomic approach to the evolution of endothermy could be very revealing, including comparisons between the genomic background to muscle NST in birds and mammals, and exploring the possibility that some of the relevant genomic transcripts are shared with vertebrates expressing regional endothermy as well, including fishes. Since crocodylians show strong evidence of endothermic ancestry (Seymour *et al*., 2004), would a phylogenomic study reveal latent genes for muscle NST? Another area that beckons is to search for other examples of SERCA‐based heat production. For instance, the source of heat in an agamid lizard, the tegu (*Tupinambis merianae*), during incubation (Tattersall *et al*., 2016) remains enigmatic. Also among reptiles, some pythons ‘shiver’ to warm their eggs (Hutchison *et al*., 1966; Harlow & Grigg, 1984), can we be sure that no SERCA‐based heat production is also involved? And is the warmth of the tuna red lateral musculature enhanced by SERCA? Another possibility comes from a study of free‐ranging leatherback turtles reporting *T*_b_ averaging about 26°C in waters between Nova Scotia and Newfoundland with a surface temperature around 15°C (Casey, James & Williard, 2014). This is a large thermal gradient for an aquatic ectotherm to maintain as a consequence of aquatic gigantothermy at that size (Brice & Grigg, in press), plus by‐product heat from the viscera and swimming muscles and their partial insulation. Finally, more osteological and other data could be gleaned from skeletons of the earliest synapsids and sauropsids.

## VIII CONCLUSIONS

1. The stimulus for this review came from recent studies positing that the ancient source of regulatory non‐shivering thermogenesis (NST) in mammals is in skeletal muscle and not, as has long been accepted, from UCP1 located in brown adipose tissue (BAT). If true, a skeletal muscle origin explains the hitherto mysterious source of regulatory NST in extant Monotremata and Marsupialia, and in so many Eutheria too, which lack both UCP1 and BAT. Also, this implies that thermogenic UCP1 and BAT are more recent, originating since the separation of marsupials and eutherians. But most intriguingly, a skeletal muscle origin of NST aligns mammals with birds; they too lack UCP1 and BAT and use skeletal muscle NST. This prompted our hypothesis that birds and mammals share tachymetabolic endothermic ancestry, contrary to the apparently unchallenged tenet that endothermy evolved in them independently.
2. The biochemical process proposed to be driving muscle NST in birds and many, perhaps most, mammals is the ‘slippage’ of Ca^2+^ from the sarcoplasmic reticulum Ca^2+^‐ATPase system (SERCA) in skeletal muscle. If endothermy in mammals and birds is homologous, as we propose, the detail of that process should be very similar in both. After undertaking a detailed analysis of the similarities and differences between the SERCA process and its control in bird and mammal muscle NST, and considering the multi‐millions of years since their ancestry diverged, we found the SERCA processes to be sufficiently similar to be consistent with their tachymetabolic endothermy being homologous.
3. That being so, we reasoned that tachymetabolic endothermy would have occurred very early in the amniote family tree. Accordingly, we compiled from the palaeontological literature an inventory of probable occurrences of whole‐body endothermy in sauropsids and synapsids, paying particular attention to clades that originated earliest and that are not normally considered endothermic. As well as noting conclusions made by the researchers themselves, we also made our own interpretations, considering six different proxies for tachymetabolism, including osteohistology, cardiovascular physiology, reconstructed body form and other relevant anatomical features and, with caution, palaeothermometric data.
4. In Sauropsida, our findings support the growing acceptance that basal Archosauromorpha were tachymetabolic endotherms and our inventory includes additional occurrences among Pareiasauria, Squamata (mosasaurs), Plesiosauria and Ichthyosauria. In Synapsida, it has been accepted that endothermy arose within Permian therapsids. We found convincing evidence for it in Permian dinocephalians and gorgonopsians and also in Permian–Triassic dicynodonts, therocephalians and cynodontians. Further back, we found evidence for it in Late Carboniferous ‘pelycosaurs’ (edaphosaurs, an ophiacodontid and sphenacodontians). These occurrences are consistent with tachymetabolic endothermy being characteristic of amniotes and plesiomorphic.
5. Strikingly different expressions of tachymetabolic endothermy between the extant platypus and echidna, both monotremes and the oldest extant mammals, suggest the likelihood of a similar diversity in energetic intensity among extinct endotherms too, as well as lower and more variable *T*_b_ and capacities for torpor and hibernation. Also, extant crocodylians and extinct phytosaurs and notosuchians provide probable examples of ancestral endothermy being lost, and similar reversions from an ancestry with a capacity for endothermy may account for the ectothermy of living non‐avian reptiles today. There may have been many such examples in the nearly 300‐million‐year amniote history of endothermy being expressed to different extents or selected against.
6. The crucial changes accompanying the evolution of tachymetabolic endothermy were the very large increase in the capacity to sustain aerobic work by the muscles and capacities for both shivering thermogenesis (ST) and non‐shivering thermogenesis (NST), the latter apparently based on ancient muscle biochemistry supplying heat for body temperature regulation. Current proposals to account for the evolution of tachymetabolic endothermy in amniotes do not yet take muscle NST into account and many implications from its recognition remain to be explored.

## Supporting information


**Appendix S1.** Probable occurrences of tachymetabolic endothermy across Sauropsida and Synapsida.
