# Supplementary material for: Whole‐body endothermy: ancient, homologous and widespread among the ancestors of mammals, birds and crocodylians
Source: Biol Rev Camb Philos Soc. 2021 Dec 10;97(2):766–801. doi: 10.1111/brv.12822 (PMC9300183; doi:10.1111/brv.12822)
Supplement: Supplementary file 1 — Appendix S1. Probable occurrences of tachymetabolic endothermy across Sauropsida and Synapsida. [file BRV-97-766-s001.docx]

**SUPPORTING INFORMATION**

**APPENDIX S1. PROBABLE OCCURRENCES OF TACHYMETABOLIC ENDOTHERMY ACROSS SAUROPSIDA AND SYNAPSIDA**

Here we summarize results from a literature search evaluating evidence of likely occurrences of whole-body, tachymetabolic endothermy among most of the major clades of amniotes. We have no doubt that some of our conclusions will be challenged, but we also have no doubt that the overall picture they paint is very strong.

**Introductory notes.** Throughout, we sought information from multiple sources and in the report below the proxies on which each conclusion in support of tachymetabolic endothermy was reached are identified. The proxies we used as evidence for endothermy are explained fully in the main text (Section IV) and, for convenience, are listed here. Figures referred to are in the main text, with Fig. 4 providing an indicative but not comprehensive summary of the survey’s results.

Proxy 1 – evidence from osteohistology.

Proxy 2 – evidence from central cardiovascular physiology.

Proxy 3 – evidence from long bone foramina; femur blood flow index.

Proxy 4 – evidence from the biomechanics of bipedality.

Proxy 5 – evidence from palaeothermometry.

Proxy 6 – evidence from respiratory turbinates.

Most of the six proxies we used are straightforward. Diagnosing tachymetabolic endothermy using only osteohistological data, however, can be challenging because reports of endothermy based on fibrolamellar bone (FLB) can be open to question unless they indicate that a high rate of growth has been sustained throughout a substantial part of ontogeny (Farlow, Dodson & Chinsamy, 1995; Padian & de Ricqlès, 2020). We took that advice seriously. In our literature survey, osteohistological information was the most common category of data available, but only in eight out of nearly 50 taxa were there no additional proxies available. One of these (phytosaurs) we judged as most likely ectothermic, and we judged the archosauromorph *Aenigmastropheus* as endothermic but show it in Fig. 4 with a question mark because the evidence is insufficiently strong. In the remaining six, the evidence in support of tachymetabolic endothermy is strong by itself. Another point to note is that, in some taxonomic categories, studies on different genera provided conflicting results, with some implying ectothermy and others endothermy. However, as we discuss elsewhere (Sections IV, VI.2) we do not hold the view that, once expressed, endothermy is necessarily present throughout that taxonomic grouping, or its descendants. At least three ‘reversions’ from endothermy to ectothermy have been proposed: in crocodylians (Seymour *et al*., 2004), phytosaurs (Legendre *et al*., 2016) and notosuchians (Cubo *et al*., 2020). Also, phylogenetic associations seem to be less stable than generic designations. Accordingly, wherever we found an occurrence of tachymetabolic endothermy we scored it and identified it in Fig. 4.

Note that, for completeness, we replicate here from the main text our diagnoses for the Parareptilia (early Sauropsida), ‘Pelycosaurs’ and pre-Triassic Therapsida (early Synapsida), the amniote groups most relevant to our study.

**STEM AMNIOTES AND PUTATIVE SISTER GROUPS**

Estefa *et al.* (2020) described well-vascularised limb bones from two Early Permian seymouriamorphs, *Seymouria sanjuanensis* and *Discosauriscus austriacus* (either stem amniotes or sister groups). They sought information on rates of growth to compare with Devonian stem tetrapods (i.e. non-amniotes) that are thought to have been mostly aquatic or semi-aquatic and to have taken many years to reach maturity. Specifically, they sought evidence that seymouriamorphs might represent a first step towards a more active metabolism, and perhaps much faster progress to sexual maturity, as was deduced previously for the Late Carboniferous synapsid *Ophiacodon* (Shelton & Sander, 2017). In both seymouriamorphs, the study revealed limb bones to be well vascularised, with evidence of faster bone growth and dynamics than in stem tetrapods such as *Acanthostega* and early temnospondyls. However, the results were inconclusive about the time taken to reach maturity. The authors encouraged exploration of other stem- or early amniotes such as the Late Carboniferous–Late Permian diadectids.

**SAUROPSIDA**

**Parareptilia (Anapsida)**

**Procolophonidae —** (Late Permian –Late Triassic). Small (30–50 cm) lizard-like ‘parareptiles’. Botha-Brink & Smith (2012) studied the limb bone histology of three small Triassic procolophonid parareptiles, *Sauropareion anoplus* (Early Triassic), *Procolophon trigoniceps* (Late Permian–Early Triassic) and *Teratophon spinigenis* (Middle Triassic), from the Karoo Basin of South Africa, in order to infer their palaeobiology. No FLB was observed, but they noted the bone histology was different from typical reptile bone, and well vascularised in a couple of genera, to the extent that suggested rapid growth early in development. They considered the morphology and bone histology to suggest a burrowing lifestyle. From the available information, no conclusion can be reached about their metabolic status.

**Pareiasauridae** (Middle–Late Permian ‘parareptiles’). Many were large (1–3 m long, 100–600 kg) stocky herbivores with semi-erect to upright stance. They have been reconstructed as chunky-looking herbivores, somewhat resembling domestic cattle. Canoville & Chinsamy (2017) examined samples from the long bones and ribs of several South African pareiasaurs, including *Pareieasaurus*, *Pareiasuchus*, *Bradysaurus* and *Anthodon* (all Late Permian) and found a generally similar microstructure among them*.* They found no FLB, but extensive well-vascularised Haversian systems with primary and secondary osteons. They concluded that the bone histology indicated relatively rapid growth early in ontogeny, with periosteal growth slowing later and growth continuing for several years during adulthood. They suggested that the early high growth “could possibly be used in support of previous interpretations by de Ricqlès (1978*a*) that pareiasaurs may have had intermediate physiologies as compared to other basal amniotes, with a tendency towards endothermy” (p. 1063). Looy *et al.* (2016) studied the osteohistology of limb bones and a scapula of *Bunostegos* (Late Permian) from Niger. They interpreted the results as evidence of fast bone deposition and elevated metabolism, in accordance with previous studies on pareiasaurs (de Ricqlès, 1978*a*,*b*; Canoville & Chinsamy-Turan, 2011). These interpretations are congruent with the semi-erect to upright limb posture of pareiasaurs (Bakker, 1971; Sumida & Modesto, 2001; Turner *et al.*, 2015). After histological examination of bone from two Upper Permian pareiasaurs from Russia, *Deltavjatia rossica* and *Scutosaurus karpinskii*, Boitsova *et al.* (2019) reported relatively short periods of rapid growth early in life, with well-vascularised, fast-growing FLB separated by lines of arrested growth (LAGs). Although this rapid growth window was relatively short, it apparently accounted for about 50% (*Deltavjatia*) or 75% (*Scutosaurus*) of their growth to maximum size. A transition to poorly vascularised parallel-fibred and lamellar bone separated by LAGs followed, suggesting that slower but still periodic growth continued for several years into adulthood. By contrast, much slower growth was inferred in the Middle Permian *Provelosaurus americanus* from southern Brazil (Farias, Schultz & Soares, 2019). Following the logic of Seymour (1976, 2016), the body size and habitus of the larger pareiasaurs such as *Pareiasaurus*, *Scutosaurus* and *Bunostegos* imply a heart-to-top-of-body (H–H) distance requiring endotherm-equivalent mean arterial pressure (MAP) (Fig. 3) and a four-chambered heart (Fig. 5). Moreover, Rey *et al.* (2020) reported stable oxygen isotope values from phosphates in teeth and bone from *Bradysaurus* and some unidentified pareiasaurs. Used as a proxy for water dependence, the values clustered with those from coeval tachymetabolic endothermic synapsids. Collectively, satisfying Proxies 1, 2, and 5, there is strong evidence for the occurrence of tachymetabolic endothermy in Pareiasauridae.

**Non-archosauromorph diapsids**

(Ichthyopterygia, Plesiosauria, Mosasauria)

Adults of some genera in each of these three groups of aquatic sauropsids grow to very large size, so large that gigantothermy has to be considered when palaeothermometric data provide body temperature estimates that might imply tachythermic endothermy. Aquatic gigantothermy differs from terrestrial gigantothermy because there is no daily input of solar radiation. Prompted by two palaeothermometric studies on these three aquatic sauropsids (Bernard *et al.*, 2010; Harrell, Perez-Huerta & Suarez, 2016) (see below), Brice & Grigg (in press) undertook the first quantitative analysis of aquatic gigantothermy. They aimed to determine how large a bradymetabolic air-breathing aquatic sauropsid would need to be to maintain an endotherm-like body temperature over a specified range of water temperatures, with feasible amounts of insulation and substantial daily activity. Briefly, the modelling showed that, if adult, none of the animals in the Bernard *et al.* (2010) and Harrell *et al.* (2016) studies could have achieved the reported body temperatures across the range of temperatures prevailing in their habitat by either regional endothermy or gigantothermy. Tachymetabolic endothermy provides the more parsimonious explanation, endorsing the options chosen by the relevant authors in each study. This conclusion is validated by multiple independent osteohistological studies which inferred high rates of growth and a high metabolic rate in representatives of each group. It is important to note that, as recognised by Reid (1997) and shown by Seymour (2013), warmth gained by gigantothermy produces a warm ectotherm with low aerobic metabolic scope, not a tachymetabolic endotherm capable of sustained aerobic activity. For each of the three clades we provide a detailed review and discussion below.

**Ichthyopterygia —** (Triassic–Late Cretaceous). Bernard *et al.* (2010) analysed stable oxygen isotopes in tooth enamel and/or cortical bone from three genera of ichthyosaur, using values from coeval fish as proxies for water temperature. They reported body temperatures averaging 35 °C ± 2 °C across water temperatures of 16–29 °C. The ichthyosaurs were *Platypterygius* (≅ 7 m, ≅ 1500 kg), *Ophthalmosaurus* (≅ 6 m, ≅ 900 kg) (Ophthalmosauridae) and *Mixosaurus* (≅ 1–2 m, ≅ to 25 kg) (Mixosauridae). The authors were aware of gigantothermy as a possibility for the largest animals but presented satisfactory counter arguments, concluding from the high and stable body temperature (*T*_b_), presumed active predatory lifestyles and osteohistological evidence of high growth rates, that ichthyosaurs had “some kind of endothermy” (p. 1382). The modelling undertaken by Brice & Grigg (in press) implies that ichthyosaurs of this size, if bradymetabolic, would have been unable to maintain the reported *T*_b_ across the prevailing 16–29 °C temperature range, even if adult and with substantial insulation and activity. Brice & Grigg (in press) concluded that tachymetabolic endothermy provides a much more plausible explanation than either regional endothermy or gigantothermy for the palaeothermometry results.

There is considerable evidence from bone histology for tachymetabolic endothermy in quite a few ichthyosaurs, with an abundance of FLB as evidence of high growth rates (de Buffrénil & Mazin, 1990; Houssaye *et al.*, 2014; Anderson *et al.*, 2018). Kolb, Sánchez-Villagra & Scheyer (2011) reported FLB in the humeri of the comparatively small *Mixosaurus* and noted that the implied high growth rate indicated that “higher metabolic rates characterised small, non-thunniform ichthyosaurs, as has been suggested already for post-Triassic, cruising forms” (p. 402). In an even more basal ichthyopterygian, the 2–3 m Early Triassic *Utatsusaurus hataii*, Nakajima, Houssaye & Endo (2014) reported the presence of FLB, suggesting a high growth rate and “a trend towards homeothermy” (p. 343) (presumably endothermy in our terminology). More recently, Anderson *et al.* (2018) examined the histology of multiple samples of post-cranial bone from *Stenopterygius quadriscissus* and reported FLB as well as Haversian canals from multiple sites. However, although acknowledging from their own and previous studies that “Multiple lines of evidence …support the potential for a raised metabolic rate and thermoregulation in *Stenopterygius*” (p. 13–14), they preferred to attribute any raised metabolic rate to regional endothermy: “The mechanism of thermoregulation probably involves retention of heat produced in the muscles during sustained swimming, resulting in a raised ectotherm or homeostatic ectotherm state.” (p. 14). The subsequent analysis by Brice & Grigg (in press) shows that *T*_b_ values in the mid-30s °C could not have been maintained by bradymetabolic aquatic sauropsids the size of even adult *Stenopterygius* (3–4 m) if they were ectothermic, either by gigantothermy or regional endothermy. Also relevant is the report of a sub-cutaneous fibro-adipose layer in a small (1.6 m) and “exquisitely preserved” *Stenopterygius* (Lindgren *et al.*, 2018). We conclude that ichthyosaurs were tachymetabolic endotherms (Proxies 1 and 5, supported by analysis precluding gigantothermy), and were probably similar in many ways to today’s dolphins.

**Sauropterygia —** (Plesiosaurs and Pliosaurs, Early Triassic – Late Cretaceous). Bernard *et al.* (2010) analysed stable oxygen isotopes in tooth enamel and/or cortical bone to estimate the temperature at which it was laid down in four genera of plesiosaur compared with coeval fish as proxies for water temperature. The plesiosaurs were *Liopleurodon* (≅ 6–7 m) and *Polyptychodon* (≅ 7 m) (Pliosauridae), *Cryptoclidus* (≅ 4–8 m) (Cryptoclididae) and *Zarafasaura* (≅ 3–4 m) (Elasmosauridae). Body temperatures averaged 35 ± 2 °C across water temperatures of 16–23 °C. Adults of these ranged from 3 to 7 m, with much of the body length comprising neck. Could the high *T*_b_ be the result of gigantothermy? As for ichthyosaurs, the authors referred to the presumed active, predatory lifestyles implied by plesiosaur skeletal features and concluded that the high and stable *T*_b_ over a wide range of ambient temperatures was a consequence of “some kind of endothermy” (p. 1382). From the modelling undertaken by Brice & Grigg (in press) and given that much of the reported body length of a plesiosaur comprises neck, these four species, if bradymetabolic, would all have been too small for either regional endothermy or gigantothermy to explain a *T*_b_ even 6 °C above water temperature, let alone the reported 12–19 °C gradients.

A conclusion in favour of tachymetabolic endothermy is supported by the widespread occurrence in plesiosaurs of well-vascularised FLB and Haversian remodelling, including in adults (Wiffen *et al.*, 1995; Klein, 2010; Krahl, Klein & Sander, 2013; Wintrich *et al.*, 2017; Fleischle, Wintrich & Sander, 2018; O’Keefe *et al.*, 2019). Klein *et al.* (2015) noted that, although the situation in a basal sauropterygian *Nothosaurus* (Nothosauroidea) is less clear (Krahl *et al.*, 2013), the Triassic *Placodus* and some, but not all, other Placodontia, possibly even more basal ones, have FLB and may have had metabolic rates typical of birds and mammals. Using their method for quantifying bone vascularity, Fleischle *et al.* (2018) looked at eight sauropterygians and concluded that all were endothermic, with plesiosaurs having resting metabolic rates equivalent to those of birds and the more basal clades having mammalian rates. They noted that this conclusion is supported by their viviparity as well as the fast growth rate of juveniles as judged by growth marks and bone apposition as reported by Wintrich *et al.* (2017). Noting that placodonts are the most basal sauropterygians, Fleischle *et al.* (2018) suggested that elevated metabolic rate and high growth rate may have evolved at the base of Sauropterygia “or may have even been inherited from terrestrial progenitors” (p. 15). The large size of plesiosaur propodeal nutrient foramina suggests high metabolic rates, although basal sauropterygians have smaller foramina (Wintrich & Sander, 2019). In short, we found good evidence for tachymetabolic endothermy in plesiosaurs, satisfying Proxies 1 and 5, and backed up by analysis precluding gigantothermy.

**Lepidosauria**

**Mosasaurs —** (Squamata, Cretaceous). Mosasaurs were wholly aquatic, viviparous predatory sauropsids with a fusiform body shape tapering more posteriorly into a slender tail turned down within a vertical tail fin (hypocercal) that is thought to have provided most of the propulsive force. Preying on fish and ammonites, directional control was achieved with paddles modified from limbs. Adults ranged in size from 2 to 12 m at least.

There have been two separate studies applying stable oxygen isotope methodology to mosasaurs. In the first, Bernard *et al.* (2010) analysed stable oxygen isotopes in teeth or cortical bone from three mosasaur genera, *Platecarpus* (≅ 6–12 m, up to 8,500 kg), *Prognathodon* (≅ 5–10 m, up to 6,000 kg) and *Tylosaurus* (up to 12 m, 8,500 kg) from five Late Cretaceous locations and also from coeval fish at each location as proxies for water temperature. Body temperatures were in the range of 35–39 °C across water temperatures of 14–29 °C, and *T*_b_ appeared more dependent on ambient temperature in mosasaurs than in the ichthyosaurs and plesiosaurs in the same study. With large thermal gradients to the ambient water, and body form and dietary information suggesting a fast-swimming predatory lifestyle, the authors concluded that mosasaurs had “some kind of endothermy” (p. 1382). In the second palaeothermometric study, Harrell *et al.* (2016) derived *T*_b_ data for three genera of Upper Cretaceous mosasaurs, a coeval fish (*Enchodus*) and a bird (*Ichthyornis*). They reported mean *T*_b_ = 33.1°C for *Clidastes* (3 m, ≅ 100 kg), 36.3 **°**C for *Platecarpus* (6 m, ≅ 900 kg) and 34.3 **°**C for *Tylosaurus* (9 m, ≅ 3300 kg), all much warmer than the fish (*T*_b_ = 28.3 **°**C) and close to the bird (*T*_b_ = 38.6 **°**C). Like Bernard *et al.* (2010), they concluded that mosasaurs were endothermic, arguing against gigantothermy because there was no correlation between *T*_b_ and body size across the three species. If bradymetabolic, the modelling by Brice & Grigg (in press) discussed above shows that the largest species of *Tylosaurus* (up to 12 m) and *Prognathodon* (to 10 m) could, as adults, have been homeothermic in the mid-high 30s °C by gigantothermy, at rest and without insulation or activity, in 28 °C water. The smaller *Clidastes* or a 6 m *Platecarpus* could not. With sufficient insulation and a very high proportion of its day spent active, however, gigantothermy could allow a 6 m (≅ 900 kg) *Platecarpus* to maintain 34 °C in 20 °C water, but with the possible exception of *Tylosaurus*, none could have maintained the reported *T*_b_ at the low end of the range of water temperatures reported by Bernard *et al.* (2010), 14–29 °C. Moreover, without tachymetabolism, the growth of juvenile stages of even *Tylosaurus* would have been constrained to rates characteristic of ectothermic reptiles, which is contradicted by osteological evidence supporting high growth rates early in ontogeny. Houssaye *et al.* (2013) compared humeri from six genera of mosasaurs, including *Clidastes* and *Platecarpus*, with those from extant squamates, extinct aquatic reptiles and extant aquatic mammals. They reported an unusual type of parallel-fibred bone, which they suggested implied basal metabolic rates higher than in typical reptiles and intermediate between *Dermochelys* and rates suggested for plesiosaurs and ichthyosaurs. Accordingly, Houssaye *et al.* (2013) suggested that mosasaurs may have been gigantotherms, leading Faure-Brac & Cubo (2020) to note that the possibility of gigantothermy had not been excluded. In a separate study, Heingård (2014) reported abundant FLB in a vertebra and in a well-vascularised humerus of *Platecarpus*, which she interpreted as “evidence of elevated growth rate and a high metabolic rate compared to that of extant ‘reptiles’”. Taking into consideration the palaeothermometric *T*_b_ data and the inability of the modelling to explain those results if the animals had been bradymetabolic (Brice & Grigg, in press), along with high growth rates implied by bone histology, it seems safe to conclude that most, if not all, mosasaurs were tachymetabolic endotherms (satisfying Proxies 1 and 5, the later supported by analysis precluding gigantothermy).

**Non-archosauriform archosauromorphs**

(*Aenigmastropheus*, *Azendhosaurus*, Rhyncosauria, *Prolacerta*)

***Aenigmastropheus* *parringtoni*** **—** (Late Permian). This archosauromorph is known from a single specimen, mostly post-cranial, from Tanzania. It was medium-sized and probably lizard-like in shape. Histological examination of a limb bone by Ezcurra, Scheyer & Butler (2014) showed FLB in the deepest parts of the cortex with a woven bone matrix vascularised by circumferentially arranged longitudinal primary osteons, indicating high growth rates during early development. The outer part of the cortex comprised lamellar-zonal bone, weakly vascularised by scattered primary osteons and short simple vascular canals. In this outer part, LAGs showed eight growth cycles, indicating a much slower growth rate, and one interpretation by the authors was that the closely spaced LAGs indicated that the animal may have had a prolonged life, but perhaps constrained by harsh conditions. They also noted that the growth pattern seen in *Aenigmastropheus* resembles those seen in *Prolacerta* and the basal archosauriforms *Proterosuchus*, *Chanaresuchus* and *Erythrosuchus,* for all of which there is good evidence of endothermy (see below). *Aenigmastropheus* was judged by Klein, Foth & Schoch (2017) in their Fig. 6 to be in their Category 2 [growth with FLB early in ontogeny, then lamellar-zonal bone (LZB)]. Rapid early growth, until approaching adult size, is characteristic of many endotherms. Presumably its high early growth rate depended on a higher than ectothermic metabolic rate. On the basis of osteohistology and phylogenetic bridging, *Aenigmastropheus* was very likely to have been endothermic, but more information is needed. It is identified in Fig. 4 as a tachymetabolic endotherm, with a question mark (Proxy 1).

***Azendohsaurus laaroussii* —** (Triassic). Another archosauromorph, *Azendohsaurus* was known originally from a single jaw, from Morocco, with considerable disarticulated post-cranial material collected subsequently. A related species is known from Madagascar and another from India. Cubo & Jalil (2019) examined histology of limb bones and found several bone types, including FLB, which provides evidence for high growth rates. Further quantitative analysis, including an innovative application of phylogenetic eigenvector mapping to compare attributes of bone histology between extant and extinct vertebrates and estimate metabolic rates of fossil archosauromorphs, was undertaken by Legendre *et al.* (2016). They showed *Azendhosaurus* with a high mass-specific resting metabolic rate, nesting within the range observed in extant mammals and providing evidence for tachymetabolic endothermy (Proxy 1).

**Rhynchosauria —** (Early to late Triassic). These were generally lizard-like archosauromorphs with a very diverse, worldwide distribution. Beginning at small sizes (0.5 m), larger species (up to 2 m) evolved in two groups independently as the Triassic progressed (Ezcurra, Montefeltro & Butler, 2016). The larger species were heavily built, and typical reconstructions show them resembling ‘high-walking’ fat, stumpy-tailed crocodiles with very broad heads and parrot-like beaks. On the criterion of extensive FLB for a substantial part of their ontogeny, at least some rhynchosaurs were probably whole-body endotherms. Mukherjee (2015) reported extensive FLB in *Hyperodapodon* from India and interpreted four distinct growth stages: a juvenile stage with fast and continuous growth; early and late sub-adult stages in which growth was still rapid but punctuated and with considerable avascular lamellar bone in the latter; and an adult stage with much slower growth. Veiga, Soares & Sayao (2015) conducted a histological study of two hyperpdapodontine rhynchosaurs, *Teyumbaita sulcognathus* and *Hyperodapedon*, and described a similar growth profile: “relatively rapid growth during early ontogeny, which then slowed during the subadult stage with periodic interruptions” (p. 835). They interpreted their observations as indicating that growth at high rates was already present in basal archosauromorphs. By contrast, Werning & Nesbitt (2016) found no FLB in *Stenaulorhynchus stockleyi*, suggesting slower growth (and lack of endothermy) in this genus. Klein *et al.* (2017) categorised rhynchosaurs in their Category 2 (growth with FLB early in ontogeny, which then switches to LZB). Tachymetabolism is highly likely to have characterised at least some rhynchosaurs on the basis of sustained high growth rates (Proxy 1).

***Prolacerta* —** (Lower Triassic). *Prolacerta* was a small (up to 1.5 m) lizard-like carnivore, possibly insectivorous, with hind legs longer than its forelegs. Botha-Brink & Smith (2011) reported poorly defined FLB, with parallel-fibred bone in some regions, and concluded that *Prolacerta* had a comparatively slow growth rate, comparable to extant crocodiles. However, that conclusion was apparently amended after consultation with J. Botha-Brink, by Legendre, Segalen & Cubo (2013) who in a study of *Euparkeria* included *Prolacerta*, *Erythrosuchus* and *Chanaresuchus* as examples of archosauromorphs with high growth rates and high metabolic rates “compatible with endothermy” (p. 1343). Subsequently Legendre *et al.* (2016) used eigenvector mapping to show that the resting metabolic rate was within the range of extant tachymetabolic endotherms. This was confirmed in further analysis by Cubo & Jalil (2019). (Proxy 1)

**Non-archosaurian archosauriformes**

(*Proterosuchus*, *Erythrosuchus*, *Euparkeria*, Phytosauria)

***Proterosuchus*** **—** (Early Triassic). This was a moderately sized (skull length up to 400 mm, total length up to 2.2 m) predatory archosauriform, crocodylian in body form, with the tip of its snout characteristically curved downwards over the lower jaw. It had a strong and muscular tail suggesting semi-aquatic habits in addition to walking on land with a semi-erect gait. Botha-Brink & Smith (2011) reported rapidly forming uninterrupted FLB early in its ontogeny, becoming slowly forming LZB with increasing age. Their interpretation was that rapid growth continued until two-thirds of adult size. These authors concluded that the growth rate of *Proterosuchus* was high and noted that this was consistent with the conclusion of de Ricqlès *et al.* (2008) that rapid growth rates during at least early and mid-ontogeny was plesiomorphic for archosauriforms. The presence of uninterrupted FLB until sub-adult and the sustained high growth rate indicate that *Proterosuchus* was endothermic. Klein *et al.* (2017) put *Proterosuchus* in their Category 2 (growth with FLB early in ontogeny, which then switches to LZB). Additional support for tachymetabolic endothermy is provided by the quantitative analysis of osteohistology across a range of extinct taxa by Cubo & Jalil (2019) who inferred a resting metabolic rate for *Proterosuchus fergusi* within the range of variation of extant birds. *Proterosuchus* and its archosauriform relatives, *Chanaresuchus*, and *Garjainia* also have large femoral nutrient foramina, consistent with tachymetabolic endothermy (Seymour *et al.,*, 2019). (Proxies 1, 3).

***Erythrosuchus*** **—** (Early Triassic). A predatory archosauriform, presumed terrestrial, up to 5 m long and 2 m tall, reconstructed as a heavily built animal with a large head, sharp teeth and an erect stance. Osteohistology in *Erythrosuchus* has attracted several studies of limb bones, ribs and a metatarsal, and there is consistency among them regarding the deposition of highly vascularised FLB throughout ontogeny to at least sub-adulthood (de Ricqlès, Padian & Horner, 2003; de Ricqlès *et al.,* 2008; Botha-Brink & Smith, 2011). De Ricqlès *et al.* (2003) noted the similarity of the observed fibrolamellar pattern to that seen in large mammals, large birds and large dinosaurs. Klein *et al.* (2017) listed *Erythrosuchus* in their Category 3 (fast growth with FLB over a longer period). Additionally, Seymour (in press) identified *Eythrosuchus* as endothermic on the basis of the H–H distance, requiring a four-chambered heart and endotherm-like blood pressure. The evidence is strong that *Erythrosuchus* was a tachymetabolic endotherm (Proxies 1, 2).

***Euparkeria* —** (Middle Triassic). This archosauriform was small (60 cm) and lizard-like with hind legs somewhat longer than the forelegs, raising speculation about possible bipedality and cursorial locomotion. De Ricqlès *et al.* (2008) reported “ill-defined” FLB in a long bone and rib fragments (p. 65), better defined in the cortex, with distinct secondary osteons. They noted a weakly developed three-dimensional network of vascular channels and a similarity to the bone structure seen in small ornithischian dinosaurs such as *Orodromeus*, but stated it was otherwise “not very dinosaur like” (p. 71). By contrast, Botha-Brink & Smith (2011) found only slow and cyclical growth rates, but they had little material to work with. Noting that *Euparkeria* is thought to be the closest known relative to the archosaur crown group, which has been confirmed recently by Sookias (2016), Legendre *et al.* (2013) sought to establish its thermometabolic status. On the basis of a methodology developed by Cubo *et al.* (2012) they concluded that “*Euparkeria* shared with other non-archosaurian archosauromorphs (*Prolacerta*, *Proterosuchus*, and *Erythrosuchus*) a condition of high growth rate compatible with endothermy.” (p. 1343). Klein *et al.* (2017) included *Euparkeria* in their Category 2 (growth with FLB early in ontogeny, which then switches to LZB). Eigenvector mapping led Legendre *et al.* (2016) and Cubo & Jalil (2019) to conclude that the metabolic rate of *Euparkeria* falls within the range of extant mammals. (Proxy 1)

**Phytosauria** (Late Triassic). Phytosaurs were large (2–12 m, most 2–3 m), slender-snouted, well armoured, semi-aquatic, carnivorous reptiles similar in appearance to modern crocodylians and possibly occupying similar habitats. They may have been a sister group to Archosauria (Nesbitt, 2011). De Ricqlès *et al.* (2003) examined two phytosaur femurs, one from *Rutiodon* and one indeterminate. They reported well-vascularised FLB in the inner cortex and parallel-fibred bone externally in both samples. Growth slowed down as ontogeny developed, with LAGs in the outer, parallel layers of both specimens suggesting small annual growth increments. They concluded (p. 97) that phytosaurs show “typical histological patterns seen in the long bones of living crocodiles and evidently grew much like them. Certainly they grew much more like crocodiles than like ornithosuchians, …which seem to have grown at sustained higher rates”. Klein *et al.* (2017) included phytosaurs in their Category 2 (growth with FLB early in ontogeny which then switches to LZB) giving *Rutiodon* as an example. Scheyer, Desojo & Cerda (2014) examined osteoderms from five phytosaurian taxa (two of uncertain identity) and found FLB and woven bone in some, but not all, of them and a small amount of Haversian bone in one. They interpreted their findings as evidence of higher bone growth rates. A ‘new’ marine phytosaur, *Mystrisuchus steinbergeri*, was recently named and its histology described. Tibial microstructure revealed a cortex made of lamellar-zonal bone, eight growth marks, and no indication that adult size had been attained (R.J. Butler *et al.,* 2019). There is little or no evidence to imply tachymetabolism, but the conclusion by de Ricqlès *et al.* (2003), who saw histological patterns reminiscent of modern crocodylians suggests that phytosaurs may have undergone an evolution parallelling that described for modern crocodylians (Seymour *et al.,* 2004). Perhaps, like modern crocodylians, in adapting to a semi-aquatic lifestyle their ancestral capacity for tachymetabolic endothermy was no longer expressed. Legendre *et al.* (2016) came to the same conclusion and phytosaurs are identified as ectotherms in Fig. 4 of the main paper. (Proxy 1; criterion for endothermy not satisfied).

**Archosauria (Pseudosuchia/Crurotarsi)**

***Ornithosuchus* (Pseudosuchia) —** (Late Triassic). This was a large (up to at least 4 m), facultatively bipedal carnivore. It meets the physiological criteria for tachymetabolic endothermy on the basis of bipedality and body size (Pontzer, Allen & Hutchinson, 2009) and H–H distance (Seymour, in press). (Proxies 2, 4)

***Gracilisuchus* (Suchia)** **—** (Mid-Triassic). This small (up to 30 cm) gracile carnivore had hind legs longer than forelegs and was at least a facultative, if not an obligate, biped. Although too small to qualify for endothermy by the Pontzer *et al.* (2009) criterion, Seymour (in press) notes that no living ectotherm is primarily bipedal. Also, its habitus suggests an active, predatory lifestyle and endothermy was common among other early pseudosuchians. So *Gracilisuchus* was very likely to have been a tachymetabolic endotherm. However, without more compelling evidence, perhaps osteohistological, its metabolic status remains uncertain and it is shown in Fig. 4 with a question mark. (Proxy 4? combined with active, predatory lifestyle and phylogenetic bridging).

**Aetosauria (Suchia) —** (Late Triassic). Aetosaurs were large (3–7 m), stocky, quadrupedal, erect, heavily armoured, herbivorous pseudosuchians with a small head in relation to the body and an upturned snout. About 25 genera are recognised, all within the family Stagonolepidae. High rates of growth early in ontogeny have been reported in a number of genera, based on osteohistological studies of long bones and, mostly, osteoderms (de Ricqlès *et al.,* 2003; Scheyer *et al.,* 2014; Hoffman, Heckert & Zanno, 2018). High growth rates were not, however, typical across all species, implying physiological diversity (Taborda, Cerda & Desojo, 2013; Scheyer *et al.,* 2014; Hoffman *et al.,* 2018). High rates were judged on the presence of vascularised FLB and, in some cases, Haversian systems as well. However, de Ricqles *et al.* (2003) from work on long bones, noted similarities more closely aligned with extant crocodylians and markedly different from the situation in pterosaurs and dinosaurs. Klein *et al.* (2017) judged aetosaurs as their Category 2, presumably on the basis of occurrences of well-vascularised FLB and high growth rate early in ontogeny. Although the osteological evidence is inconclusive, in the very large *Desmatosuchus* (4.5 m) with hind legs much longer than forelegs, and reconstructed with its hips well above head level, a large H–H distance as discussed by Seymour (1976, 2016) would have dictated the need for a four-chambered heart and endotherm-like blood pressure. (Proxies 1, 2)

**Poposauroidea (Paracrocodylomorpha) —** (Early–Late Triassic). The best-known genera are *Poposaurus* (4 m, 60–75 kg) and *Effigia* (2 m), both thought to have been swift bipedal predators, convergent in some respects with theropod dinosaurs, whose attributes would have been required for the ‘theropod-like’ lifestyle inferred by Gauthier *et al.* (2011). Likewise, the biomechanics study by Pontzer *et al.* (2009) implies that bipedal running by an animal of this size would need to be tachymetabolic. Consistent with tachymetabolism, Schachner (2010) identified the likelihood of a respiratory system capable of unidirectional airflow, similar to that of birds or, at least a competent system such as a ‘cuirassal basket’ breathing mechanism as suggested by Carrier & Farmer (2000*a*,*b*) and Schachner *et al.* (2011) for basal archosaurs, or the gastralial aspiration pump proposed by Claessens (2004). The experimental demonstration of unidirectional airflow in the lungs of extant crocodylians (Farmer & Sanders, 2010), which are thought to have had endothermic ancestry (Seymour *et al.,* 2004), lends further support to the possibility that poposauroids had unidirectional airflow in their lungs, like birds. From the criteria developed by Pontzer *et al.* (2009), *Effigia* too, standing at 2 m, would likely have needed tachymetabolism to support swift bipedal locomotion. There seem to be no osteohistological data for *Poposaurus*, but Nesbitt (2007) reported extensive FLB and Haversian systems in a right femur of *Effigia*, implying high growth rate, and on that basis alone Klein *et al.* (2017) recorded *Effigia* in their Category 3 (fast growth with FLB over a long period). Additionally, on the basis of their vertical H–H distance, Seymour (in press) identified *Poposaurus*, *Effigia*, and four other poposauroids (*Arizonasaurus*, *Lotosaurus*, *Sillosuchus*, *Ctenosauriscus*) as requiring a four-chambered heart and endotherm-like blood pressure (Fig. 3). Femoral nutrient foramen size in the poposauroid *Lotosaurus* is higher than predicted by modern reptiles (Seymour *et al.,* 2019). All evidence points strongly to poposauroids having been tachymetabolic endotherms. (Proxies 1, 2, 3, 4 as well as evidence for a ‘bird-like’ respiratory system).

***Batrachotomus* (Loricata) —** (Middle Triassic). A large (up to 6 m), heavily built predatory quadruped, with the hind legs much longer than the forelegs and an erect stance. A femur examined by Klein *et al.* (2017) showed FLB with numerous round osteocyte lacunae and primary osteons, with growth cycles suggesting that the animal had grown to an estimated length of 5.6 m in about four years. No Haversian systems were found, but the authors noted structures that could be interpreted as secondary osteons. Vascular density within the FLB was higher than in *Effigia* and *Postosuchus*, both judged herein to have been tachymetabolic endotherms. It fitted their Category 3 (fast growth with FLB over a longer period). It was so large, that the H–H distance would have been in the endothermic range, particularly if it reared on its hind legs (Seymour, 1976, 2016). *Batrachotomus* is highly likely to have been a tachymetabolic endotherm (Proxies 1, 2).

***Postosuchus* (Rauisuchidae) —** (Late Triassic). A heavily built, predatory rauisuchian with an erect stance, 3.5–6 m in length, and with hind legs longer than forelegs. Probably bipedal (Weinbaum, 2013), but even if quadrupedal they would almost certainly be capable of facultative bipedalism, and so endothermy is likely on the basis of their size as bipeds (Pontzer *et al.,* 2009) and H–H distance (Seymour, 1976, 2016). de Ricqlès *et al.* (2003) reported vascularised FLB and Legendre *et al.* (2013) considered its growth rate consistent with endothermy. *Postosuchus* was very likely a tachymetabolic endotherm on the basis of bone histology and growth rate, body size, posture, and an inferred capacity for predation on dicynodonts (early therapsids). Klein *et al.* (2017) placed *Postosuchus* in their Category 3 (fast growth with FLB over a longer period). From their quantitative analysis of osteohistology across a range of extinct taxa, Cubo & Jalil (2019) inferred a resting metabolic rate for *Postosuchus kirkpatricki* within the range of variation of extant birds, i.e. tachymetabolic endothermy. (Proxies 1, 2 and 4).

***Terrestrisuchus* (non-crocodyliform crocodylomorpha) —** (Late Triassic). Possibly a juvenile *Saltoposuchus* (Allen, 2003), also a crocodylomorph, *Terrestrisuchus* is reconstructed as almost greyhound-like in shape and size, with long legs directly under the body and clearly well-suited for fast running. Its very long tail suggests agility and would have helped with balance as it chased small prey. It grew to about 1 m long and weighed 15 kg. Longer hind legs imply a possibility for bipedality, a capacity which would depend upon tachymetabolism in full-grown individuals (Pontzer *et al.,* 2009) and that capacity is supported by evidence for continuous growth inferred from well-vascularised FLB in long bones (de Ricqlès *et al.,* 2003). Padian, Horner & de Ricqlès *et al.* (2004) noted a simple form of FLB and other histological features that suggested that growth would have been rapid for a pseudosuchian and mostly continuous. Klein *et al.* (2017) assigned them to Category 3 (fast growth with FLB over a long period). A conclusion of tachymetabolic endothermy is indicated (Proxies 1, 4).

**Archosauria (Ornithodira/Avemetatarsalia)**

**Pterosauria** (Late Triassic–Late Cretaceous). Writing when scepticism about endothermy in extinct sauropsids was higher than it is now, Padian (1983) made a careful analysis of pterosaur skeletal anatomy, referring to *Rhamphorhynchus*, *Pterodactylus*, *Pteranodon* and others and concluded that they were active flyers rather than gliders, and resembled birds morphologically in having pneumatic foramina in many postcranial bones. Accordingly, he suggested (p. 237) they were “likely to have been as endothermic as any Mesozoic archosaur, including birds”. This was followed by osteological studies of numerous genera, including *Pteranodon*, *Dimorphodon*, *Ornithocheirus*, *Pterodaustro*, *Rhamphorhynchus* and *Quetzalcoatlus*, which found abundant limb bone FLB, providing evidence for rapid growth, and mostly persisting to sub-adult sizes at least (Bennett, 1993; de Ricqlès *et al.,* 2000; Padian *et al.,* 2004; Chinsamy, Codorniú & Chiappe, 2008; Steel, 2008). In summarising their results, de Ricqlès *et al.* (2000) observed that the bone tissues suggest that pterosaurs had generally high growth rates, high metabolic rates, and resemble those of birds more closely than those of crocodiles or lizards. In a totally different approach, Claessens, O’Connor & Unwin (2009) undertook a detailed modern morphological analysis of skeletal material from both basal (*Eudimorphodon* and *Rhamphorhynchus*) and derived (*Pteranodon* and *Anhanguera*) pterosaurs. They concluded that pterosaurs had a highly effective flow-through respiratory system, capable of providing the respiratory and metabolic requirements of sustaining powered flight. The largest pterosaur *Quetzalcoatlus* was as tall as a giraffe, so the mean arterial blood pressure would have exceeded 200 mm Hg (26.7 kPa), clearly in the endothermic range (Seymour, 1976, 2016). Furthermore, Jenkins & Pratson (2005) reported in a conference abstract the results of their bioenergetic analysis of pterosaur flight capability, which showed that “large pterosaurs may have been endothermic and therefore more metabolically similar to birds than to reptiles” (p. 1). Putting this evidence together with the increasingly accepted view that endothermy was plesiomorphic within archosauria (Legendre *et al.,* 2016; Cubo & Jalil, 2019), there is strong evidence for tachymetabolic endothermy being a basal characteristic of Pterosauria. (Proxies 1, 2, plus a bird-like respiratory system and a bioenergetic analysis consistent with meeting requirements for flight).

**Dinosauriformes**

***Lewisuchus*** — (Middle Triassic). A small (length 1 m), possibly erect bipedal early Mid-Triassic dinosauriform. Marsà, Agnolín & Novas (2019) reported densely vascularised longitudinal, laminar, and reticular FLB in the femur and tibia. They argued that the fast growth of *L. admixtus* implies a relatively high metabolic rate, similar to that of basal dinosaurs. Furthermore, the Triassic dinosauriformes *Lewisuchus*, *Asilisaurus* and *Tawa* (as well as the early archosaurs *Teleocrater* and *Dromomeron*, a dinosauromorph) all have large femoral nutrient foramina consistent with tachymetabolism (Seymour *et al.,* 2019). Tachymetabolic endothermy is indicated (Proxies 1, 3).

**Dinosauria**

**Ornithischia**

**Heterodontosauridae —** (Early Jurassic–Early Cretaceous). This family comprises at least half a dozen genera of small, possibly very early ornithischian dinosaurs over a size range of about 2 kg (*[Tianyulong](https://en.wikipedia.org/wiki/Tianyulong)*) to 10 kg (*Heterodontosaurus*), grouped mainly according to unusual dentary characteristics, as suggested by the family name. They were bipedal and cursorial. Becerra *et al.* (2016) examined the histology of metatarsal bones among fragmentary remains of an unidentifiable heterodontosaurid (possibly *Manidens condorensis*) and reported FLB. [*Tianyulong*](https://en.wikipedia.org/wiki/Tianyulong) is reported to have been covered with integumentary filaments (Zheng *et al.,* 2009), and if this is a correct interpretation then speculation as to their function logically includes thermoregulation. In their study of the metabolic requirements for bipedality, Pontzer *et al.* (2009) calculated that a 10 kg bipedal *Heterodontodosaurus* would have “exceeded ectothermic capabilities ……. at moderate running speeds” (p. 2). Histological examinations were performed recently on two wallaby-sized ‘hypsilophodontids’ (*sensu* ‘small-bodied ornithischians possessing basal ornithopod characteristics’), whose relationships are quite uncertain, that would have lived within the Cretaceous Antarctic Circle (Woodward *et al.,* 2011; Woodward, Rich & Vickers-Rich, 2018). The authors reported well-vascularised FLB early in life, with growth slowing as their small adult size was attained. The authors also remarked at the similarities between the bone tissue microstructures of these ‘polar’ dinosaurs and their lower-latitude relatives, suggesting they were physiologically pre-adapted to endure the freezing polar temperatures and winter darkness. The femoral nutrient foramen of an undescribed hypsilophodont from Canada is larger than predicted by living mammals, indicating a higher metabolic rate (Seymour *et al.*, 2012). Taken collectively, the evidence supports tachymetabolic endothermy in small basal ornithodontids including Heterodontosauridae (Proxies 1, 3, 4).

**Thyreophora**

***Lesothosaurus* —** (Early Jurassic). *Lesothosaurus* was a basal thyreophoran (Butler, Upchurch & Norman, 2008), interpreted by Thulborn (1972) as a small and agile biped with distinct cursorial ability. Growing to 2 m, it would have weighed more than the 10 kg *Heterodontosaurus* considered by Pontzer *et al.* (2009) and, thus, would likely have required more energy for its locomotion than could have been supplied by bradymetabolism. Indeed, these authors judged that moderate running by a 5 kg *Lesothosaurus* would have required a metabolic intensity characteristic of endothermy. Histology of its long bones revealed FLB, implying a fast growth rate (Knoll, Padian & Ricqlès, 2010). These two lines of evidence suggest that *Lesothosaurus* was a tachymetabolic endotherm. Confirmation was provided by the quantitative analysis of osteohistology across a range of extinct taxa by Cubo & Jalil (2019), who inferred a resting metabolic rate for *Lesothosaurus diagnosticus* within the range of variation observed in extant birds. (Proxies 1, 4)

**Ankylosauria —** (Mid Jurassic–Late Cretaceous). Anklyosaurs were large (up to 8–10 m), bulky, heavily armoured herbivorous quadrupeds with an erect stance, some growing to a mass of several tonnes. Long bone histology and growth patterns were studied by Stein, Hayashi & Sander (2013), who examined several specimens of an ‘indeterminate’ ankylosaurid, and several specimens of the sister family Nodosauridae (*Hungarosaurus*, *Edmontonia* and one ‘indeterminate’). They found ‘atypical’ FLB in most samples, a mixture of woven and parallel-fibred tissue, which they interpreted as evidence of “relatively rapid growth” (p. 11) early in ontogeny, slowing as adulthood was approached, but slower than in other dinosaurs. Noting the osteology of the more basal thyreophoran *Lesothosaurus* (Knoll *et al.*, 2010), Stein *et al.,* (2013) suggested that fast growth may have been plesiomorphic in Ornithischia, with a slower rate apomorphic in anklosaurs and stegosaurs. A recent study by Cerda *et al.* (2019) investigated the osteohistology of *Antarctopelta* from Antarctica and found abundant well-vascularised cortical FLB and that, like polar hypsilophodontids, the bone histology of this high-latitude taxon differed little from its lower-latitude relatives. Additionally, the H–H distance in the larger ankylosaurs, including *Hungarosaurus*, *Edmontonia* and probably *Antarctopelta* was greater than the minimum criterion for endothermy (Seymour, 1976, 2016). Their osteology and bodily conformation, as well as their ancestry, shows that ankylosaurs (and stegosaurs, see below) were tachymetabolic endotherms, but possibly at a lower metabolic intensity than many other dinosaurs (Proxies 2, possibly 1).

**Stegosauria (Thyreophora, Ornithischia) —** (Middle Jurassic–Early Cretaceous). Stegosaurs were large (4–7 m, some reaching several tonnes), herbivorous, quadrupedal dinosaurs with an erect stance and are distinctive for being armoured by an axial midline ridge of stout plate-like and spiked osteoderms along the back and tail. In the larger ones the H–H distance was greater than the minimum criterion for endothermy (Seymour, 1976, 2016). Well-vascularised FLB has been reported in long bones and osteoderms of *Stegosaurus* (Hayashi, Carpenter & Suzuki, 2009). Redelstorff *et al.* (2013) examined an ontogenetic series of six femora from the more basal *Kentrosaurus* and reported extensive and well-vascularised FLB. *Kentrosaurus* also has large femoral nutrient foramina (Seymour *et al.*, 2012). Collectively, stegosaurs satisfy at least two of our proxies for tachymetabolism, and it is worth noting that this is consistent with the basal thyreophoran *Lesothosaurus* having been judged a tachymetabolic endotherm too (see above) (Proxies 2, 3, possibly 1).

**Neornithischia**

**Ceratopsia (Marginocephalia) —** (Late Jurassic–Late Cretaceous). This is a very diverse grouping of herbivorous ‘horned’ dinosaurs with characteristic skull structure, some quite small, such as the bipedal *Psittacosaurus* (2 m), and some very large, such as the quadrupedal *Achelousaurus horneri* (6 m, 3000 kg). The likelihood that at least some ceratopsians were endothermic accrues from several studies. For example, Erickson & Tumanova (2000) described well-vascularised FLB in the basal ceratopsid *Psittacosaurus mongoliensis*. Padian *et al.* (2004) reported that the bone matrix of *Psittacosaurus* was woven-fibred throughout, “suggesting higher growth rates than in typical reptiles of today, such as crocodiles and lizards” (p. 559). Most recently, the bone histology of *Psittacosaurus lujiatunensis* was described as having FLB throughout growth but attaining a smaller adult body size than *P. mongoliensis* (Zhao *et al.*, 2019). An ontogenetic study of the arctic-ranging *Pachyrhinosaurus* reveals well-vascularised FLB early in life (Erickson & Druckenmiller, 2011), and the authors state that the growth rates were similar to lower-latitude ceratopsids, suggesting “a greater physiological capacity to tolerate these conditions than that of extant ectothermic reptiles” (p. 332). A palaeothermometric study by Barrick, Showers & Fischer (1996) concluded that the quadrupedal *Montanaceratops* (3 m) and a juvenile *Achelousaurus* had a sufficiently stable *T*_b_ over a wide range of climates, compared to ectotherms, to suggest that they were endothermic, probably at an ‘intermediate’ level. In a subsequent palaeothermometric study on the ceratopsian *Montanaceratops* (3 m), Amiot *et al.* (2006) concluded that it maintained “rather constant” *T*_b_, i.e. homeothermy. They attributed this to endothermy rather than gigantothermy on the basis of the narrow range of *T*_b_ values calculated, its similarity to temperatures of modern-day endotherms, and the similarity between low-latitude and high-latitude dinosaurs. *Montanaceratops* was probably too small for gigantothermy to be an issue, and *Psittacosaurus* certainly was, as would be juveniles of all of them. *Centrosaurus*, *Styracosaurus* and *Pachyrhinosaurus* all have large femoral nutrient foramina, exceeding those of similarly sized mammals, indicating endothermic bone perfusion (Seymour *et al.*, 2012). Adding ancestry, body conformation and the H–H distance in an erect posture along with the other findings, tachymetabolic endothermy must have been characteristic in ceratopsians (Proxies 1, 2, 3, 5).

**Pachycephalosauria (Marginocephalia)** **—** (Late Cretaceous). Pachycephalosaurs were herbivorous, bipedal dinosaurs of moderate body size (2–4 m) made famous by having a greatly thickened skull, often interpreted as useful in head-butting contests. Perhaps the best known is *Pachycephalosaurus*, one of the largest, growing to nearly 5 m. They meet the criteria for tachymetabolic endothermy through their body size and habitus. They were obligatory bipeds, comfortably large enough to meet the criteria for endothermy identified by Pontzer *et al.* (2009) and the size and posture of most imply a H–H distance requiring endotherm-equivalent carotid arterial blood pressure and a four-chambered heart (Seymour, 1976, 2016). (Proxies 2, 4)

***Orodromeus*, *Thescelosaurus* (Ornithopoda) —** (Late Cretaceous). In a femur from *Orodromeus*, Padian *et al.* (2004) noted FLB and parallel-fibred bone in the cortex, the latter towards the outer edge. The deep cortex was highly vascularised. They concluded (p. 559) that the “tissue organization of this bone is intermediate in many respects between crocodiles and *Scutellosaurus* on the one hand and *Maiasaura* on the other”. Horner *et al.* (2009) examined femora and tibiae from a series of specimens. They reported that the cortices of the long bones consisted of fibrolamellar, and not lamellar-zonal tissue types, and that vascularity was relatively moderate. The authors concluded that *Orodromeus* grew more rapidly than crocodiles, although not as rapidly as birds, and that growth was most rapid soon after hatching. *Orodromeus* was almost certainly an obligate biped, although too small to meet the Pontzer *et al.* (2009) criterion for obligate tachymetabolism. However, no living ectotherm is an obligate biped, and it is worth noting that its large ‘cousin’ the parkosaurid *Thescelosaurus* (2.5–4 m) was almost certainly endothermic on the basis of both H–H distance (Seymour, 1976, 2016) and bipedality (Pontzer *et al.*, 2009). On balance, there is a high likelihood that both *Orodromeus* and *Thescelosaurus* were tachymetabolic endotherms (Proxies 2, 4, possibly 1).

**Iguanodontia (Ornithopoda) —** (Mid Jurassic–Late Cretaceous). Studies on three differently sized iguanodontians are informative. *Gasparinisaura cincosaltensis* was a small (1.7–2 m), bipedal, herbivorous basal iguanodontian. Cerda & Chinsamy (2012) examined postcranial bones of 11 specimens and found FLB tissue in the cortices of all of them, suggesting rapid osteogenesis and fast growth early on, slowing later in ontogeny, with bone structure becoming more parallel-fibred than woven-fibred as growth ceased.

*Rhabdodon* was a larger (up to 6 m) iguanodontid, also bipedal and herbivorous. Prondvai (2014) made a histological study of three rhabdodontid genera, *Mochlodon*, *Zalmoxes* and *Rhabdodon*, and found FLB in all of them, with *Rhabdodon* showing a faster growth rate than the other two. *Rhabdodon* was included in the oxygen isotope analysis by Amiot *et al.* (2006) and fitted the pattern they reported of “rather constant” *T*_b_, i.e. homeothermy. They attributed this to endothermy rather than gigantothermy because of the narrow range of their calculated *T*_b_ values, its similarity to temperatures of modern-day endotherms, and the similarity between low-latitude and high-latitude dinosaurs. *Tenontosaurus* was an even larger iguanodontid (6.5–8 m, 1,000–2,000 kg), largely quadrupedal but thought to browse by rising onto its hind legs. Werning (2012) studied the long bone histology of *T. tilletti* over an ontogenetic range and found well-vascularised woven bone tissue during early to subadult stages, providing evidence for extremely rapid growth early in life, sustained rapid growth through sub-adult ontogeny, followed by a multi-year period of slow growth as skeletal maturity approached. Paul (2010) calculated that a 1.7 m, *Gasparinisaura* would have weighed 13 kg, so both it and, more certainly, the larger *Rhabdodon*, would have needed to rely on tachymetabolism to support their bipedal locomotion (Pontzer *et al.*, 2009). The H–H distance in both *Rhabdodon* and *Tenontosaurus* also implies a need for tachymetabolism (Seymour, 1976, 2016). Additionally, the large size of femoral nutrient foramina in the iguanodontian *Dysalotosaurus* also implies tachymetabolism (Seymour *et al.*, 2012). These observations imply that tachymetabolic endothermy was characteristic of iguanadontians (Proxies 1, 2, 3, 4, 5).

**Hadrosauria (Ornithopoda) —** (Early–Late Cretaceous). The osteohistology of hadrosaurs has been studied extensively. Genera from each of the two major groups of hadrosaurs (Lambeosaurinae and Saurolophinae) may be representative. *Hypacrosaurus stebingeri* (9 m, 4,000 kg) was a large herbivorous bipedal/quadrupedal ‘duckbill’ lambeosaurine with hind legs longer than forelegs. Its size and a capacity for erect posture standing on hind legs imply tachymetabolic endothermy because of the H–H distance (Seymour, 1976, 2016), and also consistent with a need for tachymetabolic support of the locomotory cost of its bipedality (Pontzer *et al.*, 2009). Furthermore Barrick *et al.* (1996) reported evidence of stable *T*_b_ (homeothermy) in adults and juveniles, the latter showing that their thermal stability was not the result of gigantothermy. *Maiasaura peeblesorum* (Saurolophinae) (7 m, 2,300 kg) was a hadrosaurine with hind legs longer than forelegs, broadly similar to *Hypacrosaurus*. In adulthood they were more quadrupedal than bipedal. The size, body form, and H–H distance when erect imply tachymetabolic endothermy. This conclusion is consistent with bone histology that implies tachymetabolism (FLB and Haversian systems) and maximum growth rates similar to those observed in extant large-bodied mammals and birds (Horner, de Ricqlès & Padian, 2000) as well as high sustained growth rates occurring over a period of years (Woodward *et al.*, 2015). Endothermy in *M. peeblesorum* is further supported by a recent palaeothermometric study calculating a maternal *T*_b_ of 44 °C ± 2 °C using clumped isotope methodology to assess maternal *T*_b_ from eggshells (Dawson *et al.*, 2020) and also large femoral nutrient foramina consistent with endothermy of unidentified hadrosaurs from Alberta, Canada (Seymour *et al.*, 2012). Hadrosaurs can be considered to have been tachymetabolic endotherms (Proxies 1, 2, 3, 4, 5).

**Dinosauria**

**Saurischia**

***Eoraptor*** — (Late Triassic). This small (up to 1 m, up to 10 kg) long-legged, lightly built bipedal, carnivorous predator was an obligatory biped, similar to extant birds and in contrast to all extant ectotherms. A biomechanical study by Pontzer *et al.* (2009) suggested that a running 10 kg *Heterodontosaurus* would have had a locomotory metabolic requirement beyond what could be supplied by ectothermy, and that it was ‘likely’ an endotherm. By analogy, *Eoraptor* too is likely to have been endothermic, and Seymour (in press) notes that no living ectotherm is primarily bipedal. The body form and interpretations of lifestyle are coherent with endothermy, and there are skeletal similarities with herrerosaurids such as a flexible joint in the lower jaw and a raking manus (Sereno, 1997). It is likely that they were tachymetabolic endotherms (close to satisfying Proxy 4, combined with body form, a highly active lifestyle and possible phylogenetic bridging). However, without further data, particularly from osteohistology, this remains uncertain and so, as for *Gracilisuchus*, we show *Eoraptor* in Fig. 4 with a question mark (Proxy 4? combined with active, predatory lifestyle and phylogenetic bridging).

**Herrerosauridae —** (Late Triassic). Large (4–6 m, up to 150–350 kg), lightly built, bipedal carnivorous early dinosaurs. Endothermy is highly likely, based on the occurrence of well-vascularised FLB in *Herrerasaurus* (de Ricqlès *et al.*, 2003), bipedal posture, and hypothesised pursuit-predator niche. Hypothesised endothermy is also supported by the conclusions of Pontzer *et al.* (2009), who calculated that the locomotory metabolic requirements of swift-running bipedal sauropsids in this body mass range would have needed a metabolic intensity characteristic of endothermy. Their tachymetabolic endothermy is supported also on the basis of H–H vertical distance (Seymour, 1976, 2016), certainly in the larger species. (Proxies 2, 4)

**Prosauropoda —** (Late Triassic). The herbivorous and bipedal sauropodomorph *Plateosaurus* grew to 5–10 m. On the basis of its H–H distance (Seymour, 1976, 2016) and its metabolic requirements for bipedal locomotion (Pontzer *et al.*, 2009), it would have been endothermic and this conclusion is supported by its high growth rate as deduced from extensive long bone FLB (Sander & Klein, 2005; Hofmann & Sander, 2014). Additionally, the femoral nutrient foramen of *Plateosaurus* is larger than predicted by living mammals, indicating higher intensity of locomotion and endothermic metabolic rate (Seymour *et al.*, 2012). On the basis of satisfying at least three of our proxies, tachymetabolic endothermy was characteristic of prosauropods (Proxies 2, 3, 4 and possibly 1).

**Sauropoda —** (Late Triassic–Late Cretaceous). There are multiple lines of evidence that tachymetabolic endothermy was widespread among sauropods. Their high growth rates, evidenced by well-vascularised FLB (Rimblot-Baly, de Ricqlès & Zylberberg, 1995; Sander *et al.*, 2011; Schroeter, Boles & Lacovara, 2011; Lacovara *et al.*, 2014; Stein & Prondvai, 2014; Apaldetti *et al.*, 2018; Curry Rogers & Kulik, 2018), their avian-style respiratory system as revealed by extensive vertebral air sacs (Wedel, 2003*a*,*b*, 2005) and a warm and stable *T*_b_ shown by palaeothermometry (Amiot *et al.*, 2006) imply endothermy across a diversity of species. This conclusion is supported by a vertical H–H distance greater than 3 m, which would have required an arterial blood pressure (>230 mm Hg, 30.7 kPa) well above the endothermic range, even without raising the neck, which turns out to be anatomically and physiologically impossible (Seymour, 2016). Clumped isotope thermometry studies of teeth and eggshells from *Brachiosaurus* and teeth from *Camarasaurus* yielded values of *T*_b_ of approximately 32–38 °C (Eagle *et al.*, 2011, 2015). Some authors have proposed ectothermy for sauropods, with a high and stable *T*_b_ *via* gigantothermy (e.g. Paladino, Spotila & Dodson, 1997). However, bradymetabolic gigantothermy is not possible in juvenile life stages, is vastly energetically inferior to tachymetabolic endothermy (Seymour, 2013) and could not explain the FLB-implied high growth rate of sauropods. Nor could it explain the size of the femoral nutrient foramen of *Giraffatitan brancai*, which lies within the extrapolated range predicted by living mammals, indicating high intensity of locomotion and tachymetabolic endothermy (Seymour *et al.*, 2012). (Proxies 1, 2, 3, 5 and avian-style respiratory system).

**Theropoda.** There are now enough data on Theropoda and Aves to accept that the occurrence of widespread whole-body endothermy among them is sufficiently well demonstrated and there is no need to mount a detailed case here; see reviews by Legendre *et al.* (2016) and Bailleul, O’Connor & Schweitzer (2019). Theropod tachymetabolic endothermy is revealed by numerous osteohistological studies, cardiovascular implications from the size and stature of many, and a bipedal gait in the larger ones (Proxies 1, 2, 4).

**SYNAPSIDA**

We provide below the results of our survey of ‘Pelycosaurs’ and Therapsids with the exception of Mammaliaformes.

**‘Pelycosaurs’**

The informal, non-cladistic name ‘Pelycosaur’ is often used to refer to five categories of so-called ‘mammal-like reptiles’ comprising ‘all synapsids not held to be within the clade Therapsida’, that is, the Caseasauria, Varanopidae (maybe not a synapsid, see below), Ophiacodontidae, Edaphosauridae and Sphenacodontia. Each arose in the Late Carboniferous and none survived past the mid-Permian, unless the Therapsida arose from the Sphenacodontia as suggested by Benson (2012). There have been comparatively few osteohistological studies of ‘pelycosaurs’ and few with possible endothermy as a focus.

**Caseasauria —** (Late Carboniferous–Middle Permian). Two families comprise the Caseasauria, the Eothyrididae and the Caseidae and they are rather different from each other*.* The former is thought to have been insectivorous and more primitive, the latter mostly herbivorous, much larger, and for which some species at least were terrestrial and many may have been aquatic. In a substantial study of the osteohistology of the long bones of several Caseidae (*Ennatosaurus*, *Alierasaurus*, *Ruthenosaurus* and *Cotylosaurus*), Shelton (2014) found no evidence of well-vascularised FLB or other indications of high growth rate.

**Varanopidae —** (Late Carboniferous–Middle Permian). The classification of varanopids as synapsids has been questioned by Macdougall *et al.* (2018) and Ford & Benson (2020) found them to be a better fit within Diapsida. They were small, monitor lizard-like agile and carnivorous terrestrial predators and ranged from 1 to 2 m in length. In osteohistological studies of *Varanops* and *Mycterosaurus*, Shelton (2014) and Huttenlocker & Shelton (2020) found no evidence of fast growth rates, and neither does anything about their body size or habitus suggest tachymetabolic endothermy.

***Ophiacodontidae* —** (Late Carboniferous–Early Permian). Shelton & Sander (2017) examined post-cranial skeletal material from several species of *Ophiacodon*, large (1.6–3 m, 26–230 kg) quadrupedal basal synapsids. The authors described classic characteristics of ‘true’ FLB with the cortex comprising “primary osteons in a woven bone matrix (that) remains highly vascularized throughout ontogeny, providing evidence for fast skeletal growth” (p. 397). This evidence of well-vascularised FLB sustained throughout ontogeny is compelling. They concluded (p. 397) that “*Ophiacodon* is more advanced or ‘mammal-like’ in terms of the osteonal development, bone matrix, and skeletal growth than what has been described thus far for any other pelycosaur taxon”. This is in agreement with the much earlier findings of Enlow & Brown (1957) whose osteohistological work convinced them that mammalian endothermy arose in the Carboniferous. In a subsequent osteohistological study, however, on a different ophiacodontid, *Clepsydrops collettii* (Late Carboniferous), Laurin & de Buffrénil (2016) found no FLB and described ‘woven-like’ bone, implying fast growth early on, then slowing, with similarity to that of varanid lizards. More recently still, using phylogenetic eigenvector mapping to infer the metabolic rate of a range of non-mammalian synapsids, Faure-Brac & Cubo (2020) also reported an estimate of metabolic rate for *Clepsydrops* indicative of ectothermy. In the same study, these authors concluded that *Ophiacodon uniformis* also was ectothermic, together with *Dimetrodon*, *Sphenacodon* and an edaphosaur, all of which we judge to be tachymetabolic endotherms on the basis of osteological and cardiovascular evidence (see below). It is worth noting, however, that among these ‘pelycosaurs’ the metabolic rate Faure-Brac & Cubo (2020) calculated for *Ophiacodon uniformis* did approach the threshold for endothermy. Additionally, although Knaus *et al.* (2021) did not include their four species of *Ophiacodon* among their list of non-mammalian synapsids with an elevated maximum metabolic rate (MMR) consistent with endothermy, the MMR calculations for *Ophiacodon* based on eigenvector mapping did cluster well with edaphosaurs, sphenacodontids, dicynodonts and gorgonopsids. On balance, the FLB evidence for tachymetabolic endothermy presented by Shelton & Sander (2017) is strong, and in the studies by both Faure-Brac & Cubo (2020) and Knaus *et al.* (2021) *Ophiacodon* came very close to meeting the criteria for endothermy. The issue is not resolved but we judge that *Ophiacodon* was most likely characterised by tachymetabolic endothermy (Proxy 1, likely also 3).

**Sphenacodontia** (Late Carboniferous–Early Permian). Using their innovative osteological approach (see above), Faure-Brac & Cubo (2020) reported estimates of metabolic rate in *Dimetrodon* sp*.* and *Sphenacodon* sp. as indicative of ectothermy. However, three studies imply that at least some sphenacodontids were endothermic. Huttenlocker, Rega & Sumida (2010) examined the hyperextended spines of *Dimetrodon* and found that, as well as lamellar bone, each exhibited extensive well-vascularised parallel-fibred and FLB, implying rapid outgrowth of the spines. Shelton *et al.* (2012) found what they called incipient FLB (IFLB) in the postcranial skeleton of *Dimetrodon natalis* throughout ontogeny. The IFLB comprised highly vascularised woven and parallel-fibred bone in combination, coupled with incipient primary osteons. They suggested a metabolic rate slightly higher than that in modern reptiles. These studies may be indicative of endothermy, but they are not by themselves convincing. However, Seymour (2016) noted that the top of the neural spine of *Dimetrodon grandis* reached 1.5 m above the heart (Fig. 3), implying a MAP of 115 mm Hg (15.33 kPa), which is well within the endothermic range and requires a four-chambered heart. More recently it was observed that the nutrient foramina from several species of *Dimetrodon* indicated elevated metabolic rates consistent with endothermy (Knaus *et al*., 2021). Although *Sphenacodon* lacked a tall sail and has been assessed as ectothermic, it seems clear that *Dimetrodon* at least among sphenacodontids was a tachymetabolic endotherm (Proxies 2, 3).

**Edaphosauria** (Late Carboniferous–Early Permian). Using phylogenetic eigenvector

mapping, Faure-Brac & Cubo (2020) reported an estimate of metabolic rate in *Edaphosaurus boanerges* that is indicative of ectothermy. Huttenlocker, Mazierski & Reisz (2011) reported FLB in the lateral tubercles of the spines of edaphosaurs but made no interpretation about the animals’ metabolic rate. As in *Dimetrodon*, the elevation of the top of the tallest neural spines of *Edaphosaurus* implies endotherm-like blood pressure (Seymour, 1976, 2016), so large edaphosaurs at least must have been tachymetabolic endotherms. Additionally, nutrient foramina from edaphosaurs indicate elevated metabolic rates consistent with endothermy (Knaus *et al*., 2021). The weight of evidence thus favours tachymetabolic endothermy (Proxies 2, 3).

**Therapsida**

The case for tachymetabolic endothermy in numerous post-Triassic therapsids is now well established, as the review by Benton (2020) shows, and our literature survey confirms this and expands its taxonomic reach (Fig. 4). Benton noted its “emergence” in the Late Permian in both sauropsids and therapsids, and “acceleration” in the Early Triassic after the PTME. Noting the loss of so many of the Permian endotherms to the PTME, he thus considered endothermy to have its origins in the Triassic, and contemporaneously in both sauropsids and synapsids. But in sauropsids, as discussed above, whole-body endothermy is considered basal to Archosauromorpha from the Middle Permian. And among therapsids, whereas the Dinocephalia and Gorgonopsia arose in the mid-Permian and did not extend to the Triassic, the Dicynodontia, Therocephalia and Cynodontia all spanned the PTME and in each of these there is evidence of endothermy both before and after the extinction event. The results for all therapsids are shown graphically in Fig. 4 and those for the pre-PTME are reported in the main text. The complete explanatory details for all therapsids included in our survey (Dinocephalia, Gorgonopsia, Dicynodontia, Therocephalia, Cynodontia, Cynognathia and Probainognathia) are provided below.

**Dinocephalia —** (Middle–Late Permian). The only evidence we have for this group is that at least one member, *Moschops*, achieved a large stature and a H-H distance of about 89 cm thus requiring a MAP of approximately 120 mm Hg (16 kPa) to perfuse the brain, clearly in the endothermic range (Fig. 3). (Proxy 2).

**Gorgonopsia —** (Middle–Late Permian). Long bone histology of these bear-sized, quadrupedal carnivores, judged to be active predators on the basis of their striking sabre-like canines, was first studied by de Ricqlès (1969, cited in Chinsamy-Turan & Ray, 2012). He reported FLB with some development of Haversian systems in four genera of gorgonopsians (*Aelurognathus*, Late Permian; *Scymnognathus*, now *Gorgonops*, Late Permian; *Dixeya*, now *Aelurognathus*; *Lycaenops*, Middle Permian; and one indeterminate). Ray, Botha & Chinsamy (2004) reported FLB from an additional genus, *Scylacops* (Late Permian) and found the cortices to contain wide zones of FLB, interrupted by LAGs and annuli comprising avascular lamellar bone, suggesting rapid growth interrupted periodically. In contrast to *Scylacops*, they reported that *Aelurognathus* lacked these interruptions, suggesting its rapid growth was sustained. The osteohistology of gorgonopsians supports tachymetabolic endothermy and this is supported by reconstructions of them as active predators carrying themselves erect. The largest gorgonopsian *Inostrancevia* was quadrupedal, yet has a H–H distance consistent with endothermy (Fig. 3). Additionally, nutrient foramina from gorgonopsians indicate elevated metabolic rates consistent with endothermy (Knaus *et al*., 2021). There is thus good evidence that tachymetabolic endothermy was widespread in gorgonopsians (Proxies 1, 2, 3).

**Dicynodontia —** (Middle–Late Permian–Triassic). Most studies of this long-lived group of sturdy quadrupedal herbivores report bone histology implying high growth rates, i.e. FLB and Haversian remodelling, particularly during their growth phase and to varying degrees: *Lystrosaurus* (Late Permian–Early Triassic), *Oudenodon* (Late Permian), *Moghreberia* (Late Triassic), *Placerias* (Triassic), *Wadiasaurus* (Middle Triassic), and *Tropidostoma* (Late Permian) (Ray *et al*., 2004; Ray, Chinsamy & Bandyopadhyay, 2005; Botha & Angielczyk, 2007; Ray, Bandyopadhyay & Bhawal, 2009; Botha-Brink & Angielczyk, 2010; Green, Schweitzer & Lamm, 2010). These animals covered a size range from 0.5 m to the elephant-sized, 4.5 m long *Lisowicia* (Late Triassic), weighing more than 8000 kg (Sulej & Niedźwiedzki, 2019). The bone histology of *Lisowicia* reflects uninterrupted fast growth, with a highly remodelled inner cortex. Olivier *et al.* (2017) used analyses of osteohistological attributes combined with phylogenetic eigenvector mapping to infer resting metabolic rates of three fossil synapsids, *Moghreberia*, *Lystrosaurus* and *Oudenodon*, and reported metabolic rates within the range for extant mammals and disjunct from a series of extant ectotherms. Additionally, Whitney & Sidor (2020) deduced from daily growth rings in tusk dentine that polar *Lystrosaurus* showed periods of seasonal torpor, whereas in non-polar regions growth was uninterrupted. They noted (p. 472) that their results supported “the growing body of evidence that *Lystrosaurus* was endothermic”. More recently, nutrient foramina from dicynodonts have been found to indicate elevated metabolic rates consistent with endothermy (Knaus *et al*., 2021). Further support for endothermy among Dicynodontia comes from stable oxygen isotope analyses by Rey *et al.* (2017) who concluded that *Shansiodon* (Lower Triassic), *Lystrosaurus*, and *Moghreberia* were endothermic. Interestingly, they also concluded that *Dicynodon* (Late Permian) and *Oudenodon* were ectothermic, comparing oxygen isotope signatures with *Pareiasaurus*, which they assumed was ectothermic. However, on strong grounds we consider *Pareiasaurus* to have been endothermic (see above) and an osteological study by Faure-Brac & Cubo (2020) using eigenvector mapping listed *Oudenodon* as endothermic, along with *Lystrosaurus*. The evidence thus supports endothermy in Dicynodontia (Proxies 1, 3, 5).

**Therocephalia —** (Middle Permian–Middle Triassic). Hillenius (1992, 1994) described respiratory turbinates in the Late Permian therocephalian *Glanosuchus* and therefore deduced their tachymetabolism*.* By contrast, stable oxygen isotope analyses by Rey *et al.* (2017) implied that *Glanosuchus* was ectothermic. However, their conclusion was derived from comparing its oxygen isotope signature with that of *Pareiasaurus*, assuming *Pareiasaurus* to be ectothermic. Extensive FLB was reported in *Pristerognathus* (Middle Permian) (Ray *et al*., 2004), supporting the earlier finding by de Ricqlès (1969, referred to by Chinsamy-Turan & Ray, 2012) in this genus and in *Theriognathus* (Late Permian)*.* de Ricqlès (1969) also observed that Haversian bone was more extensive in the therocephalians than in gorgonopsians. Huttenlocker & Botha-Brink (2013) reported FLB and moderately fast but interrupted multi-year growth to large body size in Permian *Moschorhinus* and a change after the PTME to rapid sustained growth over a shorter period. Collectively, the evidence implies that tachymetabolic endothermy was common among therocephalians (Proxies 1, 6).

**Non-eucynodont Cynodontia —** (Late Permian–Early Triassic). As reviewed by Botha-Brink, Soares & Martinelli (2018), FLB appears to be typical across Cynodontia, including the early representatives, characteristically showing rapid bone deposition sustained through early to middle ontogeny. They considered this pattern of growth to be plesiomorphic for non-mammaliaform cynodonts, having been found in even the most basal members of the clade. A similar growth pattern is reported from *Procynosuchus* (Late Permian) (Ray *et al*., 2004), *Galesaurus* (Early Triassic) (Botha-Brink, Abdala & Chinsamy, 2012; E. Butler, Abdala & Both-Brink, 2019) and *Thrinaxodon* (Early Triassic) (Botha & Chinsamy, 2005; Botha-Brink *et al*., 2012). Structures in the nasal cavity of *Thrinaxodon* have been interpreted by Hillenius (1994) as respiratory turbinates. More recently, nutrient foramina from cynodonts have been found to indicate elevated metabolic rates consistent with endothermy (Knaus *et al*., 2021). Tachymetabolic endothermy thus was apparently widespread among early Cynodontia (Proxies 1, 3, 6).

**Cynognathia —** (Early Triassic–Early Jurassic). High growth rates indicated by osteohistology and other attributes that imply tachymetabolic endothermy have been reported in numerous cynognathians: *Cynognathus* (Cynognathidae, mid-Triassic), *Diademon* (Diademodontidae, mid-Triassic) (Botha & Chinsamy, 2001); *Trirachodon*, *Langbergia*, (Trirachodontidae, Early–mid-Triassic) (Botha & Chinsamy, 2004; Botha-Brink *et al.*, 2012); *Traversodon* (Traversidodontidae, mid–Late Triassic) (Chinsamy & Abdala, 2008) and *Exaeretodon* and *Protuberum* (Gomphodontosuchinae, Triassic) (Chinsamy & Abdala, 2008; Veiga, Botha-Brink & Soares, 2018). Additionally, respiratory turbinates were described in *Massetognathus* (Massetognathinae, mid–Late Triassic) by Hillenius (1994) and the conclusion that both *Diademodon* and *Cynognathus* were tachymetabolic endotherms is consistent with the findings of Rey *et al.* (2017) using a stable oxygen isotope approach. (Proxies 1, 5, 6)

**Probainognathia —** (Middle Triassic–Holocene/Anthropocene). High growth rates indicated by histological studies of bones that imply tachymetabolic endothermy have been reported in numerous probainognathians. Interpreting de Ricqlès (1969), including re-examining his images, Botha-Brink *et al.* (2012) wrote that the bone tissue of *Chiniquodon* (Chiniquodontidae) comprised moderate to highly vascularised FLB, probably lacking in LAGs and suggesting rapid and sustained growth. The same authors reported that *Trucidocynodon* (Ecteniniidae) and the tritylodontids (*Tritylodon*) all exhibit uninterrupted FLB, indicating sustained and rapid growth to adult size. Osteology of *Prozostrodon* and the tritheledontid *Irajatherium* revealed similar growth patterns, with fibrolamellar structure and indications of rapid early growth with annual interruptions later in ontogeny (Botha-Brink *et al.*, 2018). They also reported poorly developed FLB in an ulna of *Brasilodon quadrangularus* (Brasilodontidae) and some poorly developed FLB and other woven bone was found in a femur and a tibia of *Brasilitherium riograndensis*. This is less convincing than in the other families, but given the widespread nature of endothermy in Probainognathia, it is likely to characterise Brasilidontidae as well. (Proxy 1)

**REFERENCES**

Allen, D. (2003). When *Terrestrisuchus gracilis* reaches puberty it becomes *Saltoposuchus connectens*! *Journal of Vertebrate Paleontology* **23**, 29A.

Amiot, R., Lécuyer, C., Buffetaut, E., Escarguel, G., Fluteau, F. & Martineau, F. (2006). Oxygen isotopes from biogenic apatites suggest widespread endothermy in Cretaceous dinosaurs. *Earth and Planetary Science Letters* **246**, 41–54.

Anderson, K. L., Druckenmiller, P. S., Erickson, G. M. & Maxwell,E.E. (2018). Skeletal microstructure of *Stenopterygius quadriscissus* (Reptilia, Ichthyosauria) from the Posidonienschiefer (Posidonia Shale, Lower Jurassic) of Germany. *Palaeontology* **62**, 443–449.

Apaldetti, C., Martínez, R. N., Cerda, I. A., Pol, D. & Alcober, O. (2018). An early trend towards gigantism in Triassic sauropodomorph dinosaurs. *Nature Ecology and Evolution* **3**, 1227–1232.

Bailleul, A. M., O’Connor, J. & Schweitzer, M. H. (2019). Dinosaur paleohistology: review, trends and new avenues of investigation. *PeerJ* **7**, e7764.

Bakker, R. T. (1971). Dinosaur physiology and the origin of Mammals. *Evolution* **25**, 636–658.

Barrick, R. E., Showers, W. J. & Fischer, A. G. (1996). Comparison of thermoregulation of four ornithischian dinosaurs and a varanid lizard from the Cretaceous Two Medicine Formation: Evidence from oxygen isotopes. *PALAIOS* **11**, 295.

Becerra, M. G., Pol, D., Rauhut, O. W. M. & Cerda, I. A. (2016). New heterodontosaurid remains from the Cañadón Asfalto Formation: cursoriality and the functional importance of the pes in small heterodontosaurids. *Journal of Paleontology* **90**, 555–577.

Bennett, S. C. (1993). The ontogeny of *Pteranodon* and other pterosaurs. *Paleobiology* **19**, 92–106.

Benson, R. B. J. (2012). Interrelationships of basal synapsids: cranial and postcranial morphological partitions suggest different topologies, *Journal of Systematic Palaeontology*, **10**, 601–624.

Benton, M.J. (2020). The origin of endothermy in synapsids and archosaurs and arms races in the Triassic, *Gondwana Research* doi:10.1016/j.gr.2020.08.003

Bernard, A., Lécuyer, C., Vincent, P., Amiot, R., Bardet, N., Buffetaut, E., Cuny, G., Fourel, F., Martineau, F., Mazin, J.-M. & Prieur, A. (2010). Regulation of body temperature by some Mesozoic marine reptiles. *Science* **328**, 1379–1382.

Boitsova, E. A., Skutschas, P. P., Sennikov, A. G., Golubev, V. K., Masuytin, V. V. & Masuytina, O. A. (2019). Bone histology of two pareiasaurs from Russia (*Deltavjatia rossica* and *Scutosaurus karpinskii*) with implications for pareiasaurian palaeobiology. *Biological Journal of the Linnean Society* **128**, 289–310.

Botha, J. & Angielczyk, K. D. (2007). An integrative approach to distinguishing the Late Permian dicynodont species *Oudenodon bainii* and *Tropidostoma microtrema* (Therapsida: Anomodontia). *Palaeontology* **50**, 1175–1209.

Botha, J. & Chinsamy, A. (2001). Growth patterns from the bone histology of the cynodonts *Diademodon* and *Cynognathus*. *Journal of Vertebrate Paleontology* **20**, 705–711.

Botha, J. & Chinsamy, A. (2004). Growth and life habits of the Triassic cynodont *Trirachodon*, inferred from bone histology. *Acta Palaeontologica Polonica* **49**, 619–627.

Botha, J. & Chinsamy, A. (2005). Growth patterns of *Thrinaxodon liorhinus,* a non-mammalian cynodont from the Lower Triassic of South Africa. *Palaeontology* **48**, 385–394.

Botha-Brink, J., Abdala, F. & Chinsamy, A. (2012). The radiation and osteohistology of nonmammaliaform cynodonts. In *Forerunners of Mammals: radiation,histology and biology*. (ed A. Chinsamy-Turan), pp. 223–246. Indiana University Press, Bloomington.

Botha-Brink, J. & Angielczyk, K. D. (2010). Do extraordinarily high growth rates in Permo-Triassic dicynodonts (Therapsida, Anomodontia) explain their success before and after the end-Permian extinction? *Zoological Journal of the Linnean Society* **160**, 341–365.

Botha-Brink, J. & Smith, R. M. H. (2011). Osteohistology of the Triassic archosauromorphs *Prolacerta*, *Proterosuchus*, *Euparkeria*, and *Erythrosuchus* from the Karoo Basin of South Africa. *Journal of Vertebrate Paleontology* **31**, 1238–1254.

Botha-Brink, J. & Smith, R. M. H. (2012). Palaeobiology of Triassic procolophonids, inferred from bone microstructure. *Comptes Rendus Palevol* **11**, 419–433.

Botha-Brink, J., Soares, M. B. & Martinelli, A. G. (2018). Osteohistology of Late Triassic prozostrodontian cynodonts from Brazil. *PeerJ* **6**, e5029.

Brice, P. H. & Grigg, G. C. (in press). Modelling gigantothermy endorses constitutional endothermy of ichthyosaurs, mosasaurs and plesiosaurs (Sauropsida). In *Crocodylian Biology and Archosaurian Paleobiology*. (eds H. N. Woodward and J. O. Farlow). Indiana University Press, Bloomington, Indiana.

Butler, E., Abdala, F. & Botha-Brink J. (2019). Postcranial anatomy of the Early Triassic nonmammaliaform cynodont *Galesaurus planiceps* (Owen, 1859) from the Karoo Basin, South Africa. Palaeontology. Palaeontology **5**, 1–32.

Butler, R., Upchurch, P. & Norman, D. B. (2008). The phylogeny of the ornithischian dinosaurs. *Journal of Systematic Palaeontology* **6**, 1–40.

Butler, R. J., Jones, A. S., Buffetaut, E., Mandl, G. W., Scheyer, T. M. & Schultz, O. (2019). Description and phylogenetic placement of a new marine species of phytosaur (Archosauriformes: Phytosauria) from the Late Triassic of Austria. *Zoological Journal of the Linnean Society* **187**, 198–228.

Canoville, A. & Chinsamy, A. (2017). Bone microstructure of pareiasaurs (Parareptilia) from the Karoo Basin, South Africa: Implications for growth strategies and lifestyle habits. *The Anatomical Record* **300**, 1039–1066.

Canoville, A., Chinsamy-Turan, A., (2011). Growth patterns and palaeoecology of pareiasaurs (Parareptilia, Pareiasauridae) inferred from long bone histology and microanatomy. *Journal of Vertebrate Paleontology* **31** (Suppl. 3), 83.

Carrier, D. R. & Farmer, C. G. (2000*a*). The evolution of pelvic aspiration in archosaurs. *Paleobiology* **26**, 271–293.

Carrier, D. R. & Farmer, C. G. (2000*b*). The integration of ventilation and locomotion in archosaurs. *American Zoologist* **40**, 87–100.

Cerda, I. & Chinsamy, A. (2012). Biological implications of the bone microstructure of the Late Cretaceous ornithopod dinosaur *Gasparinisaura cincosaltensis.* *Journal of Vertebrate Paleontology* **32**, 355–368.

Cerda, I. A., de Gasparini, Z. B., Coria, R. A., Salgado, L., Reguero, M., Ponce, D., Gonzalez, R., Janello, J. M. & Moly, J. (2019). Paleobiological inferences for the Antarctic dinosaur *Antarctopelta oliveroi* (Ornithischia: Ankylosauria) based on bone histology of the holotype. *Cretaceous Research* **103**, 104171.

Chinsamy, A. & Abdala, F. (2008). Paleobiological implications of the bone microstructure of South American traversodontids (Therapsida: Cynodontia). *South African Journal of Science* **104**, 225–230.

Chinsamy, A., Codorniú, L. & Chiappe, L. (2008). Developmental growth patterns of the filter-feeder pterosaur, *Pterodaustro guinazui*. *Biology Letters* **4**, 282–285.

Chinsamy-Turan, A. & Ray, S. (2012). Bone histology of some therocephalians and gorgonopsians, and evidence of bone degradation by fungi. In *Forerunners of Mammals: Radiation,Histology, Biology*. (ed A. Chinsamy-Turan), pp. 199–222. Indiana University Press., Bloomington.

Claessens, L. P. A. M. (2004). Dinosaur gastralia: Origin, morphology, and function. *Journal of Vertebrate Paleontology* **24**, 89–106.

Claessens, L. P. A. M., O'Connor, P. M. & Unwin, D. M. (2009). Respiratory evolution facilitated the origin of pterosaur flight and aerial gigantism. *PLoS ONE* **4**, e4497.

Cubo, J. & Jalil, N.-E. (2019). Bone histology of *Azendohsaurus laaroussii*: Implications for the evolution of thermometabolism in Archosauromorpha. *Paleobiology* **45**, 317–330.

Cubo, J., Le Roy, N., Martinez-Maza, C. & Montes, L. (2012). Paleohistological estimation of bone growth rate in extinct archosaurs. *Paleobiology* **38**, 335–349.

Cubo, J., Sena, M. V. A., Aubier, P., Houee, G., Claisse, P., Faure-Brac, M. G., Allain, R., Andrade, R. L. P., Sayão, J. M. & Oliveira, G. R. (2020). Were Notosuchia (Pseudosuchia: Crocodylomorpha) warm-blooded? A palaeohistological analysis suggests ectothermy. Biological Journal of the Linnean Society **131**,154–162.

Curry Rogers, K. & Kulik, Z. (2018). Osteohistology of *Rapetosaurus krausei* (Sauropoda: Titanosauria) from the Upper Cretaceous of Madagascar. *Journal of Vertebrate Paleontology* e1493689.

Dawson, R. R., Field, D. J., Hull, P. M., Zelenitsky, D. K., Therrien, F. & Affek, H. P. (2020). Eggshell geochemistry reveals ancestral metabolic thermoregulation in Dinosauria. *Science Advances* **6**, eaax9361.

de Buffrénil, V. & Mazin, J-M. (1990). Bone histology of the ichthyosaurs: comparative data and functional interpretation. *Palaeobiology* **16**, 435–447.

de Ricqlès, A. (1969). Recherches paléohistologiques sur les os longs des Tétrapodes: Quelques observations sur la structure des os longs des Thériodontes. *Annales de Paléontologie* **55**, 1–52.

de Ricqlès, A. (1978*a*). Recherches paléohistologiques sur les os longs des tétrapodes. VII.—Sur la classification, la signification fonctionnelle et l’histoire des tissus osseux des tétrapodes. Troisième partie. Evolution: Considérations phylogénétiques. *Annales de Paléontologie* **64**, 85–111.

de Ricqlès, A. (1978*b*). Recherches paléohistologiques sur les os longs des tétrapodes. VII—Sur la classification, la signification fonctionnelle et l'histoire des tissus osseux des tétrapodes (Troisième partie: Les problèmes du déterminisme des types de tissus osseux). *Annalés de Paleontologie* **64**, 153–184.

de Ricqlès, A., Padian, K. & Horner, J. R. (2003). On the bone histology of some Triassic pseudosuchian archosaurs and related taxa. *Annales de Paléontologie* **89**, 67–101.

de Ricqlès, A., Padian, K., Horner, J. R. & Francillon-Vieillot, H. (2000). Palaeohistology of the bones of pterosaurs (Reptilia : Archosauria): anatomy, ontogeny, and biomechanical implications. *Zoological Journal of the Linnean Society* **129**, 349–385.

de Ricqlès, A. J., Padian, K., Knoll, F. & Horner, J. R. (2008). On the origin of high growth rates in archosaurs and their ancient relatives: Complementary histological studies on Triassic archosauriforms and the problem of a “phylogenetic signal” in bone histology. *Annales de Paléontologie* **94**, 57–76.

Eagle, R. A., Enriquez, M., Grellet-Tinner, G., Pérez-Huerta, A., Hu, D., Tütken, T., Montanari, S., Loyd, S. J., Ramirez, P., Tripati, A. K., Kohn, M. J., Cerling, T. E., Chiappe, L. M. & Eiler, J. M. (2015). Isotopic ordering in eggshells reflects body temperatures and suggests differing thermophysiology in two Cretaceous dinosaurs. *Nature Communications* **6**, 8296.

Eagle, R. A., Tütken, T., Martin, T. S., Tripati, A. K., Fricke, H. C., Connely, M., Cifelli, R. L. & Eiler, J. M. (2011). Dinosaur body temperatures determined from isotopic (^13^C-^18^O) ordering in fossil biominerals. *Science* **333**, 443–445.

Enlow, D. H. & Brown, S. O. (1957). A comparative histological study of fossil and recent bone tissues. Part II. *Texas Journal of Science* **9**, 186–204.

Erickson, G. M. & Druckenmiller, P. S. (2011). Longevity and growth rate estimates for a polar dinosaur: a *Pachyrhinosaurus* (Dinosauria: Neoceratopsia) specimen from the North Slope of Alaska showing a complete developmental record. *Historical Biology* **23**, 327–334.

Erickson, G. M. & Tumanova, T. A. (2000). Growth curve of *Psittacosaurus mongoliensis* Osborn (Ceratopsia: Psittacosauridae) inferred from long bone histology. *Zoological Journal of the Linnean Society* **130**, 551–566.

Estefa, J., Klembara, J., Tafforeau, P. & Sanchez, S. (2020). Limb-bone development of seymouriamorphs: Implications for the evolution of growth strategy in stem amniotes. *Frontiers in Earth Science* **8,** 97. doi: 10.3389/feart.2020.00097

Ezcurra, M. D., Montefeltro, F. & Butler, R. J. (2016). The early evolution of rhynchosaurs. *Frontiers in Ecology and Evolution* **3**, 142. [doi.org/10.3389/fevo.2015.00142](https://doi.org/10.3389/fevo.2015.00142)

Ezcurra, M. D., Scheyer, T. M. & Butler, R. J. (2014). The origin and early evolution of Sauria: reassessing the Permian saurian fossil record and the timing of the crocodile-lizard divergence. *Plos One* **9**, e89165.

Farias, B. D. M., Schultz, C. M. & Soares, M. B. (2019). Bone microstructure of the pareiasaur *Provelosaurus americanus* from the Middle Permian of southern Brazil. *Historical Biology* **33**, 1–12.

Farlow, J. O., Dodson, P. & Chinsamy, A. (1995). Dinosaur biology. *Annual Review of Ecology and Systematics* **26**, 445–471.

Farmer, C. G. & Sanders, K. (2010). Unidirectional airflow in the lungs of alligators. *Science* **327**, 338–340.

Faure-Brac, M. G. & Cubo, J. (2020). Were the synapsids primitively endotherms? A palaeohistological approach using phylogenetic eigenvector maps. *Philosophical Transactions of the Royal Society B: Biological Sciences* **375**, 20190138.

Fleischle, C. V., Wintrich, T. & Sander, P. M. (2018). Quantitative histological models suggest endothermy in plesiosaurs. *PeerJ* **6**, e4955.

Ford, D.P., Benson, R.B.J. (2020). The phylogeny of early amniotes and the affinities of Parareptilia and Varanopidae. *Nature Ecology and Evolution* **4,**57–65.

Gauthier, J. A., Nesbitt, S. J., Schachner, E. R., Bever, G. S. & Joyce, W. G. (2011). The bipedal stem crocodilian *Poposaurus gracilis*: Inferring function in fossils and innovation in archosaur locomotion. *Bulletin of the Peabody Museum of Natural History* **52**, 107–126.

Green, J. L., Schweitzer, M. H. & Lamm, E.-T. (2010). Limb bone histology and growth in *Placerias hesternus* (Therapsida: Anomodontia) from the Upper Triassic of North America. *Palaeontology* **53**, 347–364.

Harrell, L., Perez-Huerta, A. & Suarez, C. (2016). Endothermic mosasaurs? Possible thermoregulation of Late Cretaceous mosasaurs (Reptilia, Squamata) indicated by stable oxygen isotopes in fossil bioapatite in comparison with coeval marine fish and pelagic seabirds. *Palaeontology* **59**, 351–363.

Hayashi, S., Carpenter, K. & Suzuki, D. (2009). Different growth patterns between the skeleton and osteoderms of *Stegosaurus* (Ornithischia: Thyreophora). *Journal of Vertebrate Paleontology* **29**, 123–131.

Heingård, M. (2014). *Long bone and vertebral microanatomy and osteo-histology of ’Platecarpus’ ptychodon (Reptilia, Mosasauridae) - implications for marine adaptations.* Dissertation. University of Lund.

Hillenius, W. J. (1992). The evolution of nasal turbinates and mammalian endothermy. *Paleobiology* **18**, 17–29.

Hillenius, W. J. (1994). Turbinates in therapsids: Evidence for Late Permian origins of mammalian endothermy. *Evolution* **48**, 207–229.

Hoffman, D. K., Heckert, A. B. & Zanno, L. E. (2018). Disparate growth strategies within Aetosauria: novel histologic data from the aetosaur *Coahomasuchus chathamensis*. *The Anatomical Record* **302**, 1504–1515.

Hofmann, R. & Sander, P. M. (2014). The first juvenile specimens of *Plateosaurus engelhardti* from Frick, Switzerland: isolated neural arches and their implications for developmental plasticity in a basal sauropodomorph. *PeerJ* **2**, e458.

Horner, J. R., de Ricqlès, A. J. & Padian, K. (2000). Long bone histology of the hadrosaurid dinosaur *Maiasaura peeblesorum*: growth dynamics and physiology based on an ontogenetic series of skeletal elements. *Journal of Vertebrate Paleontology* **20**, 115–129.

Horner, J. R., de Ricqlès, A. J., Padian, K. & Scheetz, R. D. (2009). Comparative long bone histology and growth of the “hypsilophodontid” dinosaurs *Orodromeusmakelai*, *Dryosaurusaltus*, and *Tenontosaurustillettii* (Ornithischia: Euornithopoda). *Journal of Vertebrate Paleontology* **29**, 734–747.

Houssaye, A., Lindgren, J., Pellegrini, R., Lee, A. H., Germain, D. & Polcyn, M. J. (2013). Microanatomical and histological features in the long bones of mosasaurine mosasaurs (Reptilia, Squamata) – Implications for aquatic adaptation and growth rates. *Plos One* **8**, e76741.

Houssaye, A., Scheyer, T, m., Kolb, C., Fischer, V. & Sander, P. M. (2014). A new look at ichthyosaur long bone microanatomy and histology: Implications for their adaptation to an aquatic life. *Plos One* **9**, e95637.

Huttenlocker, A. K. & Botha-Brink, J. (2013). Body size and growth patterns in the therocephalian *Moschorhinus kitchingi* (Eutheriodontia) before and after the end-Permian extinction in South Africa. *Paleobiology* **39**, 253–277.

Huttenlocker, A. K., Mazierski, D. & Reisz, R. R. (2011). Comparative osteohistology of hyperelongate neuralspines in the Edaphosauridae (Amniota: Synapsida). *Palaeontology* **54**, 573-590.

Huttenlocker, A. K., Rega, E. & Sumida, S. (2010). Comparative anatomy and osteohistology of hyperelongate neural spines in the sphenacodontids *Sphenacodon* and *Dimetrodon* (Amniota: Synapsida). *Journal of Morphology* **271**, 1407–1421.

Huttenlocker, A. K. & Shelton, C. D. (2020). Bone histology of varanopids (Synapsida) from Richards Spur, Oklahoma, sheds light on growth patterns and lifestyle in early terrestrial colonizers. *Philosophical Transactions of the Royal Society B: Biological Sciences* **375**, 20190142.

Jenkins, H. S. & Pratson, L. F. (2005). Evidence for endothermy in pterosaurs based on flight capability analyses In *American Geophysical Union*. American Geophysical Union, San Francisco.

Klein, N. (2010). Long bone histology of Sauropterygia from the Lower Muschelkalk of the Germanic Basin provides unexpected implications for phylogeny. *Plos One* **5**, e11613.

Klein, N., Foth, C. & Schoch, R. R. (2017). Preliminary observations on the bone histology of the Middle Triassic pseudosuchian archosaur *Batrachotomus kupferzellensis* reveal fast growth with laminar fibrolamellar bone tissue. *Journal of Vertebrate Paleontology* **37**, e1333121.

Klein, N., Houssaye, A., Neenan, J. M. & Scheyer, T. M. (2015). Long bone histology and microanatomy of Placodontia (Diapsida: Sauropterygia). *Contributions to Zoology* **84**, 59–84.

Knaus, P.L., Van Heteren, A.H., Lungmus, J.K. & Sander, P.M. (2021). Higher blood flow into the femur indicates elevated aerobic capacity in synapsids since the reptile-mammal split. Frontiers in Ecology and Evolution (in press 751238)

Knoll, F., Padian, K. & Ricqlès, A. (2010). Ontogenetic change and adult body size of the early ornithischian dinosaur *Lesothosaurus diagnosticus*: Implications for basal ornithischian taxonomy. *Gondwana Research* **17**, 171–179.

Kolb, C., Sánchez-Villagra, M. R. & Scheyer, T. M. (2011). The palaeohistology of the basal ichthyosaur *Mixosaurus* (Ichthyopterygia, Mixosauridae) from the Middle Triassic: Palaeobiological implications. *Comptes Rendus Palevol* **10**, 403–411.

Krahl, A., Klein, N. & Sander, P. M. (2013). Evolutionary implications of the divergent long bone histologies of *Nothosaurus* and *Pistosaurus* (Sauropterygia, Triassic). *Biomed Central Evolutionary Biology* **13**, 1-23.

Lacovara, K. J., Lamanna, M. C., L.M., I., Poole, J. C., Schroeter, E. R., Ullmann, P. V., Voegele, K. K., Boles, Z. M., Carter, A. M., Fowler, E. K., Egerton, V. M., Moyer, A. E., Coughenour, C. L., Schein, J. P., Harris, J. D., Martínez, R. D., *et al*. (2014). A gigantic, exceptionally complete titanosaurian sauropod dinosaur from Southern Patagonia, Argentina. *Science Reports* **4**, 6196.

Laurin, M. & de Buffrénil, V. (2016). Microstructural features of the femur in early ophiacodontids: A reappraisal of ancestral habitat use and lifestyle of amniotes. *Comptes Rendus Palevol* **15**, 115–127.

Legendre, L. J., Guenard, G., Botha-Brink, J. & Cubo, J. (2016). Palaeohistological evidence for ancestral high metabolic rate in archosaurs. *Systematic Biology* **65**, 989–996.

Legendre, L. J., Segalen, L. & Cubo, J. (2013). Evidence for high bone growth rate in *Euparkeria* obtained using a new paleohistological inference model for the humerus. *Journal of Vertebrate Paleontology* **33**, 1343–1350.

Lindgren, J., Sjövall, P., Thiel, V., Zheng, W., Ito, S., Wakamatsu, K., Hauff, R., Kear, B. P., Engdahl, A., Alwmark, C., Eriksson, M. E., Jarenmark, M., Sachs, S., Ahlberg, P. E., Marone, F., *et al*. (2018). Soft-tissue evidence for homeothermy and crypsis in a Jurassic ichthyosaur. *Nature* **564**, 359–365.

Looy, C., Ranks, S., Chaney, D., Sanchez, S., Steyer, J. S., Smith, R., Sidor, C., Myers, T., Ide, O. & Tabor, N. (2016). Biological and physical evidence for extreme seasonality in central Permian Pangea. *Palaeogeography, Palaeoclimatology, Palaeoecology* **451**, 210–226.

MacDougall, M.J., Modesto, S.P., Brocklehurst, N., Verrière, A., Reisz, R.R. & Fröbisch, J. (2018) Commentary: A reassessment of the taxonomic position of mesosaurs, and a surprising phylogeny of early amniotes. *Frontiers in Earth Science* **6**, 99.

Marsà, J. A. G., Agnolín, F. L. & Novas, F. (2019). Bone microstructure of *Lewisuchus admixtus* Romer, 1972 (Archosauria, Dinosauriformes). *Historical Biology* **31**, 157–162.

Mukherjee, D. (2015). New insights from bone microanatomy of the Late Triassic *Hyperodapedon* (Archosauromorpha, Rhynchosauria): implications for archosauromorph growth strategy. *Palaeontology* **58**, 313–339.

Nakajima, Y., Houssaye, A. & Endo, H. (2014). Osteohistology of the Early Triassic ichthyopterygian reptile *Utatsusaurus hataii*: Implications for early ichthyosaur biology. *Acta Palaeontologica Polonica* **59**, 343–352.

Nesbitt, S. J. (2007). The anatomy of *Effigia okeeffeae* (Archosauria, Suchia), theropod-like convergence, and the distribution of related taxa. *Bulletin of the American Museum of Natural History* **302**, 1–84.

Nesbitt, S. J. (2011). The early evolution of archosaurs: relationships and the origin of major clades. *Bulletin of the American Museum of Natural History* **352**, 1–192.

O’Keefe, F. R., Sander, P. M., Wintrich, T. & Werning, T. (2019). Ontogeny of polycotylid long bone microanatomy and histology. [*Integrative Organismal Biology* **1**](file:///C:\Users\zlggrigg\Documents\Working%20Docs\Echidnas,%20hibernation%20&%20evoln%20endothermy%20&%20reptile%20TR\homology%20of%20endothermy%20in%20mammals%20and%20birds\Integrative%20Organismal%20Biology%201)**,** 1–26**.**

Olivier, C., Houssaye, A., Jalil, N.-E. & Cubo, J. (2017). First palaeohistological inference of resting metabolic rate in an extinct synapsid, *Moghreberia nmachouensis* (Therapsida: Anomodontia). *Biological Journal of the Linnean Society* **121**, 409–419.

Padian, K. (1983). Osteology and functional morphology of *Dimorphodon macronjyx* (Buckland) (Pterosauria: Rhamphorhynchoidea) based on new material in the Yale Peabody Museum. *Postilla (Peabody Museum of Natural History)* **189**, 1–44.

Padian, K. & de Ricqlès, A. J. (2020). Inferring the physiological regimes of extinct vertebrates: methods, limits and framework. *Philosophical Transactions of the Royal Society of London. Series B, Biological Sciences* **375**, 20190147.

Padian, K., Horner, J. R. & de Ricqlès, A. J. (2004). Growth in small dinosaurs and pterosaurs: the evolution of archosaurian growth strategies. *Journal of Vertebrate Paleontology* **24**, 555–571.

Paladino, F. V., Spotila, J. R. & Dodson, P. (1997). A blueprint for giants: Modelling the physiology of large dinosaurs. In *The complete dinosaur* (ed. J. O. Farlow and M. K. Brett-Surman), pp. 491–504. Indiana University Press, Bloomington.

Paul, G. S. (2010). *The Princeton Field Guide to Dinosaurs*. Princeton University Press, New Jersey, USA.

Pontzer, H., Allen, V. & Hutchinson, J. R. (2009). Biomechanics of running indicates endothermy in bipedal dinosaurs. *Plos One* **4**, e7783.

Prondvai, E. (2014). Comparative bone histology of rhabdodontid dinosaurs. *Palaeovertebrata* **38**, e1.

Ray, S., Bandyopadhyay, S. & Bhawal, D. (2009). Growth patterns as deduced from bone microstructure of some selected neotherapsids with special emphasis on dicynodonts: phylogenetic implications. *Palaeoworld* **18**, 53–66.

Ray, S., Botha, J. & Chinsamy, A. (2004). Bone histology and growth patterns of some nonmammalian therapsids. *Journal of Vertebrate Palaeontology* **24**, 634–648.

Ray, S., Chinsamy, A. & Bandyopadhyay, S. (2005). *Lystrosaurus murrayi* (Therapsida, Dicynodontia): bone histology, growth and lifestyle adaptations. *Palaeontology* **48**, 1169–1185.

Redelstorff, R., Hübner, T. R., Chinsamy, A. & Sander, P. M. (2013). Bone histology of the stegosaur *Kentrosaurus aethiopicus* (Ornithischia: Thyreophora) from the Upper Jurassic of Tanzania. *The Anatomical Record* **296**, 933–952.

Reid, R. E. H. (1997). Dinosaurian physiology: the case for ‘intermediate’ dinosaurs; pp. 449–473 in J. O. Farlow and M. K. Brett-Surman (eds.), The Complete Dinosaur. Indiana University Press, Bloomington.

Rey, K., Amiot, R., Fourel, F., Abdala, F., Fluteau, F., Jalil, N.-E., Liu, J., Rubidge, B. S., Smith, R. M. H., Steyer, J. S., Viglietti, P. A., Wang, X. & Lécuyer, C. (2017). Oxygen isotopes suggest elevated thermometabolism within multiple Permo-Triassic therapsid clades. *eLife* **6**, e28589.

Rey, K., Day, M. O., Amiot, R., Fourel, F., Luyt, J., Van den Brandt, M. J., Lécuyer, C. & Rubidge, B. S. (2020). Oxygen isotopes and ecological inferences of Permian (Guadalupian) tetrapods from the main Karoo Basin of South Africa. *Palaeogeography, Palaeoclimatology, Palaeoecology* **538**, 109485. doi: 10.1016/j.palaeo.2019.109485.

Rimblot-Baly, F., de Ricqlès, A. & Zylberberg, L. (1995). Analyse paléohistologique d’une série de croissance partielle chez *Lapparentosaurus madagascariensis* (Jurassique moyen): Essai sur la dynamique de croissance d’un dinosaure sauropode. *Annales de Paléontologie (Invertebres-Vertebres)* **81**, 49–86.

Sander, P. M. & Klein, N. (2005). Developmental plasticity in the life history of a prosauropod dinosaur. *Science* **310**, 1800–1802.

Sander, P. M., Klein, N., Stein, K. & Wings, O. (2011). Sauropod bone histology and its implications for sauropod biology. In *Biology of the sauropod dinosaurs: Understanding the life of giants* (eds N. Klein, K. Remes, C. T. Gee and P. M. Sander), pp. 276–302. Indiana University Press, Bloomington.

Schachner, E. R. (2010). *Anatomical reconstructions of respiratory morphology and hindlimb musculature in Poposaurus gracilis (Archosauria Poposauroidea) and related dinosauriformes.* Dissertation. University of Pennsylvania.

Schachner, E. R., Farmer, C. G., McDonald, A. T. & Dodson, P. (2011). Evolution of the dinosauriform respiratory apparatus: new evidence from the postcranial skeleton. *Anatomical Record* **294**, 1532–1547.

Scheyer, T. M., Desojo, J. B. & Cerda, I. A. (2014). Bone histology of phytosaur, aetosaur, and other archosauriform osteoderms (Eureptilia, Archosauromorpha). *The Anatomical Record* **297**, 240–260.

Schroeter, E., Boles, Z. & Lacovara, K. (2011). The histology of a massive titanosaur from Argentina and implications for maximum size. In *Society of Vertebrate Paleontology Meeting Abstracts 189*. Society of Vertebrate Paleontology.

Sereno, P. C. (1997 ). The origin and evolution of dinosaurs. *Annual Review of Earth and Planetary Sciences* **25**, 435–489.

Seymour, R. S. (1976). Dinosaurs, endothermy and blood pressure. *Nature* **262**, 207–208.

Seymour, R. S. (2013). Maximal aerobic and anaerobic power generation in large crocodiles versus mammals: implications for dinosaur gigantothermy. *Plos One* **8**, e69361.

Seymour, R. S. (2016). Cardiovascular physiology of dinosaurs. *Physiology (Bethesda)* **31**, 430–441.

Seymour, R. S. (in press). Physiology and anatomy of extant crocodiles as windows to pseudosuchian evolution. In *Crocodylian Biology and Archosaurian Paleobiology*. (eds H. N. Woodward and J. O. Farlow). Indiana University Press, Bloomington, Indiana.

Seymour, R. S., Bennett-Stamper, C. L., Johnston, S. D., Carrier, D. R. & Grigg, G. C. (2004). Evidence for endothermic ancestors of crocodiles at the stem of archosaur evolution. *Physiological and Biochemical Zoology* **77**, 1051–1067.

Seymour, R. S., Ezcurra, M., Henderson, D., Jones, M. E., Maidment, S. C., Miller, C. V., Nesbitt, S. J., Schwarz, D., Sullivan, C. & Wilberg, E. (2019). Large nutrient foramina in fossil femora indicate intense locomotor and metabolic activity in Triassic archosauromorphs and the pseudosuchian lineage. p.190. In *Society for Vertebrate Paleontology Meeting Abstracts*. Society for Vertebrate Paleontology, Brisbane.

Seymour, R. S., Smith, S. L., White, C. R., Henderson, D. M. & Schwarz-Wings, D. (2012). Blood flow to long bones indicates activity metabolism in mammals, reptiles and dinosaurs. *Proceedings of the Royal Society of London. Series B, Biological Sciences* **279**, 451–456.

Shelton, C. D. (2014). *Origins of endothermy in the mammalian lineage: the evolutionary beginning of fibro-lamellar bone in the “mammal-like” reptiles.* Dissertation. Rheinischen Friedrich-Wilhelms-Universität Bonn.

Shelton, C. D. & Sander, P. M. (2017). Long bone histology of *Ophiacodon* reveals the geologically earliest occurrence of fibrolamellar bone in the mammalian stem lineage. *Comptes Rendus Palevol* **16**, 397–424.

Shelton, C., Sander, P. M., Stein, K. & Winkelhorst, H. (2012). Long bone histology indicates sympatric species of *Dimetrodon* (Lower Permian,Sphenacodontidae). *Earth and Environmental Science Transactions of the Royal Society of Edinburgh* **103**, 217–236.

Sookias, R. B. (2016). The relationships of the Euparkeriidae and the rise of Archosauria. *Royal Society Open Science* **3,** 150674. doi: 10.1098/rsos.150674.

Steel, L. (2008). The palaeohistology of pterosaur bone: an overview. *Zitteliana* **28**, 109–126.

Stein, M., Hayashi, S. & Sander, M. (2013). Long bone histology and growth patterns in ankylosaurs: Implications for life history and evolution. *Plos One* **8**, e68590.

Stein, K. & Prondvai, E. (2014). Rethinking the nature of fibrolamellar bone: an integrative biological revision of sauropod plexiform bone formation. *Biological Reviews* **89**, 24–47.

Sulej, T. & Niedźwiedzki, G. (2019). An elephant-sized Late Triassic synapsid with erect limbs. *Science* **363**, 78–80.

Sumida, S. S. & Modesto, S. (2001). A phylogenetic perspective on locomotory strategies in early amniotes. *American Zoologist* **41**, 586–597.

Taborda, J. R. A., Cerda, I. A. & Desojo, J. B. (2013). Growth curve of *Aetosauroides scagliai* Casamiquela 1960 (Pseudosuchia: Aetosauria) inferred from osteoderm histology. In *Anatomy, phylogeny and palaeobiology of early archosaurs and their kin.* (Volume 379, eds S. J. Nesbitt, J. B. Desojo and R. B. Irmis), pp. 413–423. Geological Society, Special Publications. London.

Thulborn, R. A. (1972). The postcranial skeleton of the Triassic ornithischian dinosaur *Fabrosaurus australis*. *Palaeontology* **15**, 29–60.

Turner, M. L., Tsuji, L. A., Ide, O. & Sidor, C. A. (2015). The vertebrate fauna of the upper Permian of Niger—IX. The appendicular skeleton of *Bunostegos akokanensis* (Parareptilia: Pareiasauria). *Journal of Vertebrate Paleontology* **35**, e994746.

Veiga, F. H., Botha-Brink, J. & Soares, M. B. (2018). Osteohistology of the non-mammaliaform traversodontids *Protuberum cabralense* and *Exaeretodon riograndensis* from southern Brazil. *Historical Biology* **31**, 1231–1241

Veiga, F. H., Soares, M. B. & Sayao, J. M. (2015). Osteohistology of hyperodapedontine rhynchosaurs from the Upper Triassic of Southern Brazil. *Acta Palaeontologica Polonica* **60**, 829–836.

Wedel, M. J. (2003*a*). The evolution of vertebral pneumaticity in sauropod dinosaurs. *Journal of Vertebrate Paleontology* **23**, 344–357.

Wedel, M. J. (2003*b*). Vertebral pneumaticity, air sacs, and the physiology of sauropod dinosaurs. *Paleobiology* **29**, 243–255.

Wedel, M. J. (2005). Postcranial skeletal pneumaticity in sauropods and its implications for mass estimates. In *The Sauropods: Evolution and Paleobiology*. (eds K. A. Curry-Rogers and J. A. Wilson), pp. 201–228. University of California Press, Berkeley.

Weinbaum, J. C. (2013). Postcranial skeleton of *Postosuchus kirkpatricki* (Archosauria: Paracrocodylomorpha), from the upper Triassic of the United States. In *Anatomy, phylogeny and palaeobiology of early archosaurs and their kin. Special Publications* (Volume 379, eds S. J. Nesbitt, J. B. Desojo and R. B. Irmis), pp. 525–553. Geological Society, London.

Werning, S. (2012). The ontogenetic osteohistology of *Tenontosaurus tilletti*. *Plos One* **7**, e33539.

Werning, S. & Nesbitt, S. J. (2016). Bone histology and growth in *Stenaulorhynchus stockleyi* (Archosauromorpha: Rhynchosauria) from the Middle Triassic of the Ruhuhu Basin of Tanzania. *Comptes Rendus Palevol* **15**, 163–175.

Whitney, M. R. & Sidor, C. A. (2020). Evidence of torpor in the tusks of *Lystrosaurus* from the Early Triassic of Antarctica. *Communications Biology* **3**, 471.

Wiffen, J., de Buffrenil, V., de Ricqlès, A., & Mazin, J.-M. (1995). Ontogenetic evolution of bone structure in Late Cretaceous Plesiosauria from New Zealand. *Geobios* **28**, 625–640.

Wintrich, T., Hayashi, S., Houssaye, A., Nakajima, Y. & Sander, P. M. (2017). A Triassic plesiosaurian skeleton and bone histology inform on evolution of a unique body plan and survival of end-Triassic extinctions. *Science Advances* **3**, e1701144.

Wintrich, T. & Sander, P. M. (2019). Inferences on plesiosaurian metabolic rate and vascular system from nutrient foramina in long bones. p. 220. In *Society for Vertebrate Paleontology Meeting Abstracts*. Society for Vertebrate Paleontology, Brisbane.

Woodward, H. N., Freedman Fowler , E. A., Farlow, J. O. & Horner, J. R. (2015). *Maiasaura*, a model organism for extinct vertebrate population biology: a large sample statistical assessment of growth dynamics and survivorship. *Paleobiology* **41**, 503–527.

Woodward, H. N., Rich, T. H., Chinsamy, A. & Vickers-Rich, P. (2011). Growth dynamics of Australia's polar dinosaurs. *Plos One* **6**, e23339.

Woodward, H. N., Rich, T. N. & Vickers-Rich, P. (2018). The bone microstructure of polar “hypsilophodontid” dinosaurs from Victoria, Australia. *Scientific Reports* **8**, 1162.

Zhao, Q., Benton, M. J., Hayashi, S. & Xu, X. (2019). Ontogenetic stages of ceratopsian dinosaur *Psittacosaurus* in bone histology. *Acta Palaeontologica Polonica* **64**, 323–334.

Zheng, X.-T., You, H.-L., Xu, X., Dong, Z.-M. & You, H.-L. (2009). An Early Cretaceous heterodontosaurid dinosaur with filamentous integumentary structures. *Nature* **458**, 333–336.
